# Digital tools for the recruitment and retention of participants in randomised controlled trials: a systematic map

**DOI:** 10.1186/s13063-020-04358-3

**Published:** 2020-06-05

**Authors:** Geoff K. Frampton, Jonathan Shepherd, Karen Pickett, Gareth Griffiths, Jeremy C. Wyatt

**Affiliations:** 1 Southampton Health Technology Assessments Centre (SHTAC), Wessex Institute, Faculty of Medicine, University of Southampton, Alpha House, Southampton Science Park, Southampton, SO16 7NS UK; 2 Wessex Institute, Faculty of Medicine, University of Southampton, Alpha House, Southampton Science Park, Southampton, SO16 7NS UK; 3 Southampton Clinical Trials Unit, University of Southampton and Southampton University Hospital NHS Foundation Trust, Southampton General Hospital, Southampton, SO16 6YD UK

**Keywords:** Clinical trial management, Clinical trial efficiency, Recruitment strategies, Retention strategies, Participant identification and recruitment, Online recruitment, Participant retention, Digital tools, Electronic tools, Systematic map

## Abstract

**Background:**

Recruiting and retaining participants in randomised controlled trials (RCTs) is challenging. Digital tools, such as social media, data mining, email or text-messaging, could improve recruitment or retention, but an overview of this research area is lacking. We aimed to systematically map the characteristics of digital recruitment and retention tools for RCTs, and the features of the comparative studies that have evaluated the effectiveness of these tools during the past 10 years.

**Methods:**

We searched Medline, Embase, other databases, the Internet, and relevant web sites in July 2018 to identify comparative studies of digital tools for recruiting and/or retaining participants in health RCTs. Two reviewers independently screened references against protocol-specified eligibility criteria. Included studies were coded by one reviewer with 20% checked by a second reviewer, using pre-defined keywords to describe characteristics of the studies, populations and digital tools evaluated.

**Results:**

We identified 9163 potentially relevant references, of which 104 articles reporting 105 comparative studies were included in the systematic map. The number of published studies on digital tools has doubled in the past decade, but most studies evaluated digital tools for recruitment rather than retention. The key health areas investigated were health promotion, cancers, circulatory system diseases and mental health. Few studies focussed on minority or under-served populations, and most studies were observational. The most frequently-studied digital tools were social media, Internet sites, email and tv/radio for recruitment; and email and text-messaging for retention. One quarter of the studies measured efficiency (cost per recruited or retained participant) but few studies have evaluated people’s attitudes towards the use of digital tools.

**Conclusions:**

This systematic map highlights a number of evidence gaps and may help stakeholders to identify and prioritise further research needs. In particular, there is a need for rigorous research on the efficiency of the digital tools and their impact on RCT participants and investigators, perhaps as studies-within-a-trial (SWAT) research. There is also a need for research into how digital tools may improve participant retention in RCTs which is currently underrepresented relative to recruitment research.

**Registration:**

Not registered; based on a pre-specified protocol, peer-reviewed by the project’s Advisory Board.

## Background

### The problem: inadequate patient recruitment and retention

Recruiting and retaining participants in clinical and health studies is a key determinant of research efficiency, but is highly challenging [1, 2], and poor rates of recruitment to randomised controlled trials (RCTs) are commonly reported [3]. In reviews of clinical trials funded by the UK Medical Research Council and/or the National Institute for Heath Research (NIHR) Health Technology Assessment (HTA) programme [4–7] the proportion of trials achieving their original recruitment target ranged from only 31% (of 114 trials that recruited between 1994 and 2002) [4] to 60% (of 151 trials published from 2004 to April 2016) [7]. Although there appears to have been some improvement over time, the latest data [7] suggest that around 40% of trials may still fail to reach their intended enrolment numbers, despite considerable interest in approaches that might improve study recruitment [2, 8].

Once patients have been recruited into clinical or health studies the rates at which they are retained in the studies can be highly variable, depending on the nature of the population, condition, intervention, comparator and outcomes. For example, estimates from trials in Alzheimer’s disease [9] suggest that that there is an average dropout rate of 30% across trials and 85% of trials fail to retain enough patients [10], whilst in a systematic review of 87 RCTs of asthma inhalers we found that dropout rates ranged from 0% to over 40% [11].

Consequences of inadequate trial recruitment include: reduced statistical power of trials due to inadequate sample size, increasing the risk of type II error (failure to detect real treatment effects) [12]; wasting patients’ and investigators’ time and money (e.g. 481 trials that closed during 2011 due to inadequate recruitment had already involved over 48,000 patients [13]); extending study duration which increases costs and delays the adoption of trial findings [12] (clinical studies often double their original timelines in an attempt to meet their intended enrolment target [14]); and failure to identify participants who are eligible (e.g. up to 60% of potentially eligible patients may miss being identified [15], which disadvantages the potential participants as well as the study investigators, sponsors and end-users of the research). Inadequate retention of patients in clinical trials also reduces the available sample size for analysis and hence the statistical power to detect treatment effects [16]. Failure to achieve adequate recruitment and retention of participants in clinical trials therefore creates research waste [17] and may mean that policy-makers have to delay their decisions or base them on inferior evidence.

A survey of chief investigators of NIHR HTA trials and UK Clinical Research Collaboration (UKCRC)-registered clinical trials units (CTUs) identified that a diverse range of participant-retention strategies has been used in clinical trials, but for many of these, no research had been conducted to assess their effectiveness [18]. Another survey found that the top three priorities of UK CTU directors for trials methodological research included ‘Research into methods to boost recruitment in trials’(considered the highest priority) and ‘Methods to minimise attrition’ [19].

### Potential solutions: digital tools

To address inadequate patient recruitment and/or retention in studies, CTUs, funding agencies and study investigators are increasingly exploring the use of digital tools to identify, recruit and retain study participants. The range of digital tools that could assist with the recruitment and/or retention of study participants is wide. For example, a systematic review of the use of computers for patient recruitment into clinical trials found 79 different recruitment systems [20] and a survey of trials and CTUs identified a wide range of retention strategies [19]. Digital recruitment approaches may help potential participants to locate trials for which they are eligible, and/or help trial investigators or health professionals to identify potentially eligible participants. Specific digital tools for recruitment and/or retention of people in trials include (among others): automated telephone messaging [2, 21]; audio messages [22]; videos [2, 22]; radio and television advertisements [23, 24]; online advertisements [23, 25]; Internet websites and tools, including online surveys [21, 24, 26, 27]; social media [21, 23, 25, 27, 28]; ‘smartphone Apps’ [25]; computer pop-up reminders [29]; email messages [2, 21, 25]; text-messaging [2, 21, 23, 25] and automated eligibility screening of electronic health records, data warehouses or other patient data sources [15, 30–36]. The automated screening approaches may be subdivided according to the different algorithms employed for predicting patient eligibility, which include, among others, machine-learning approaches [34, 37] and case-based reasoning models [32, 33]. These digital tools may either be used alone or in various combinations, and may be combined with non-digital approaches. For example, a strategy to improve patient recruitment, enrolment, engagement and retention in a clinical trial of a weight-loss intervention for students included a smartphone App, television screens, email messaging, text-messaging, online advertisements and social media, as well as printed materials (flyers, coasters, pens, posters and postcards) [25]. In another example, an intervention to increase research participation of young female cancer survivors included Internet, email and social media components as well as newspaper advertisements that appeared both online and in print [38].

### Current evidence

The range of digital tools potentially available to assist with the recruitment and/or retention of patients in clinical and health trials is diverse, and a number of systematic reviews have investigated whether digital approaches for recruitment and/or retention are effective [2, 3, 20, 39–53] (summarised in Additional Table 1). However, it is difficult to get a comprehensive picture of the characteristics of the overall evidence base, because:

- These reviews have focussed on a single specific digital approach (e.g. Facebook [40, 51, 53] or social networking [39, 49]) or population (e.g. young people [40, 47, 49] or people with a specific condition [41, 48, 50]) or
- The reviews have been broad, not limited to digital approaches [2, 41, 43, 46–48] or
- The searches are now several years out of date [2, 3, 20, 39–52, 54]

Given the growth in the availability of digital tools in the last decade and the importance of effective and efficient recruitment and retention, we constructed a systematic map to investigate what evidence exists to support the use of these tools.

### Rationale for systematic mapping as an evidence-synthesis approach

The diversity, uniqueness, and, in some cases complexity, of strategies for improving patient recruitment and retention suggest that systematic mapping is an appropriate initial approach to the evidence synthesis, to identify and characterise the range of digital tools for improving patient recruitment and/or retention and characterise the studies that have evaluated these tools. Systematic mapping (NB also referred to in the literature as evidence mapping) begins in the same way as a systematic review, based on extensive searches for evidence and systematic screening to determine eligibility of the identified studies, but provides a descriptive output rather than an estimate of effects [55]. Systematic maps are helpful in summarising and describing a broad and/or heterogeneous evidence base in order to plan and prioritise focussed evaluative syntheses, e.g. using one or more subsequent systematic review(s) [55–62]. Evidence gaps can also be identified where little or no primary research has been conducted. We chose systematic mapping as the method for this project because no comprehensive overview of the evidence base relating to the use of digital tools for trial participant recruitment and/or retention currently exists.

### Systematic map research question

The question addressed by the systematic map is ‘what are the types and characteristics of the digital tools that have been evaluated for improving the recruitment and/or retention of people in RCTs?’

### Aims and objectives

The aim of this research is to systematically identify and describe the research studies that have evaluated the accuracy or effectiveness of digital tools for improving the recruitment and/or retention of people in RCTs, and to describe the characteristics of those digital tools that have been evaluated.

This research has two objectives:

- To develop a systematic map to identify and characterise the research studies that have evaluated digital tools for recruiting and retaining participants in clinical and health RCTs
- Based on the results of the map, to summarise any key evidence gaps and areas where a more detailed, focused, evidence synthesis of effectiveness could be worthwhile; and to provide recommendations for further primary research as necessary

## Methods

### Project management and protocol

This research was part of a broader project ‘User-focussed research to identify the benefits of innovative digital recruitment and retention tools for more efficient conduct of randomised trials’. The project had three parts: Phase 1: Scoping searches to identify digital tools for patient recruitment and retention. Phase 2: A survey of the UKCRC-registered CTUs on what digital tools they currently use, and interviews with stakeholders to identify which digital tools for trial recruitment and retention are currently being used in practice and the performance characteristics required for digital tools to be judged useful [63]. This informed the development of logic models to capture the functions of different types of digital tools for recruitment and retention and the theory behind their mechanism of action in a generic way, without focussing on any specific digital tool, product or service. Phase 3: Systematic mapping to identify and describe the characteristics of research studies which have evaluated the effectiveness of digital tools for patient recruitment and/or retention. The research we are reporting here is specifically on the systematic mapping work (Phase 3).

The systematic map methods were based on a protocol (Appendix 1; see Additional file 4) which was peer-reviewed by the full project team and an Advisory Board prior to the commencement of the work. The Advisory Board included representatives of the NIHR research infrastructure (i.e. Research Design Service and Clinical Research Network), clinical trials units, a patient and public involvement representative, and academic members of a National Health Service (NHS) Foundation Trust (see the ‘Acknowledgements’ section). The systematic mapping was conducted by a team experienced in evidence synthesis (the authors of this paper) who met monthly with, and received feedback from, the full project team. The work was conducted in accordance with those steps of the Preferred Reporting Items for Systematic Reviews and Meta-Analyses (PRISMA) Checklist [64] that are applicable to systematic mapping (see Additional Table 2).

### Searches

A comprehensive search for relevant studies was undertaken by an experienced health information specialist in the following bibliographical databases:

- Ovid MEDLINE (including MEDLINE Epub Ahead of Print, MEDLINE In-Process and Other Non-Indexed Citations and MEDLINE Daily; 1990 to July 2018)
- Ovid Embase (1990 to 17 July 2018)
- Inspec (Institute of Engineering and Technology) (1990 to 18 July 2018)
- Web of Science (a cross-database search including the BIOSIS Citation Index, BIOSIS Previews, Current Contents Connect, Data Citation Index, Derwent Innovations Index, Inspec and the KCI Korean Journal Database; 2000 to 18 July 2018)

The search strategies for MEDLINE, Embase, Web of Science and Inspec searches are shown in Appendix 2 (see Additional file 5). Where possible, the databases were searched from 1990 onwards, as specified in the protocol (Appendix 1; see Additional file 4). As noted below (see the ‘Eligibility criteria’ section), a further date limit was subsequently applied during eligibility screening.

All searches were conducted in the English language.

### Eligibility screening process

Titles and abstracts of references identified by the search were screened against the eligibility criteria (stated below) independently by two reviewers. In cases of disagreement a third reviewer was consulted to reach a consensus. Full-text articles were retrieved for those titles and abstracts which met the eligibility criteria or which had unclear relevance, and for references that did not have an abstract or summary (e.g. Internet pages and reports). Full-text articles were screened by one reviewer and checked by a second, and a third reviewer was consulted in cases of disagreement. The screening process is reported according to the Preferred Reporting Items for Systematic Reviews and Meta-Analyses (PRISMA) guideline [65] (see the ‘Results’ section).

### Eligibility criteria

References were screened using an eligibility worksheet, which was pilot-tested on 29 studies identified by our scoping searches [16, 18, 28, 35, 61–85] to ensure consistency of the review team’s interpretation. The final eligibility criteria (Appendix 3; see Additional file 6) were then applied to all the identified references. The same eligibility criteria were applied for screening titles, abstracts and full-text articles.

#### Populations

##### Inclusion criteria

Studies featuring one or more of the following population groups were eligible:

- Health professionals (e.g. physicians, nurses, therapists)
- Researchers and study administration staff (e.g. study investigators, research managers)
- Patients, their carers or the general public
- Healthy volunteers

##### Exclusion criteria

- People from disciplines outside health, medical or clinical research
- Mixed populations in which not all participants met the inclusion criteria and outcomes were not separately reported for those who met the inclusion criteria

#### Interventions

Studies were eligible for inclusion if they evaluated one or more *digital approach* to recruit and/or encourage retention of participants into clinical and health studies. To be classed as a *digital approach*, the method of participant recruitment or retention had to include one or more *digital tools*. A *digital tool* was defined broadly in this project as: an Internet, software or social media application, computer, electronic tablet or virtual assistant/gadget, to support recruitment and/or retention of participants in RCTs. According to the logic models we developed (unpublished data, available from the authors on request), digital approaches for the recruitment of participants could include methods that: raise awareness of trials; help eligible people to locate trials; identify eligible patients from databases or during clinical consultations; and/or check a person’s eligibility for a trial. Digital approaches to support retention of participants in trials could include, among others, methods that provide trial information and/or reminders for trial participants to provide data or to attend visits or tests.

Studies of multi-component digital approaches (e.g. which included digital in addition to non-digital tools) were eligible for inclusion.

#### Comparators

##### Inclusion criteria

Only studies that included a comparator were eligible. The comparator could include:

- Standard practice for participant recruitment or retention (for the given study sponsor or institution conducting the study)
- Non-digital approaches (e.g. approaches that comprise paper-based or manual tools)
- Recruitment or retention approaches comprising digital tools other than those included in the intervention
- Digital approaches comprising ‘bundles’ of tools (i.e. where the comparator included more than one digital and/or non-digital tool)

##### Exclusion criteria

Studies were excluded if the configuration of the comparator and intervention was such that effects of digital tools could not be separated from the effects of non-digital tools (e.g. if the intervention and comparator both contained digital and non-digital strategies but the digital strategies were identical and, therefore, their effects would ‘cancel out’).

#### Outcomes

Studies that reported one or more of the following outcome measures were eligible for inclusion:

- Recruitment rate (e.g. the proportion of the intended number of participants enrolled in the study)
- Quantitative assessment of recruitment accuracy (e.g. the proportion of participants included in a study accurately meeting study inclusion criteria, as assessed by sensitivity, specificity and/or area under the curve estimates [31, 36])
- Qualitative assessment of recruitment accuracy (e.g. similarity of the characteristics of the recruited participants against the study eligibility criteria)
- Participant retention in a study (e.g. the proportion of recruited participants who remained in the study at the end)

#### Study designs

Two types of study design are relevant to the systematic map:

- The design of the RCT into which participants were recruited and retained. For clarity we refer to this as the *host trial*
- The design of the research that investigated the accuracy or effectiveness of the digital approaches for recruitment and/or retention in the host trial. We refer to this as the *primary evaluation study*. For clarity, throughout the rest of this paper we refer to ‘trial’ when referring to the host trial and ‘study’ when referring to the primary evaluation study
- To be eligible for inclusion, the host trial had to be an RCT. The primary evaluation study could be of any design (e.g. RCT, quasi-experimental study or observational study), provided that relevant outcomes (as specified above) were reported for the intervention (i.e. the digital recruitment or retention approach of interest) and at least one comparator. The primary evaluation studies could conform to the broad definition of a ‘study within a trial’ (SWAT), which is ‘a self-contained research study that has been embedded within a host trial with the aim of evaluating or exploring alternative ways of delivering or organising a particular trial process’ [66]. However, in order to fully capture the range of evaluative research studies that have been conducted on RCT recruitment and retention, our inclusion criteria are wider than those that would define a SWAT. For example, we permitted retrospective studies to be included; and we did not require evidence that studies were based on a formal protocol, as is recommended for SWATs [66].

Studies were limited to those published during the last 10 years (i.e. those published from the start of 2008 to the date of searches in 2018), to ensure that digital tools included in the studies are likely to be reflective of the tools available for use in current practice. A cut-off date for study eligibility was not specified in the original protocol when searches were conducted but was subsequently agreed in consultation with the project Advisory Board, as specified in a protocol amendment (Appendix 1; see Additional file 4).

### Coding of eligible studies and development of the systematic map

All studies meeting the criteria for inclusion in the map were classified by systematically applying pre-specified keywords to each study. The purpose of the keyword list was to capture the characteristics of the evidence base in a flexible manner such that the key attributes of studies of interest to end-users could be clearly summarised in the map, and specific combinations of characteristics of interest explored. The map, and hence the keywords, did not seek to critically appraise, synthesise or evaluate the results of the studies, for which systematic review and meta-analysis would be more appropriate evidence-synthesis methods. The draft keyword list and coding process were initially pilot-tested on eight studies that were identified in scoping searches (reported in five papers [21, 31, 67–69]), to ensure that the map would consistently capture relevant information. The draft keyword list was refined based on consultation with the full project team and the project Advisory Board (which we consider representative of most stakeholders likely to consult the map), to give a final version (Appendix 4; see Additional file 7).

### Data extraction strategy

Each study was coded by one reviewer and a random sample of 20% of the studies was checked by a second reviewer. The coding decisions for each study were recorded in a Microsoft Excel spreadsheet template, which contained the list of keywords (rows) and a list of the included studies (columns) to generate a data matrix that would form the systematic map database. In cases where checking identified that refinements to coding would be appropriate these were agreed by the review team and applied to all the included studies to minimise the risk of introducing bias. Once the coding of all the included studies had been completed, Excel chart and pivot table functions were applied to the data matrix to produce a descriptive map of the key characteristics of the evidence.

## Results

### Searching, study selection and map coding

**Fig. 1:**
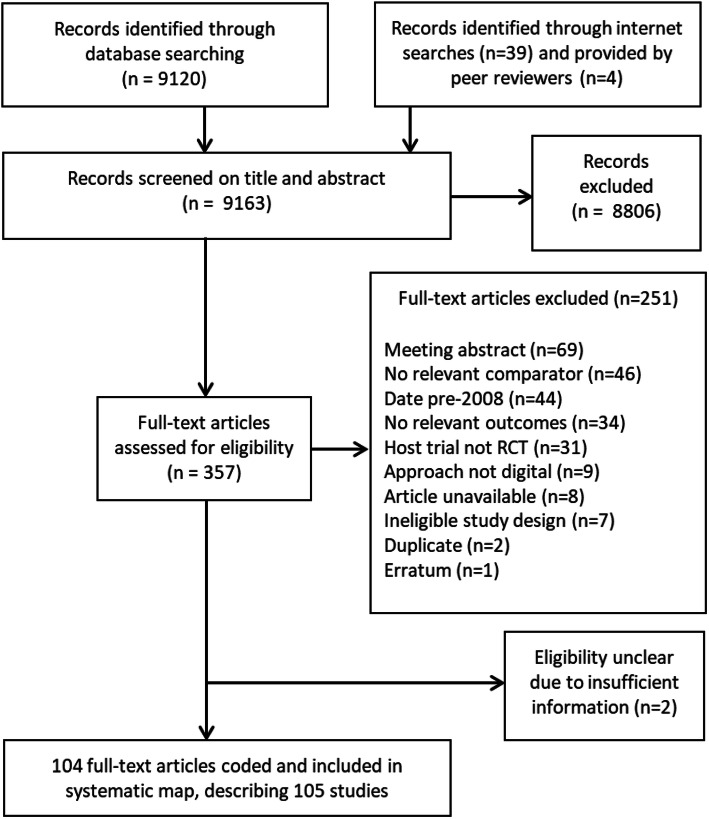
Eligibility screening flow diagram

A flow diagram summarising the eligibility screening process is shown in Fig. 1. After removing duplicates, searches identified 9163 unique references published from January 2008 to mid-July 2018. The majority of these (*n* = 8806) were excluded because their title and/or abstract did not meet the eligibility criteria, leaving 357 articles for full-text screening. A further 251 articles were excluded at full-text screening (Fig. 1) (a full list of the 251 excluded studies and the reasons for exclusion is provided in Additional Table 3). The remaining 104 articles, reporting 105 unique studies, passed the full-text assessment and were included in the systematic map [28, 32–36, 70–167].

#### Reviewer eligibility screening agreement

Two reviewers independently conducted all eligibility screening steps and we considered the overall rates of reviewer agreement to be good, given the topic complexity and the heterogeneity of reporting styles in the articles. The overall agreement rate for title and abstract screening was 97%, which primarily reflects very good agreement on the large number of decisions to exclude records. The overall agreement rate at the full-text screening step was 91%, with 9% of the full-text articles (*n* = 31) requiring discussion before the eligibility decision was finalised.

#### Reviewer map-coding agreement

The 20% check of coded studies by a second reviewer showed good overall agreement between reviewers in how they had applied the keywords. Generally, only minor changes were made to coding studies after discussion between the reviewers. The main issue identified by the check was that the ‘recruitment reach’ outcome keyword had not been applied consistently by all the reviewers. As a result of this, we created a new, clearer definition for this outcome (Appendix 4; see Additional file 7) and updated all the studies in the map to ensure that where studies reported ‘reach’ this was coded consistently.

### Systematic map results

The full systematic map database is provided in Appendix 5 (see Additional file 8).

### Characteristics of the studies

**Fig. 2:**
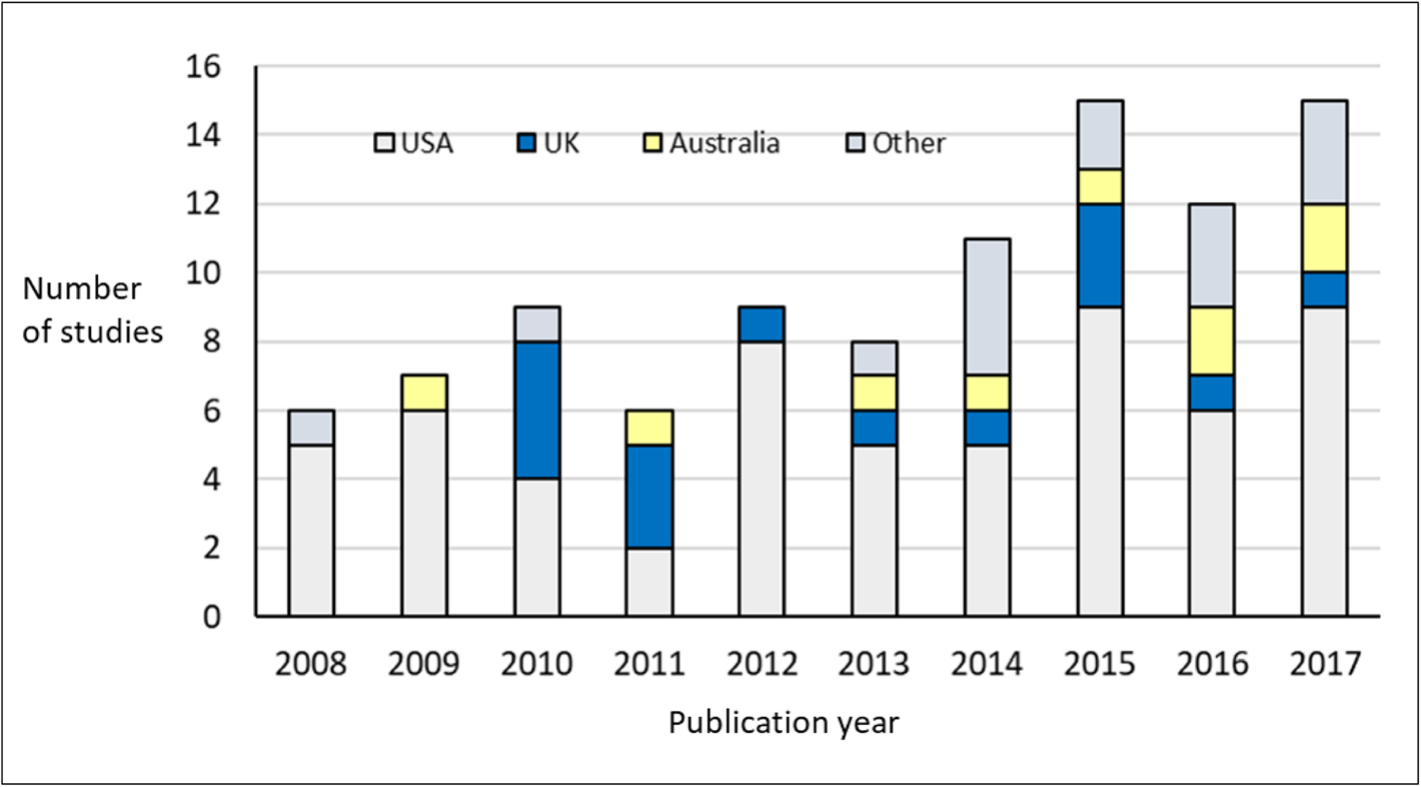
Geographical and temporal distribution of the studies. Data from 2018 are excluded as they are not available for the whole year

The 105 identified studies of digital recruitment and retention approaches have nearly all been conducted in single countries (100/105; 95%), with five multi-national studies identified (5%). The 100 single-country studies were conducted most often in the USA (61%), UK (17%), Australia (9%), Germany (4%) and Canada (3%), with a slight increase in the diversity of countries involved over time (Fig. 2).

**Fig. 3:**
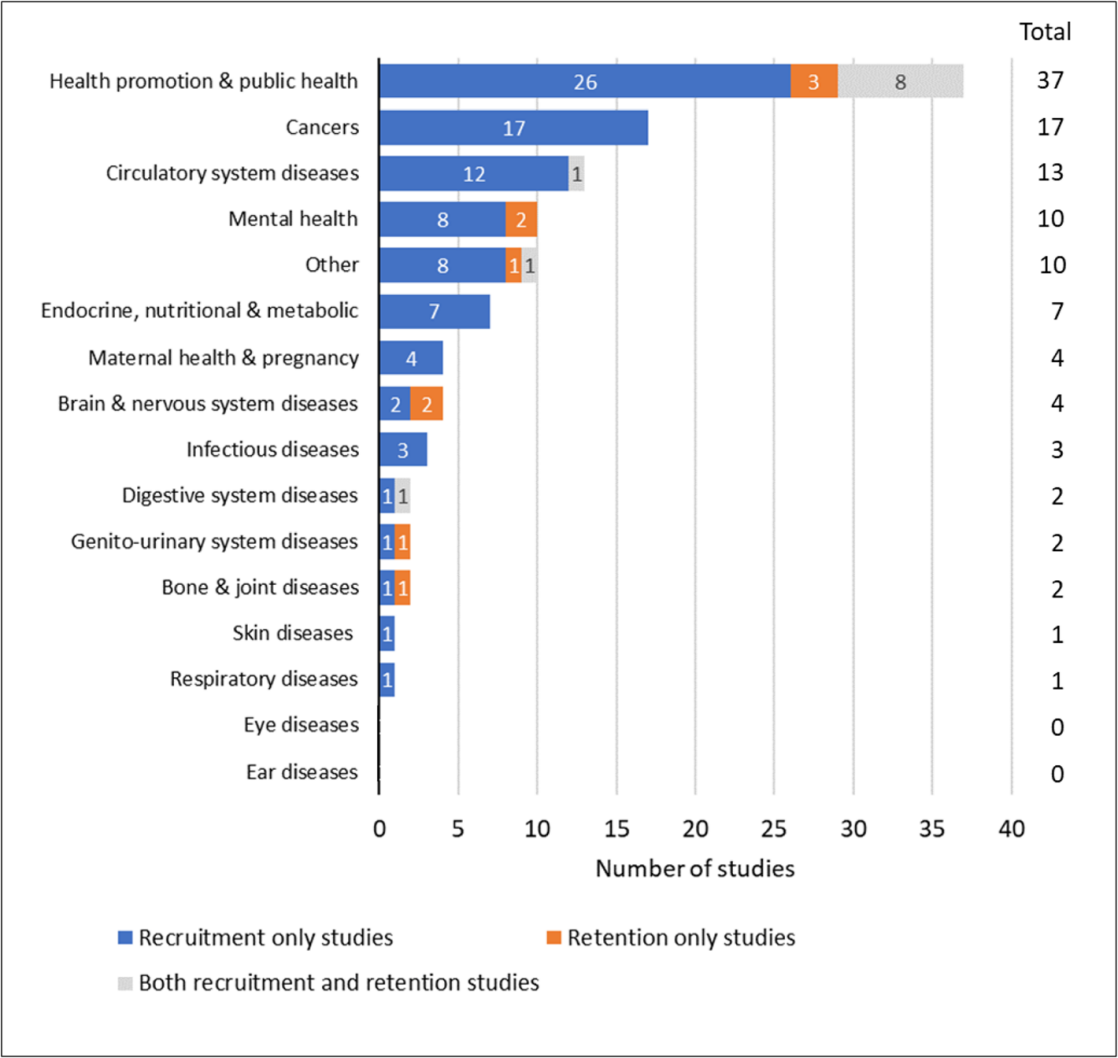
Health topics assessed in studies of digital tools for recruitment and/or retention

The health areas studied most often were health promotion and public health (37/105; 35%), cancers (17/105; 16%), circulatory system disorders (13/105; 12%), mental health (10/105; 10%) and endocrine and metabolic disorders (7/105; 7%) (Fig. 3). Note that the total number of studies in Fig. 3 is more than 105, since several studies covered more than one health condition.

**Fig. 4:**
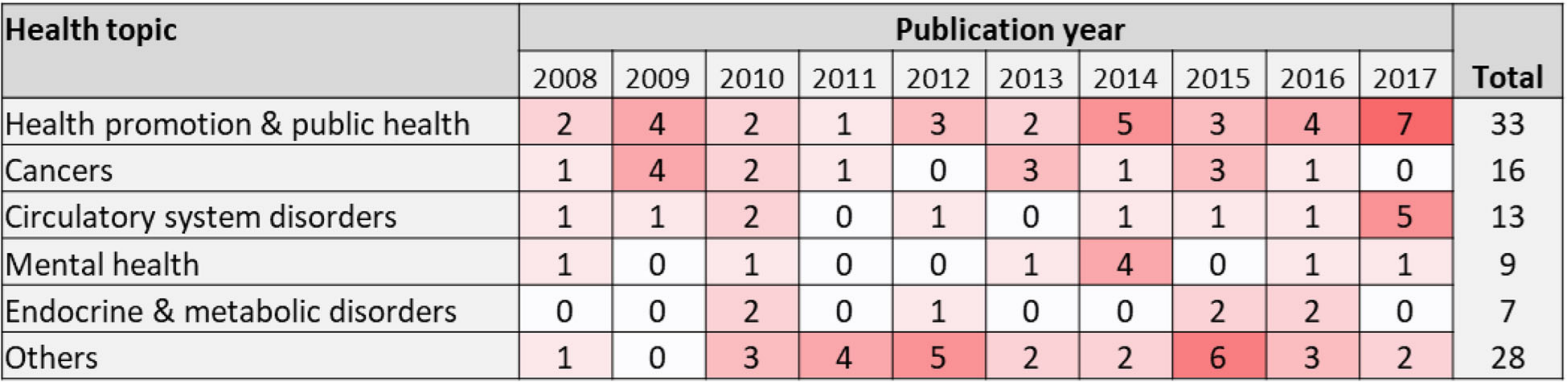
Distribution of the most frequently-studied health topics in relation to publication year. Data from 2018 are excluded as they are not available for the whole year

Studies on health promotion and public health and on circulatory system disorders have recently increased, but those on cancers, mental health and endocrine and metabolic disorders have remained relatively infrequent (Fig. 4).

The systematic map (Appendix 5; see Additional file 8) shows that the most common specific health topics that the 37 studies on health promotion and public health investigated were smoking cessation or tobacco control (11 studies) and sexual health promotion (seven studies).

**Fig. 5:**
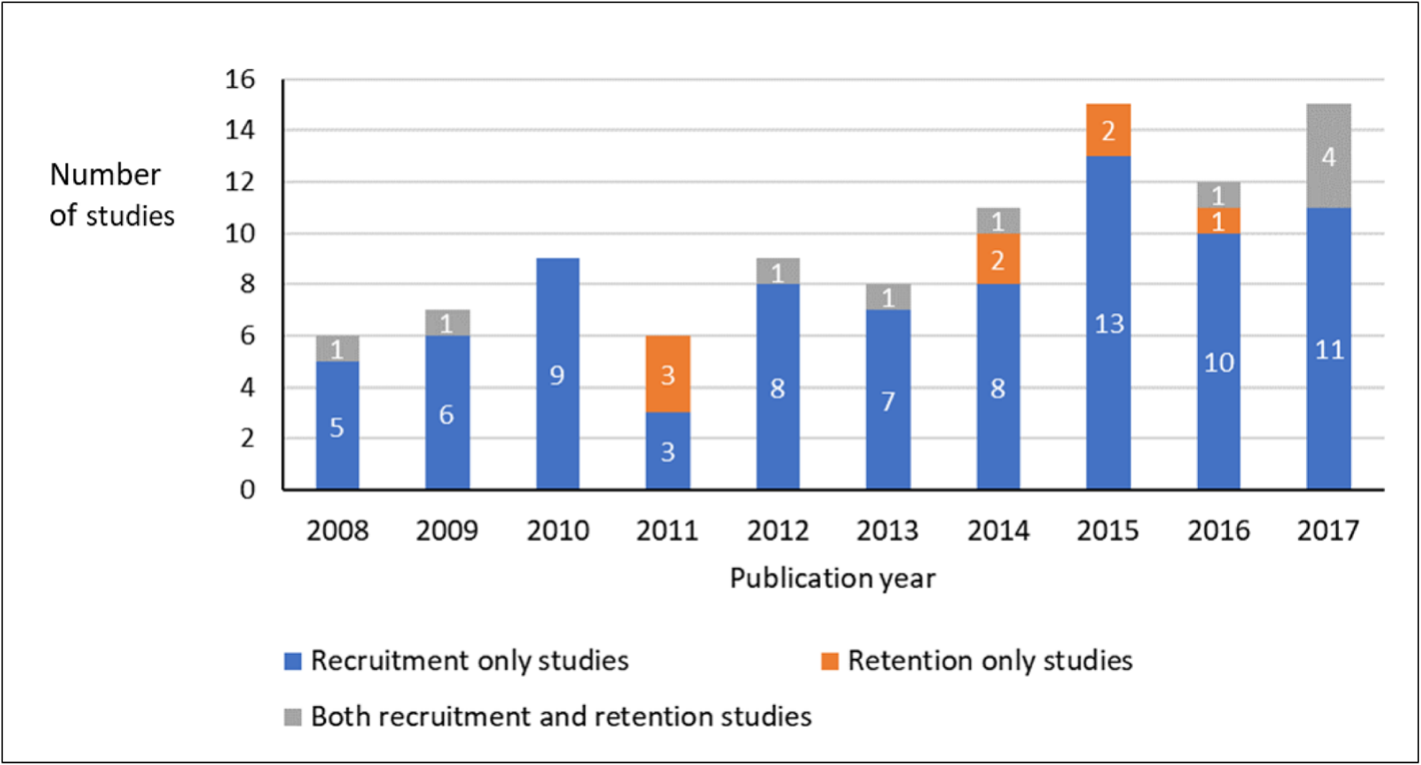
Distribution of studies of digital tools for recruitment and/or retention in relation to publication year. Data from 2018 are excluded as they are not available for the whole year

Most of the studies (96/105; 91%) have investigated digital approaches for recruitment, with fewer (20/105; 19%) investigating digital approaches for retention, and 11/105 (10%) investigating both recruitment and retention (85/105 studies (81%) were on recruitment only and 9/105 studies (9%) were on retention only). The greater frequency of studies on recruitment than retention has been consistent across the different health topics investigated (Fig. 3). The number of studies published each year has approximately doubled during the past decade; this primarily reflects an increase in studies of recruitment, with only a slight increase evident in the number of studies that assessed retention (Fig. 5). The study designs have been primarily observational and/or retrospective (90/105; 86%), with 11 randomised experiments (10%) and five non-randomised experimental studies (5%).

Among the 11 studies that evaluated digital tools for both recruitment and retention, five (45%) used different digital tools for recruitment and for retention [79, 80, 97, 104, 162]. Four studies employed at least one digital tool that was common to both recruitment and retention, i.e. email [73, 106, 126, 139] and Facebook [126], but in each case the tool was tailored to function differently for recruitment (it included adverts) and retention (it included reminders or facilitated engagement). The remaining two studies assessed the effect of their digital recruitment approach on retention, effectively assuming that the recruitment tools also acted as retention tools [99, 150].

### Characteristics of the digital approaches

The systematic map (Appendix 5; see Additional file 8) shows that the digital interventions for recruitment and/or retention tested in the studies were a single digital approach in 41% of the studies (43/105), digital approaches combined with non-digital approaches in 50% (53/105), and multiple combined digital approaches in 10% (10/105) (totals exceed 100% as one study included different approaches for recruitment and retention).

Nearly half of the studies (46/105; 44%) did not explicitly state that they included a comparator, but they presented outcomes in such a way that a comparator was discernible (e.g. outcomes were reported separately for different tools). Sixty-four studies defined their comparator(s) (the total number of studies exceeds 105 as some studies included more than one comparison). Of these, 13% of the studies (8/64) employed a comparator that was a single digital approach, whilst 48% (31/64) employed a comparator that was a single non-digital approach. In 16% of the studies (10/64) the comparator comprised a mixture of digital and non-digital approaches whilst in a further 16% the comparator was a mixture of different non-digital approaches. Only one study (1%) employed a combination of multiple different digital approaches as the comparator. The remaining 4/64 studies (6%) gave unclear information, classified as “other” in the map.

#### Recruitment approaches and tools

**Fig. 6:**
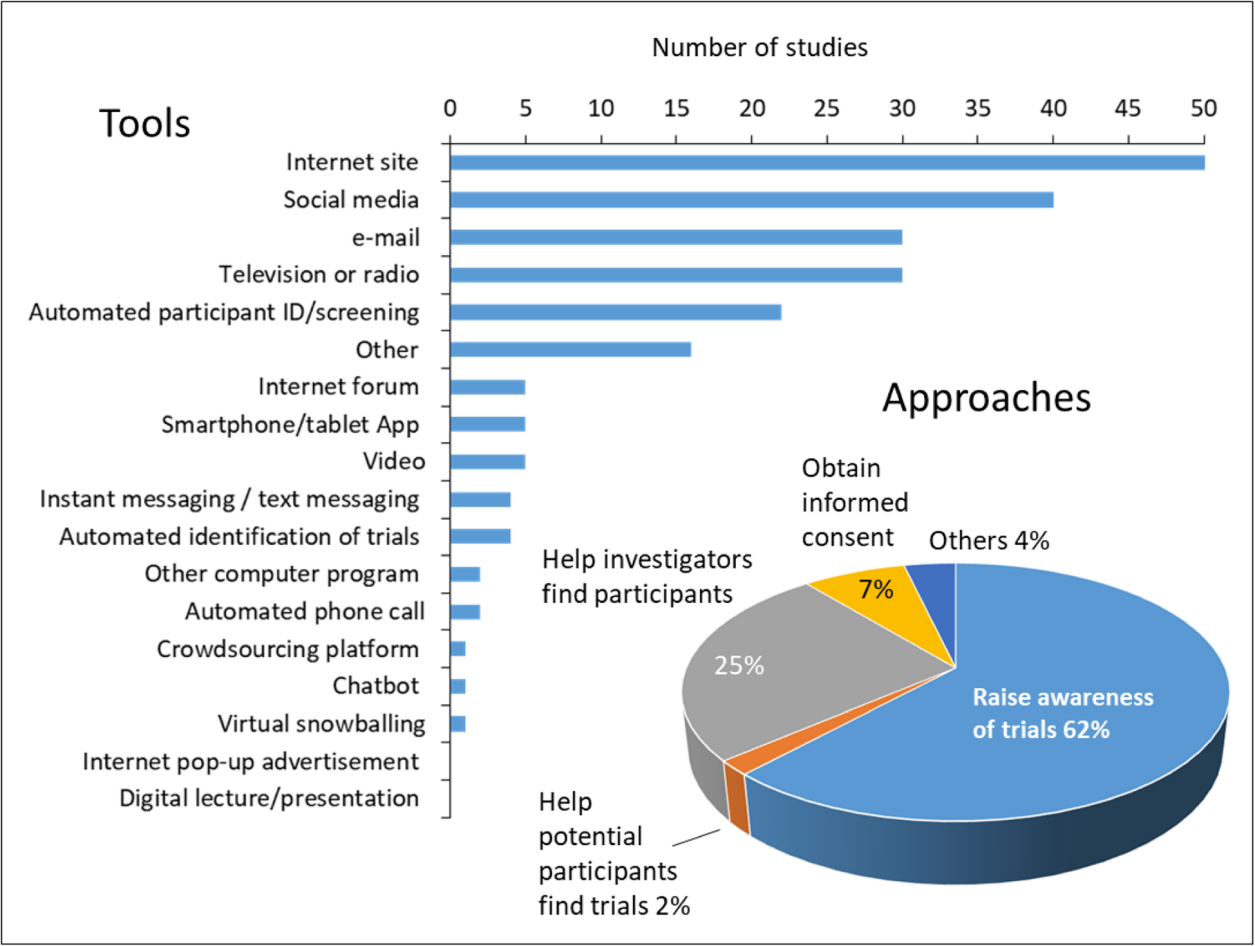
Overview of digital approaches and tools for recruitment

An overview of the digital approaches and tools evaluated for recruitment is shown in Fig. 6. Several studies reported more than one approach, so the number of approaches (*n* = 110) exceeds the number of recruitment studies (*n* = 96).

The most commonly evaluated digital recruitment approaches aimed to raise awareness of trials among potential trial participants (68/110; 62%) and to help investigators and clinicians to identify eligible participants for their trials (28/110; 25%). A further 7% of the recruitment approaches (8/110) aimed to obtain informed consent, while 2% (2/110) provided search aids for people to identify specific trials that they could join.

The specific digital recruitment tools evaluated in the 96 studies that assessed recruitment are summarised in Table 1. The most frequently investigated tools in the last decade have been Internet sites (51/96; 53% of studies), social media (40/96; 42%), television or radio (30/96; 31%), email (30/96; 31%), and automated approaches for identifying potential participants (22/96; 23%). Note that some studies employed more than one recruitment approach, so the sum of these percentages exceeds 100.

**Table 1:** Types of digital tool for recruitment evaluated in the studies

Digital tool	Number of studies	% of recruitment studies (*n* = 96)	% of all studies (*N* = 105)
Internet site	51	53	49
Social media	40	42	38
email	30	31	29
Television or radio	30	31	29
Automated screening to identify potential participants	22	23	21
Internet forum	5	5	5
Smartphone or tablet App	5	5	5
Video	5	5	5
Automated identification of trials for which people are potentially eligible	4	4	4
Instant-messaging or text-messaging	4	4	4
Automated telephone call	2	2	2
Other computer programme	2	2	2
Chatbot	1	1	1
Crowd-sourcing platform	1	1	1
Virtual snowballing	1	1	1
Smartphone or tablet other use	1	1	1
Internet pop-up advertisements	0	0	0
Digital lecture/presentation (e.g. PowerPoint)	0	0	0
Others	16	17	15

Note: studies could use more than one tool type, so the total number of studies and the percentages do not summate to *N* = 96 and 100%, respectively.

**Fig. 7:**
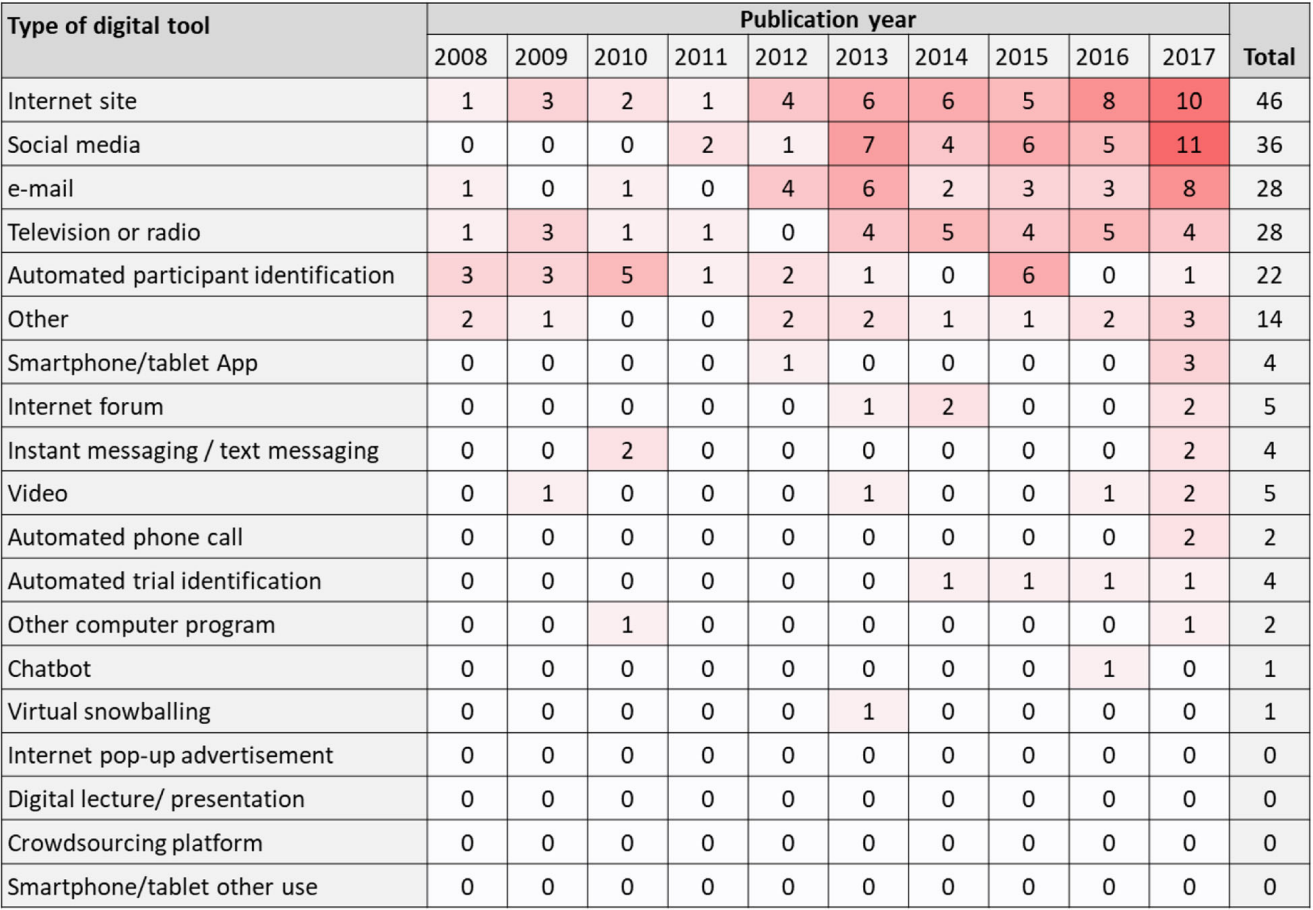
Types of digital recruitment tools studied in relation to publication year. Data from 2018 are excluded as they are not available for the whole year

The map shows that there has been a recent increase in the number of studies evaluating Internet sites and social media for recruitment (Fig. 7).

Overall, the specific recruitment approaches employed, and their combinations, have been diverse, meaning that each study has effectively assessed a unique recruitment approach. These have included, for example: online advertisements using banners [79, 80, 106] or Google Adwords [81, 105]; advertisements in cinemas [86]; Internet press releases [82]; electronic newsletters [131]; podcasts and webinars [75]; and online community notice boards [128]. Where the recruitment approach included both digital and non-digital tools, the most frequent non-digital components were flyers (32/96 studies; 33%) or mail outs (27/96 studies; 28%).

#### Retention approaches and tools

**Fig. 8:**
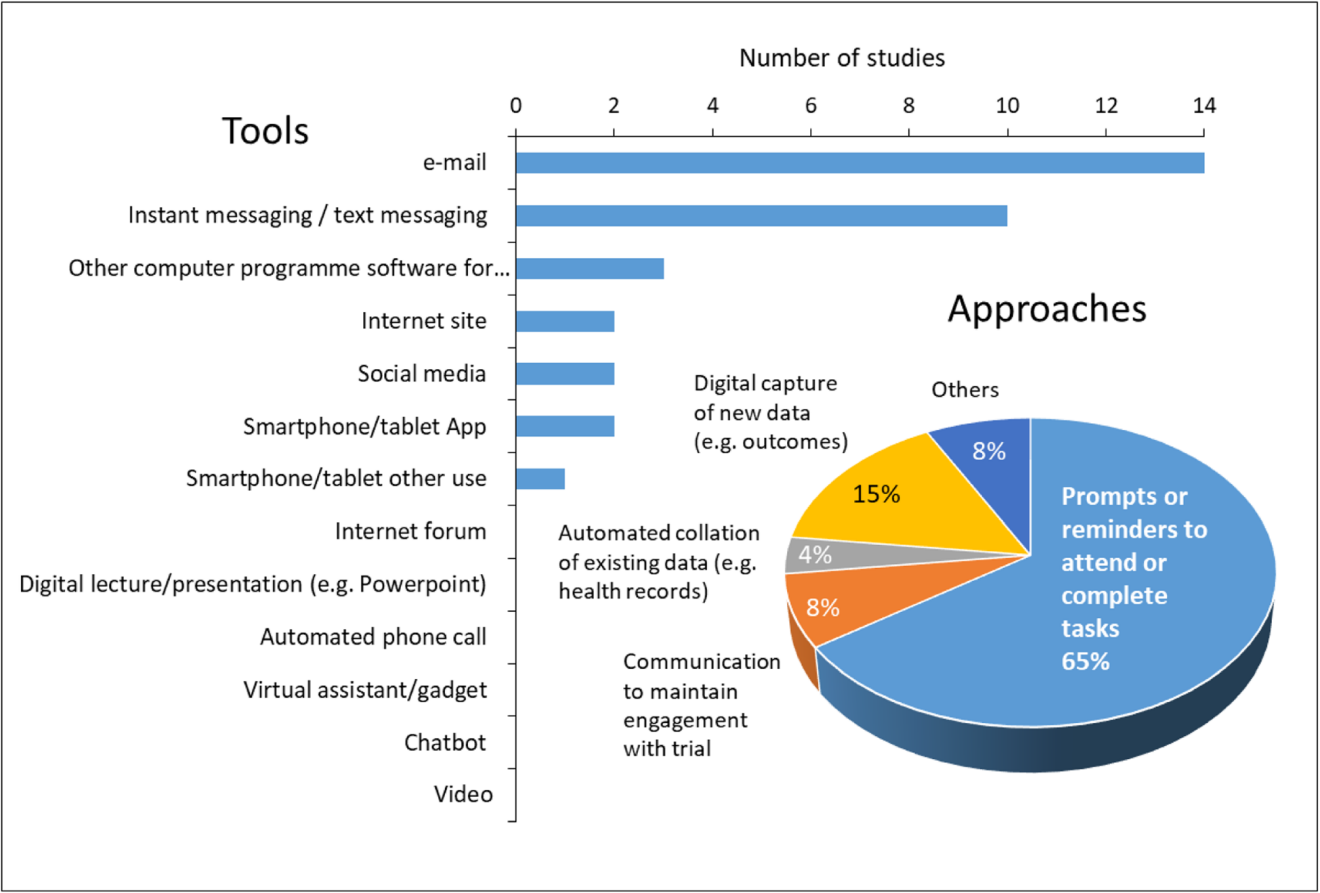
Overview of digital approaches and tools for retention

An overview of the digital approaches and tools evaluated for retention is shown in Fig. 8.

Several studies reported more than one approach, so the number of approaches (*n* = 26) exceeds the number of retention studies (*n* = 20). The digital retention approaches in most cases (17/26; 65%) provided prompts or reminders for trial participants to attend study appointments or to complete outcome assessments. The remaining approaches mainly involved the digital collection of new or existing data to improve the completeness of data availability (5/26; 19%) or communication approaches to maintain participants’ engagement with the trial (2/26; 8%).

The specific digital retention tools evaluated are summarised in Table 2.

**Table 2:** Types of digital tools for retention evaluated in the studies

Digital tools	Number of studies	% of retention studies (*n* = 20)	% of all studies (*N* = 105)
email	14	70	13
Instant-messaging or text-messaging	10	50	10
Other computer programme	3	15	3
Internet site	2	10	2
Smartphone or tablet App	2	10	2
Social media	2	10	2
Smartphone or tablet other use	1	5	1
Automated telephone call	0	0	0
Chatbot	0	0	0
Digital lecture/presentation (e.g. PowerPoint)	0	0	0
Internet forum	0	0	0
Video	0	0	0
Virtual assistant/gadget	0	0	0

Note. Studies could use more than one tool type, so the total number of studies and the percentages do not summate to *N* = 20 and 100%, respectively

**Fig. 9:**
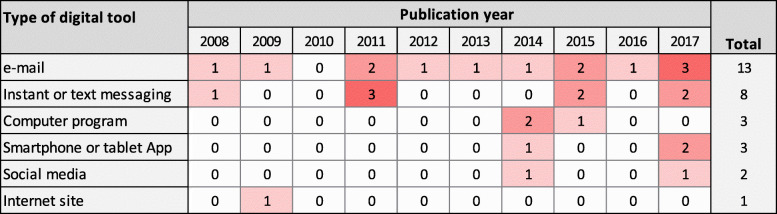
Types of digital retention tools studied in relation to publication year. Data for 2018 are excluded as they are not available for the whole year

Among the 20 studies that investigated retention, the most frequently investigated digital retention tools were email (14/20 studies; 70%) and/or instant-messaging or text-messaging (10 studies; 50%). The distribution of research on retention is more sparse than that for recruitment, without any clear trends through time (Fig. 9). Although social media has been widely used for participant recruitment, only two of the studies identified in the map investigated the potential of social media for improving retention. No studies had investigated whether chatbot or video-based approaches could assist the retention of participants in trials.

### Types of recruitment and retention outcomes studied

**Fig. 10:**
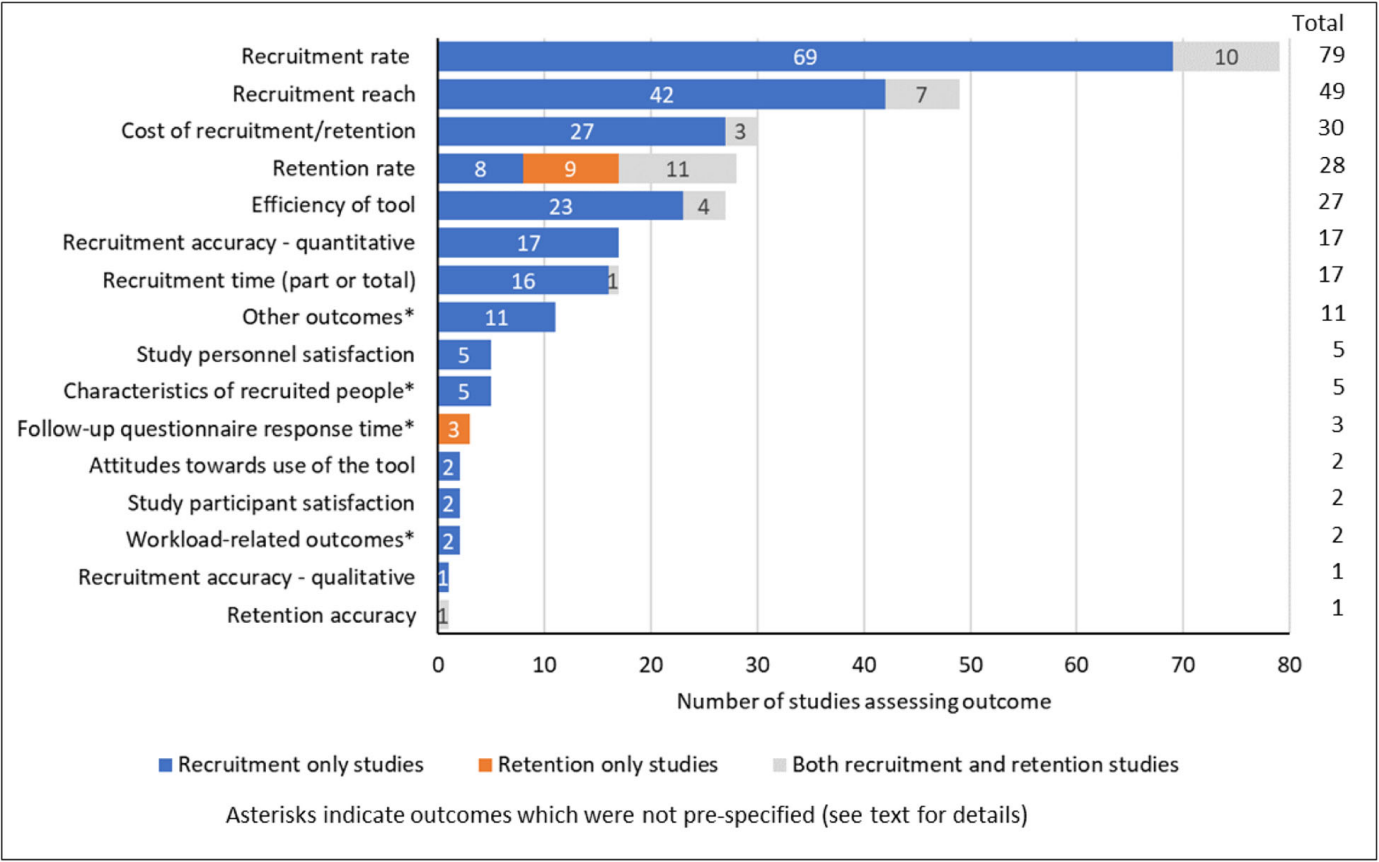
Outcomes assessed in studies of digital tools for recruitment and/or retention

The types of recruitment and retention outcomes assessed in the studies are summarised in Fig. 10.

The most frequently reported recruitment outcome in the map (Appendix 5; see Additional file 8) was the recruitment rate, reported in 79 of the 96 recruitment studies (82%). Seventeen of the recruitment studies (18%) reported the quantitative accuracy of recruitment compared against a reference standard, and the time to complete one or more parts of the recruitment process was also reported in 18%. The costs of the digital approaches were reported in 30 of the 105 studies in the map (29%), but the focus was on recruitment, with only three of these studies mentioning any costs in relation to retention. Twenty-eight of the 105 studies in the map (27%) reported the retention rate, but eight of these had investigated digital tools for recruitment rather than for retention. Few studies assessed the satisfaction with digital tools perceived by study personnel (5/105 studies; 5%) or study participants (2/105 studies; 2%), or people’s attitudes towards using the tools (also 2%).

In addition to the pre-specified keywords, the map coding (Appendix 4; see Additional file 7) allowed us to record ‘other’ outcomes if they appeared relevant. We recorded other outcomes for 21 studies. The textual descriptions for these outcomes in the map spreadsheet (Appendix 5; see Additional file 8) indicate that five studies assessed the descriptive characteristics of recruited people, three studies assessed follow-up questionnaire response times, and two studies reported outcomes relating to study workloads (Fig. 10). The remaining 11 studies reported other outcomes that were unique (i.e. specific to each study).

### Application of digital tools in specific populations

Sixteen studies (15% of all those included in the map) evaluated the use of digital tools for trial recruitment or retention with a focus on individuals from minority or under-served populations (as defined by the study authors). Minority or under-served populations included in studies of recruitment were: Black, African-American and Hispanic people [77, 89, 110, 113, 135, 146, 149, 154]; Maori or Pacific Island populations [162]; Turkish migrants with depressive symptoms [159]; men who have sex with men [107]; transgender women [107]; other people at risk of HIV infection [137]; people with low computer and health literacy [76]; Gulf War veterans [96]; infertile couples [160]; and low-income gay, lesbian and bisexual smokers [77]. One study focussed specifically on the retention of a minority/under-served population: of low-income, vulnerable, smokers with mental illness in psychiatric units [114].

Overall, the most commonly evaluated digital tools in studies on minority or under-served populations were Internet sites for recruitment (reported in 13/16 studies; 81%), television or radio for recruitment, social media for recruitment, and email for recruitment (each reported in 9/16 studies; 56%) (the percentages do not summate to 100 as some studies evaluated more than one tool).

The health-topic area with the largest number of studies that included a focus on minority or under-served populations was health promotion and public health (8/37 studies; 22%) (Table 3). For most health-topic areas the total number of studies was small, but it is notable that only 1 of 17 studies on cancers, and only 2 of 13 studies on circulatory system disorders included a focus on minority or under-served populations.

**Table 3:** Health topics where digital tools studies have focussed on minority or under-served populations

Health-topic area where digital tools have been evaluated	Number (%) of studies	
	Total studies	Studies on minority or under-served populations
Health promotion and public health	37	8 (22)
Cancers	17	1 (6)
Endocrine, nutritional and metabolic disorders	17	0
Circulatory system diseases	13	2 (15)
Mental health	10	2 (20)
Other	10	2 (20)
Maternal health and pregnancy	4	1 (25)
Brain and nervous system diseases	4	0
Infectious diseases	3	1 (33)
Digestive system diseases	2	0
Bone and joint diseases	2	0
Genito-urinary system diseases	2	0
Respiratory diseases	1	0
Skin diseases	1	0

Note: Percentages summate to more than 100 as some studies included more than one health-topic area

We did not code participant age in the map keywords. However, inspection of the study publications after the map was completed shows that none of the studies on digital tools for retention included populations of older people or children. In contrast, four of the studies of digital recruitment tools were specifically on populations of older people [125, 143, 152, 155] (although the definition of ‘older’ varied, e.g. range 50 to 75 years [125], mean 69 years [155], median 70 years [152] or range 70 to 93.9 years [143]), whilst three studies were on children [34, 130, 141].

## Discussion

The work that we have reported here is the first systematic map to describe the characteristics of studies that have evaluated digital tools for improving participant recruitment and retention in clinical trials. We have employed systematic mapping as our aim was to provide information on the scope, nature and content of the empirical research that has been carried out on digital tools for trial recruitment and retention, rather than to answer a specific policy question. The systematic map database, which is provided as supporting material alongside this article (Appendix 5; see Additional file 8) may be examined in detail by anyone interested in exploring the characteristics of the studies and digital tools further, and may be updated, or expanded in scope, to suit emerging research needs.

### Links between recruitment, retention and other digital aspects of trial management

Tools that optimise both recruitment and retention would be desirable, since failure to retain adequate participants in a trial would negate the benefits of achieving good recruitment. In a survey of trial investigators and UK CTUs, the need for strategies to improve retention in trials was identified as being a key research priority [168], whilst the PRioRiTy studies have identified the use of technology in the trial recruitment and retention processes as being within the top 10 research priorities for the future [169]. However, the map shows that most of the studies (81%) have evaluated digital tools only for recruitment. Where studies did utilise digital tools both for recruitment and retention the tools that they used for recruitment and retention were different [73, 79, 80, 97, 104, 106, 126, 139, 162]. This is not surprising, since the approaches for recruitment were primarily to raise awareness whilst the approaches for retention were primarily to provide reminders or to collect data (see Figs. 6 and 8).

We did not specifically seek studies of digital approaches for managing other components of trial management beyond recruitment and retention and so our map keywords do not cover these aspects. However, according to the study publications, several of the studies in the map that evaluated digital tools for both recruitment and retention had included further digital methods for other purposes. These digital methods covered: online patient verification [79, 80]; online randomisation [73, 104]; allocation concealment [73]; automated data collection [73, 104]; information dissemination from investigators to participants [104]; email and website options for participants to contact investigators [139]; data monitoring and tracing [97]; and data-quality checking [73]. A key question is whether the efficiency of clinical trials could be improved by developing a small set of compatible digital tools that would cover all of these aspects of trial management, rather than requiring trial investigators to select separate digital tools to cover recruitment, retention and other aspects of trial management. Further research would be helpful to understand whether (and, if so, how) the different digital tools interact with one another, to ensure that the most efficient combinations of tools can be selected by trial investigators. However, as there are many different possible permutations and combinations of different tools that could be employed in clinical trials for different populations and health conditions, it may be necessary to identify for further research a core set of tools that appear to show the most promise in improving recruitment and retention efficiency.

### Use of digital tools in relation to the population and health topic

The appropriateness and efficiency of digital tools for recruitment and retention is likely to be vary across populations and health conditions. The 17 studies on cancers in the map focussed solely on recruitment, with none having investigated digital tools for retention (Fig. 3). A possible explanation could be that retention tools are not required in studies where the primary outcome is survival, or that for other outcomes cancer patients in general are highly motivated, by their need for life-prolonging therapy, to remain in clinical trials as long as possible, reducing the need for retention tools. Whilst a proportion of cancer patients inevitably drop out from clinical trials due to adverse effects of therapy or worsening of their condition, they may no longer be eligible for continuation of the same therapy and so it may not be possible to improve rates of retention in this group. We also noted a relative lack of retention studies for RCTs of circulatory system disorders (Fig. 3). Without an understanding of the underlying mechanisms explaining these observations it is unclear whether the lack of digital retention tools studies in these health conditions represents a major evidence gap, or that retention tools are less useful for certain health conditions.

The map shows that the impact of digital tools for recruitment or retention has mainly been investigated for trial participants who were either patients with a specified health condition or were people eligible for a health promotion intervention. Only one study specifically evaluated digital recruitment of a health professional group: general practitioners being recruited to an online trial to develop and evaluate theory-based interventions to improve antibiotic prescribing [157]. Among the minority or under-served populations (as defined by the study authors), there has been a particular focus on African-American and Hispanic people (eight out of 16 studies on minority or under-served populations), suggesting that these populations are especially challenging to recruit in clinical trials. As with the majority of studies in the map, the research on minority and under-served populations has mainly focussed on recruitment rather than retention. Nevertheless, the authors of a study included in the map commented that they had some difficulty at retaining African American people in their clinical trial of an HIV-prevention intervention in young people [80].

It is unclear why there is an imbalance in the population age distribution in the studies included in the map, i.e. children and older people were represented in some studies of recruitment but not in any of the studies of retention. The age of recruited participants differed between digital and non-digital recruitment methods in a number of the studies (e.g. [78, 83, 103, 107, 129, 140, 152]), indicating that the age of the target population should be taken into consideration when selecting recruitment tools. National statistics show that the proportion of adults who use the Internet (and also social media [170, 171]) is lowest in the over-65 years’ group [171–173], although the proportion of older people using the Internet has risen steadily in recent years [172, 173], suggesting that some digital tools that may currently be out of reach of older people may become increasingly more accessible.

### Combinations of digital and non-digital tools

The studies included in the map evaluated single digital tools, combinations of multiple digital tools or combinations of digital and non-digital tools, with comparators that could be a single digital tool, a single non-digital tool or a combination of multiple digital and/or non-digital tools. The relevance of these digital tool comparisons to clinical trial investigators would depend on whether the investigators’ aim is to supplement existing non-digital recruitment or retention strategies with digital methods; or whether the aim is to employ only digital methods (e.g. if digital tools are considered to be more appropriate or more efficient than standard non-digital recruitment or retention tools, and hence should replace them). Although not specifically captured by our map keywords, we observed that whilst some studies in the map deployed digital tools from the outset of the host trial, others added digital tools after the trial had started, as a response to inadequate recruitment using existing methods.

### Study designs

The problem of how to increase the rates and accuracy of participant recruitment and retention in clinical trials appears well-suited to prospective experimental research, but our systematic map shows that the majority of the primary evaluation studies have been observational in design. A recent paper providing guidance to researchers on the use of social media for recruiting trial participants noted that most of the studies that have shown benefits of social media were observational [174], although observational studies are at increased risk of bias. Direct proof of the effectiveness of digital tools may, therefore, be difficult to establish conclusively unless further experimental studies are conducted. The lack of experimental studies might reflect the idea that SWATs is a relatively new concept [66], and few organisations yet provide funding for these (although this is improving [175]).

The map shows there were many cases where ‘bundles’ of digital and/or non-digital tools were employed for recruitment and/or retention, but the outcomes reported for the digital and non-digital components were often not separable. For example, 12 of the studies in the map employed monetary incentives (e.g. gift vouchers) in addition to their digital tools to encourage participants to join and/or remain in clinical trials. Some studies provided similar monetary incentives to participants in both their digital and non-digital tool study groups (e.g. [104, 106, 114, 126, 158, 162]), whilst other studies provided different monetary incentives for their digital and non-digital tool groups (e.g. [79, 80, 82]). Employing a ‘bundle’ of recruitment or retention tools could have practical relevance (e.g. monetary payment is an integral part of Amazon Mechanical Turk, which was employed as a recruitment tool in one study [82]), but it is important when considering recruitment and retention outcomes to be aware that in some studies estimates of the effectiveness of digital tools could be confounded with the influence of other tools that are present, such as incentives. The authors of one of the studies included in the map acknowledged that they could not rule out the possibility that the observed rates of retention in their study were influenced by the ‘generous’ monetary incentives that they provided [106].

### Outcomes

A key challenge when conducting research is to ensure that appropriate outcomes are measured, but the map suggests that a wide range of recruitment and retention outcomes are relevant and it might not be practical for an individual study to assess all of these. The outcomes that appear to have been considered most important by researchers (Fig. 10) are the rate, reach and costs of recruitment and the efficiency and accuracy of recruitment tools. Among these outcomes, accuracy, efficiency and costs of recruitment were reported in only 18%, 24% and 28% of the recruitment studies, whilst hardly any studies assessed the tool users’ attitudes or satisfaction, despite these being important factors that may determine whether a digital tool is likely to used, and work effectively, in practice. For instance, a tool that achieves a high recruitment reach would be of little value if it inaccurately matches people to the trial eligibility criteria, or is considered burdensome or too expensive to use routinely. Where possible, studies evaluating digital tools should, therefore, measure costs, accuracy and efficiency alongside rates of recruitment or retention, and should also capture key process measures, including the attitudes and satisfaction of end-users of the digital tools.

### Strengths and limitations of our study

Our study benefitted from comprehensive and systematic methods to identify and characterise the evidence for the effectiveness of digital tools for recruitment and retention in RCTs in health. Our inclusion criteria were necessarily broad, to chart the range of digital approaches that have been evaluated, something that has not been achieved by previous evidence syntheses in this area.

We consulted with relevant stakeholders, via our Advisory Board, to ensure that the scope of the project was as relevant as possible to contemporary practice in trial management. The Board advised us on issues such as the inclusion criteria, and the choice of keywords.

We are publishing our map in its entirety, to help other researchers in this area to understand which areas have been well-researched and where the gaps are in the evidence base. We have identified a number of clear recommendations for researchers that should help to improve the quality and relevance of research in this area and, thus, help to fill these important evidence gaps.

There are some potential limitations to this study that should be acknowledged. Whilst our intention was to map studies that investigated RCTs as their host trial, the host-trial design was not stated explicitly in 15 studies (15%); these were included in the map as the host trial appeared likely to be an RCT in the review team’s judgement. Clearer reporting of study designs would be helpful.

A challenge with developing the keywords was that it is difficult to find keywords that are mutually exclusive. For example, a smartphone App could access an Internet site or social media or advertisements (or none of these). A fine-grained systematic map database that splits out all the possible combinations (e.g. distinguishing between: smartphone App with Internet site; smartphone App with social media; smartphone App with advertisements) would be impractically large and would result in relatively small sample sizes per category. We feel that the granularity of the current map is appropriate since it provides useful information whilst maintaining a manageable breadth of keywords.

We limited our searches to the English language, meaning that the scope of the systematic map is specific to English-language studies. We believe it unlikely that the exclusion of non-English-language studies would make our map unrepresentative, as we are not aware of any reasons why English-language and non-English-language studies would differ systematically in the areas of digital tools research covered.

Due to our eligibility criteria requiring studies to have a comparator, some innovative or more recently developed digital approaches or tools for trial recruitment and/or retention that have not been evaluated against a comparator may not have been identified and included in our map. For example, point-of-care clinical trials using electronic health record systems to carry out the research, when a patient presents to, and is being cared for by, a health professional, can potentially be used to enrol and randomise patients, and to collect outcome data. Studies by D’Avolio et al. [176] and van Staa et al. [177] are examples of point-of-care trials that were excluded from our systematic map due to having no comparator.

Our choice of keywords, agreed with our Advisory Board, aimed to enable us to produce a systematic map with a useful level of detail within a specific timescale. A limitation of our map keywords is that they do not go into detail about the methodology of the host trials and primary evaluation studies. For instance, we did not record systematically whether the studies provided a rationale for why a digital approach was appropriate for their particular setting, although we noticed that the reporting of this appeared to be quite variable. We did not assess whether the trials and studies followed the reporting standards for interventional studies such as the Template for Intervention Description and Replication (TIDieR) Checklist [178], or the Guidelines for reporting embedded recruitment trials [179], or whether the studies strictly meet the definition of a SWAT [66] (many of the studies included in the map were conducted before these guidelines were published). However, further keywords could be added to the map database to capture other information on aspects of study rigor, if required. Further keywords could also be added to address specific research questions of interest (for example, to ascertain the extent to which patients and the public were involved in the conception, design and application of digital tools; or to explore how the selection of digital tools is influenced by host-trial endpoints).

## Conclusions

A wide range of digital tools has been evaluated to improve the recruitment and retention of participants in clinical trials. However, few experimental studies, especially randomised controlled studies, have been conducted on digital tools for recruitment or retention, which would limit the availability of robust evidence on the effectiveness of these tools. Further experimental studies of the effectiveness and efficiency of digital tools are needed to ensure that estimates of the effectiveness and efficiency of digital tools are reliable and unbiased.

Our systematic map highlights a number of knowledge gaps where further research would be helpful. These include a lack of research on retention, and a lack of research on certain populations such as children and older people, and on process outcomes (i.e. facilitators and barriers), including the attitudes and satisfaction of the digital tool users. Where possible, studies on digital tools should include process indicators (e.g. measures of costs and acceptability to users) alongside effectiveness and efficiency outcomes, to help understand why digital tools may or may not be effective in particular populations and settings.

Given the complexity of the digital tools’ comparisons identified in the systematic map (bundles of digital and/or non-digital tools were often compared against other combinations of one or more digital and/or non-digital tools), further research would be helpful to clarify which tools work best individually and which work best in combination, for particular populations and health conditions. A question arising from our systematic map is whether a core set of digital tools could be identified that: optimises both recruitment and retention; has utility across a range of health conditions; and is compatible with other tools that are used for the general management and conduct of RCTs (such as for online participant verification, online randomisation, communication and information dissemination between investigators and participants, and data monitoring, checking and tracing).

Since we did not carry out a synthesis of the results of the studies, we are unable to recommend any specific tools for immediate application for trial recruitment or retention. However, the map provides a resource that trial investigators may find useful when considering which tools are available, which tools have been tested in certain populations, and some of the potential limitations of the tools and their comparisons that may need to be considered. Stakeholders may also find the map helpful when considering the prioritisation of which populations, health topics, types of digital tool, and outcomes to focus research on, given that it is unlikely to be possible to conduct studies to cover all populations and health conditions in detail. The map is readily updatable and may be extended in scope, or updated, as necessary to suit the research needs of trial investigators and CTUs. With further development the map could also support guidance (e.g. a checklist) to assist trial investigators and CTUs with the selection of digital tools that are appropriate for their research question.

## Supplementary information

**Additional file 1: Additional Table 1.** Example systematic reviews of digital approaches for recruitment or retention in clinical and health studies.

**Additional file 2: Additional Table 2.** Preferred Reporting Items for Systematic Reviews and Meta-Analyses (PRISMA) 2009 Checklist.

**Additional file 3: Additional Table 3.** List of studies excluded at full-text screening (*N* = 251) (reference list below table).

**Additional file 4: Appendix 1.** Systematic map protocol

**Additional file 5: Appendix 2. ** Search strategies.

**Additional file 6: Appendix 3.** Eligibility screening worksheet.

**Additional file 7: Appendix 4.** List of map keywords with explanations and comments.

**Additional file 8: Appendix 5.** Systematic map database (November 2019).

## Data Availability

All data generated and analysed during this study, including the full systematic map database, are included in this published article and its supplementary information files.

## Abbreviations

CTU(s): Clinical trials unit(s)
HTA: Health Technology Assessment
NHS: National Health Service
NIHR: National Institute for Health Research
PRISMA: Preferred Reporting Items for Systematic Reviews and Meta-Analyses
RCT(s): Randomised controlled trial(s)
SWAT: Study within a trial
UKCRC: UK Clinical Research Collaboration
