# Supplementary material for: Digital tools for the recruitment and retention of participants in randomised controlled trials: a systematic map
Source: Trials. 2020 Jun 5;21:478. doi: 10.1186/s13063-020-04358-3 (PMC7273688; doi:10.1186/s13063-020-04358-3)
Supplement: Supplementary file 1 — Additional file 1: Additional Table 1. Example systematic reviews of digital approaches for recruitment or retention in clinical and health studies. [file 13063_2020_4358_MOESM1_ESM.docx]

**Additional Table 1 Example systematic reviews of digital approaches for recruitment or retention in clinical and health studies**

References are listed below the table

| **Authors, date [reference]** | **Topic** | **Search date** | **Comments** |
| --- | --- | --- | --- |
| Alshaikh et al. 2014 [1] | Social network sites for collecting health data | March 2013 |  |
| Amon et al. 2014 [2] | Facebook for recruiting young people in health research | November 2013 |  |
| Boland et al. 2015 [3] | Recruitment methods for people with cancer or organ failure into clinical trials | February 2014 |  |
| Bonevski et al. 2014 [4] | Strategies for improving health of socially disadvantaged groups | May 2013 |  |
| Brueton et al. 2013 [5] | Methods to improve retention in RCTs or quasi-randomised trials | May 2012 |  |
| Caldwell et al. 2010 [6] | Methods to improve recruitment in RCTs | April 2009 |  |
| Cuggia et al. 2011 [7] | Semi-automatic recruitment systems for clinical trials | August 2009 | Systematic search, not full systematic review |
| Fletcher et al. 2012 [8] | Recruitment methods to assist clinicians in RCTs | March 2011 |  |
| Foster et al. 2011 [9] | Recruitment methods for walking intervention studies | May 2009 |  |
| Köpcke et al. 2014 [10] | Computer-based approaches for patient recruitment into clinical trials | January 2014 | Systematic search, not full systematic review |
| Lam et al. 2016 [11] | Recruitment methods for young people in healthy lifestyle programmes | May 2015 |  |
| Lane et al. 2015 [12] | Online recruitment methods for web-based mobile health studies | October 2014 | Systematic search, not full systematic review |
| Marcano Belisario et al. 2012 [13] | Recruitment methods for smokers in smoking cessation studies | September 2012 |  |
| Park et al. 2013 [14] | Social networking sites for health research on young people | Not reported | Included studies that were published up to 2013 |
| Rosenbaum et al. 2017 [15] | Technology for recruiting minority populations into weight management RCTs | May 2016 |  |
| Thornton et al. 2016 [16] | Facebook for recruiting into health, medical or psychosocial research | March 2015 |  |
| Topolevic-Vranic et al. 2016 [17] | Social media in recruitment for medical research studies | July 2014 | Systematic scoping search, not full systematic review |
| Treweek et al. 2013 [18], 2018 [19] | Methods to improve recruitment in RCTs or quasi-randomised trials | February 2015 |  |
| Whitaker et al. 2017 [20] | Facebook for recruiting participants in health research | March 2017 |  |

**References for Additional Table 1**

1. Alshaikh F, Ramzan F, Rawaf S, Majeed A. Social network sites as a mode to collect health data: a systematic review. Journal of Medical Internet Research. 2014;16(7):e171.

2. Amon KL, Campbell AJ, Hawke C, Steinbeck K. Facebook as a recruitment tool for adolescent health research: a systematic review. Acad Pediatr. 2014;14(5):439-447.e434.

3. Boland J, Currow DC, Wilcock A, Tieman J, Hussain JA, Pitsillides C, et al. A systematic review of strategies used to increase recruitment of people with cancer or organ failure into clinical trials: Implications for palliative care research. Journal of Pain and Symptom Management. 2015;49(4):762-772.

4. Bonevski B, Randell M, Paul C, Chapman K, Twyman L, Bryant J, et al. Reaching the hard-to-reach: a systematic review of strategies for improving health and medical research with socially disadvantaged groups. BMCMedical Research Methodology. 2014;14.

5. Brueton VC, Tierney J, Stenning S, Harding S, Meredith S, Nazareth I, et al. Strategies to improve retention in randomised trials. Cochrane Database of Systematic Reviews. 2013;Issue 12. Art. No.: MR000032:1-127.

6. Caldwell PHY, Hamilton S, Tan A, Craig JC. Strategies for increasing recruitment to randomised controlled trials: systematic review. PLoS Medicine. 2010;7(11): e1000368:1-16.

7. Cuggia M, Besana P, Glasspool D. Comparing semi-automatic systems for recruitment of patients to clinical trials. International Journal of Medical Informatics. 2011;80:371-388.

8. Fletcher B, Gheorghe A, Moore D, Wilson S, Damery S. Improving the recruitment activity of clinicians in randomized controlled trials: a systematic review. BMJ Open. 2012;2:e000496:1-14.

9. Foster CE, Brennan G, Matthews A, McAdam C, Fitzsimons C, Mutrie N. Recruiting participants to walking intervention studies: A systematic review. International Journal of Behavioral Nutrition and Physical Activity. 2011:137.

10. Köpke F, Prokosch H-U. Employing computers for the recruitment into clinical trials: a comprehensive systematic review. J Med Internet Res. 2014;16(7):e161:1-18.

11. Lam E, Partridge SR, Allman-Farinelli M. Strategies for successful recruitment of young adults to healthy lifestyle programmes for the prevention of weight gain: a systematic review. Obes Rev. 2016;17(2):178-200.

12. Lane TS, Armin J, Gordon JS. Online Recruitment Methods for Web-Based and Mobile Health Studies: A Review of the Literature. Journal of Medical Internet Research. 2015;17(7):e183.

13. Marcano Belisario JS, Bruggeling MN, Gunn LH, Brusamento S, Car J. Interventions for recruiting smokers into cessation programmes. Cochrane Database of Systematic Reviews. 2012;12:CD009187.

14. Park BK, Calamaro C. A systematic review of social networking sites: Innovative platforms for health research targeting adolescents and young adults. Journal of Nursing Scholarship. 2013;45(3):256-264.

15. Rosenbaum DL, Piers AD, Schumacher LM, Kase CA, Butryn ML. Racial and ethnic minority enrollment in randomized clinical trials of behavioural weight loss utilizing technology: a systematic review. Obes Rev. 2017;18(7):808-817.

16. Thornton L, Batterham PJ, Fassnacht DB, Kay-Lambkin F, Calear AL, Hunt S. Recruiting for health, medical or psychosocial research using Facebook: Systematic review. Internet Interventions. 2016;4(1):72-81.

17. Topolovec-Vranic J, Natarajan K. The Use of Social Media in Recruitment for Medical Research Studies: A Scoping Review. Journal of Medical Internet Research. 2016;18(11):e286.

18. Treweek S, Lockhart P, Pitkethly M, Cook JA, Kjeldstrøm M, Johansen M, et al. Methods to improve recruitment to randomised controlled trials: Cochrane systematic review and meta-analysis. BMJ Open. 2013;3:e002360:1-24.

19. Treweek S, Pitkethly M, Cook J, Fraser C, Mitchell E, Sullivan F, et al. Strategies to improve recruitment to randomised trials. Cochrane Database of Systematic Reviews. 2018;Issue 2. Art. No.: MR000013:1-185.

20. Whitaker C, Stevelink S, Fear N. The use of Facebook in recruiting participants for health research purposes: a systematic review. Journal of Medical Internet Research. 2017;19(8).
