# Supplementary material for: Digital tools for the recruitment and retention of participants in randomised controlled trials: a systematic map
Source: Trials. 2020 Jun 5;21:478. doi: 10.1186/s13063-020-04358-3 (PMC7273688; doi:10.1186/s13063-020-04358-3)
Supplement: Supplementary file 3 — Additional file 3: Additional Table 3. List of studies excluded at full-text screening (N = 251) (reference list below table). [file 13063_2020_4358_MOESM3_ESM.pdf]

Additional Table 3 List of studies excluded at full-text screening (N=251) (reference list below table)

Study [reference]	Reason for exclusion
[Anon], 2017 [1]	Erratum
Abbate, 2017 [2]	Target not RCT
Adams, 2017 [3]	Target not RCT
Afrin, 2003 [4]	Date pre 2008
Agarwal, 2011 [5]	Meeting abstract
Ahmad, 2005 [6]	Date pre 2008
Ainsworth, 2009 [7]	No relevant outcomes
Ash N, 2001 [8]	Date pre 2008
Bachour, 2015 [9]	Meeting abstract
Bailey, 2011 [10]	Meeting abstract
Baucom, 2014 [11]	Target not RCT
Beattie, 2018 [12]	Article unavailable
Bent, 2014 [13]	No relevant comparator
Bergin, 2010 [14]	No relevant comparator
Blalock, 2017 [15]	Meeting abstract
Blitchtein, 2015 [16]	Meeting abstract
Boland, 2013 [17]	No relevant outcomes
Bold, 2016 [18]	No relevant outcomes
Boles, 2000 [19]	Date pre 2008
Bonadies, 2017 [20]	Target not RCT
Borlikova, 2016 [21]	No relevant outcomes
Bouhnik, 2017 [22]	Meeting abstract
Bouhnik, 2016 [23]	Meeting abstract
Bowen, 2008 [24]	No relevant comparator
Bower, 2017 [25]	Target not RCT
Brabyn, 2014 [26]	No relevant outcomes
Bracken, 2017 [27]	Meeting abstract
Breitfeld, 2003 [28]	Date pre 2008

Study [reference]	Reason for exclusion
Breitfeld, 1999 [29]	Date pre 2008
Bressler, 2016 [30]	Meeting abstract
Brooks, 2009 [31]	No relevant comparator
Brutman, 2018 [32]	Meeting abstract
Bull, 2004 [33]	Date pre 2008
Burgess, 2017 [34]	No relevant comparator
Butte, 2000 [35]	Date pre 2008
Byrd-Bredbenner, 2017 [36]	No relevant outcomes
Cadmus Bertram, 2011 [37]	Not digital
Caesar, 2017 [38]	Meeting abstract
Camacho, 2016 [39]	Meeting abstract
Cambron, 2001 [40]	Date pre 2008
Cantrell, 2012 [41]	No relevant outcomes
Cardozo, 2010 [42]	Target not RCT
Carlson, 1995 [43]	Date pre 2008
Carpenter, 2014 [44]	Meeting abstract
Carroll, 2016 [45]	Meeting abstract
Chan, 2015 [46]	Design
Child, 2014 [47]	Target not RCT
Chipaux, 2010 [48]	Article unavailable
Christian, 2013 [49]	Meeting abstract
Cobb, 2013 [50]	Design
Cobb, 2014 [51]	No relevant comparator
Cowie, 2018 [52]	No relevant comparator
Cunningham, 2017 [53]	No relevant comparator
Cunningham, 2017 [54]	No relevant comparator
D’Avolio, 2012 [55]	No relevant comparator
DeBar, 2009 [56]	No relevant outcomes

Study [reference]	Reason for exclusion
Derrick, 2017 [57]	Target not RCT
Dietrich, 2017 [58]	No relevant outcomes
Dill, 2014 [59]	Target not RCT
Doig, 2012 [60]	Meeting abstract
Elkin, 2016 [61]	Meeting abstract
Elkin, 2016 [62]	Duplicate
Embi, 2005 [63]	Date pre 2008
Embi, 2012 [64]	No relevant outcomes
Etter, 2001 [65]	Date pre 2008
Ezell, 2013 [66]	No relevant outcomes
Fazzino, 2014 [67]	Meeting abstract
Feman, 2008 [68]	Duplicate
Fernandez, 2007 [69]	Date pre 2008
Ferrigno, 2018 [70]	Meeting abstract
Ferris, 2013 [71]	No relevant comparator
Fink, 2003 [72]	Date pre 2008
Fink, 2004 [73]	Date pre 2008
Flores, 2017 [74]	No relevant outcomes
Foster Akard, 2016 [75]	No relevant comparator
Fazzino, 2014 [67]	Meeting abstract
Fraser, 2013 [76]	No relevant comparator
Frawley, 2012 [77]	Meeting abstract
Garcia, 2018 [78]	No relevant outcomes
Garner, 2016 [79]	Meeting abstract
Gatehouse, 2012 [80]	No relevant comparator
Gitlitz, 2017 [81]	Meeting abstract
Glasgow, 2007 [82]	Date pre 2008
Gliddon, 2015 [83]	Meeting abstract
Goff, 2016 [84]	No relevant outcomes
Goldsmith, 2014 [85]	Article unavailable (meeting abstract)

Study [reference]	Reason for exclusion
Goodman, 2011 [86]	No relevant outcomes
Gordon, 2006 [87]	Date pre 2008
Graham, 2006 [88]	Date pre 2008
Groupp, 2005 [89]	Date pre 2008
Grundmeier, 2007 [90]	Date pre 2008
Gupta, 2015 [91]	No relevant outcomes
Harris, 2012 [92]	No relevant comparator
Haslett, 2016 [93]	Meeting abstract
Hawkins, 2014 [94]	No relevant comparator
Hempel, 2013 [95]	Meeting abstract
Huh, 2011 [96]	Meeting abstract
Hui, 2015 [97]	No relevant outcomes
Hutchison, 2007 [98]	Date pre 2008
Irwin, 2017 [99]	Meeting abstract
Jakrot, 2010 [100]	Meeting abstract
Jobling, 2017 [101]	Meeting abstract
Johnson, 1995 [102]	Date pre 2008
Johnson, 2012 [103]	Target not RCT
Johnson, 2015 [104]	Not digital
Jones, 2015 [105]	No relevant comparator
Jones, 2013 [106]	Target not RCT
Jones, 2012 [107]	No relevant outcomes
Joseph, 2016 [108]	No relevant comparator
Joshi, 2017 [109]	Target not RCT
Justin, 2015 [110]	Meeting abstract
Kayrouz, 2016 [111]	Target not RCT
Kelleher, 2018 [112]	No relevant comparator
Kemp, 2014 [113]	Meeting abstract
Kernan, 2009 [114]	Not digital
Kimmick, 2005 [115]	Date pre 2008

Study [reference]	Reason for exclusion
Koca, 2012 [116]	No relevant outcomes
Koenings, 2015 [117]	Meeting abstract
Köpke, 2013 [118]	Target not RCT
Kost, 2015 [119]	No relevant comparator
Krishnamurti, 2016 [120]	No relevant outcomes
Laws, 2016 [121]	Target not RCT
Lawton, 2015 [122]	Meeting abstract
Leavitt, 2017 [123]	No relevant comparator
Lee, 2013 [124]	No relevant outcomes
Leonard, 2014 [125]	No relevant comparator
Leveille, 2008 [126]	No relevant comparator
Lim, 2006 [127]	Date pre 2008
Lobe, 2018 [128]	Meeting abstract
Longbottom, 2012 [129]	No relevant comparator
Lonsdale, 2006 [130]	Date pre 2008
Lonsdale, 2008 [131]	No relevant outcomes
Love, 2010 [132]	Meeting abstract
Lowenstein, 2013 [133]	Meeting abstract
Lucey, 2000 [134]	Date pre 2008
Lutz, 2000 [135]	Date pre 2008
Malhotra, 2012 [136]	No relevant comparator
Markham, 2016 [137]	No relevant outcomes
Martin, 2014 [138]	Meeting abstract
Martin-Biggers, 2016 [139]	Meeting abstract
May, 2013 [140]	Meeting abstract
McCallum, 2014 [141]	No relevant comparator
McGee, 2011 [142]	Design (not primary research)
Meeks, 2013 [143]	Meeting abstract
Metz, 2005 [144]	Date pre 2008
Meunier, 2012 [145]	Meeting abstract

Study [reference]	Reason for exclusion
Meurer, 2018 [146]	Design
Miller, 2010 [147]	No relevant comparator
Mohebati, 2012 [148]	No relevant comparator
Mosis, 2006 [149]	Date pre 2008
Mudano, 2011 [150]	No relevant outcomes
Myers, 2013 [151]	Not digital
Nash, 2016 [152]	Meeting abstract
Nasser, 2017 [153]	Meeting abstract
Nichols, 2017 [154]	Meeting abstract
Nkoy, 2009 [155]	Target not RCT
Nystuen, 2004 [156]	Date pre 2008
Pakhomov, 2005 [157]	Date pre 2008
Papaconstantinou, 1998 [158]	Date pre 2008
Parikh, 2010 [159]	Meeting abstract
Park, 2007 [160]	Date pre 2008
Parkinson, 2013 [161]	Target not RCT
Partridge, 2014 [162]	Meeting abstract
Patel 2007, [163]	Date pre 2008
Pazze, 2011 [164]	Meeting abstract
Pedersen, 2015 [165]	No relevant comparator
Pedersen, 2017 [166]	No relevant comparator
Pena-Robichaux, 2010 [167]	Target not RCT
Plante, 2018 [168]	Meeting abstract
Prescott, 2015 [169]	No relevant comparator
Prescott, 2016 [170]	No relevant outcomes
Quinlan, 2009 [171]	Article unavailable (meeting abstract)
Rait, 2015 [172]	Target not RCT
Ramo, 2014 [173]	No relevant comparator
Ray, 2015 [174]	Target not RCT
Richesson, 2007 [175]	Date pre 2008

Study [reference]	Reason for exclusion
Richesson, 2009 [176]	Target not RCT
Rigney, 2017 [177]	Meeting abstract
Rimel, 2014 [178]	Meeting abstract
Rimel, 2015 [179]	Target not RCT
Ristuccia, 2015 [180]	No relevant comparator
Robinson, 2017 [181]	No relevant comparator
Rocque, 2017 [182]	Target not RCT
Rodrigues, 2011 [183]	Not digital
Rogers, 2017 [184]	Meeting abstract
Rosal, 2010 [185]	No relevant comparator
Roth, 2017 [186]	Meeting abstract
Roth, 2018 [187]	Meeting abstract
Ruffin, 2011 [188]	No relevant comparator
Rukstalis, 2009 [189]	Article unavailable (meeting abstract)
Russo, 2015 [190]	No relevant comparator
Salapatek, 2013 [191]	Meeting abstract
Sanchez, 2018 [192]	No relevant outcomes
Sanossian, 2009 [193]	No relevant comparator
Santoro, 1999 [194]	Date pre 2008
Schmid, 2014 [195]	Article unavailable (meeting abstract)
Schwinn, 2017 [196]	No relevant comparator
Scianni, 2011 [197]	Meeting abstract
Scott, 2016 [198]	No relevant comparator
Seibold-Simpson, 2010 [199]	Not digital
Sellors, 2002 [200]	Date pre 2008
Seroussi, 2003 [201]	Date pre 2008
Seyfried, 2009 [202]	Target not RCT
Shamah, 2016 [203]	Meeting abstract
Shattuck, 2018 [204]	Target not RCT
Shere, 2013 [205]	Meeting abstract

Study [reference]	Reason for exclusion
Short, 2015 [206]	No relevant outcomes
Silva, 1994 [207]	Date pre 2008
Skonnord, 2016 [208]	No relevant outcomes
Smith, 2007 [209]	Date pre 2008
Smith, 2012 [210]	Target not RCT
Spencer, 2015 [211]	Meeting abstract
Spoelstra, 2015 [212]	Meeting abstract
Squiers, 2016 [213]	No relevant comparator
Strehlau, 2017 [214]	No relevant outcomes
Sturgeon, 2018 [215]	No relevant outcomes
Style, 2017 [216]	No relevant comparator
Subasinghe, 2016 [217]	Target not RCT
Suffoletto, 2011 [218]	Meeting abstract
Sumi, 2013 [219]	No relevant comparator
Swain, 2017 [220]	Target not RCT
Takahashi, 2010 [221]	Article unavailable (meeting abstract)
Tanjasiri, 2015 [222]	Not digital
Tehranisa, 2014 [223]	No relevant outcomes
Thew, 2011 [224]	Meeting abstract
Thompson, 2003 [225]	Date pre 2008
Torgerson, 2001 [226]	Date pre 2008
Tortella, 1996 [227]	Date pre 2008
Trotman, 2013 [228]	Meeting abstract
Usher 2014, [229]	No relevant outcomes
van Staa, 2014 [230]	No relevant comparator
Varner, 2016 [231]	Meeting abstract
Vermunt, 2018 [232]	No relevant outcomes
Villanti, 2015 [233]	Target not RCT
Virk, 2015 [234]	Meeting abstract
Wade, 2017 [235]	Meeting abstract

Study [reference]	Reason for exclusion
Walline, 2017 [236]	Meeting abstract
Weiner, 2003 [237]	Date pre 2008
Weintraub, 2016 [238]	Design (not primary research)
Weng, 2015 [239]	Design (not primary research)
Wilbur, 2013 [240]	Not digital
Wilcox, 2011 [241]	Meeting abstract
Williams, 2013 [242]	Meeting abstract
Williams, 2013 [243]	Article unavailable (meeting abstract)
Wise, 2016 [244]	Target not RCT
Wong, 2013 [245]	Not digital
Wu, 2017 [246]	Meeting abstract
Wu, 2018 [247]	Target not RCT
Wu, 2015 [248]	No relevant comparator
Ybarra, 2014 [249]	No relevant comparator
Young, 2014 [250]	Design
Zimmerman, 2018 [251]	No relevant outcomes

Study [reference]	Reason for exclusion

REFERENCES

1. Erratum: Use of digital technology to boost patient recruitment in inflammatory bowel disease clinical trials. J Crohns Colitis. 2017;11(8):1027-1027.
2. Abbate KJ, Hingle MD, Armin J, Giacobbi P, Jr., Gordon JS. Recruiting Women to a Mobile Health Smoking Cessation Trial: Low- and No-Cost Strategies. JMIR Res Protoc. 2017;6(11):e219.
3. Adams D, Handley L, Heald M, Simkiss D, Jones A, Walls E, et al. A Comparison of Two Methods for Recruiting Children with an Intellectual Disability. J Appl Res Intellect Disabil. 2017;30(4):696-704.
4. Afrin LB, Oates JC, Boyd CK, Daniels MS. Leveraging of open EMR architecture for clinical trial accrual. AMIA 2003;Annual Symposium Proceedings/AMIA Symposium.:16-20.

5. Agarwal P, Sia CC, L VG. Novel "bottom-up" recruiting strategies for a Huntington's disease clinical trial at Evergreen Neuroscience Institute, a new HSG investigative site lacking a patient base. Mov Disord. 2011;2):S63.
6. Ahmad F, Gupta R, Kurz M. Real time electronic patient study enrollment system in emergency room. AMIA 2005;Annual Symposium Proceedings/AMIA Symposium.:881.
7. Ainsworth J, Buchan I. Preserving consent-for-consent with feasibility-assessment and recruitment in clinical studies: FARSITE architecture. Stud Health Technol Inform. 2009;147:137-148.
8. Ash N, Ogunyemi O, Zeng Q, Ohno-Machado L. Finding appropriate clinical trials: evaluating encoded eligibility criteria with incomplete data. Proceedings of the AMIA Symposium 2001. 2001:27-31.
9. Bachour CC, Bachmann GA, Foster DC, Wan J, Brown CS. Recruitment of women with provoked vestibulodynia in a clinical trial. Obstetrics and Gynecology. 2015;1):22S.
10. Bailey J, McCarthy O, Carswell K, Murray E, Rait G, Hart G, et al. The sexunzipped website for sexual wellbeing for young people: Early results of a pilot online rct. Journal of Sexual Medicine. 2011;3):126.
11. Baucom RB, Ousley J, Poulose BK, Rosenbloom ST, Jackson GP. Case report: patient portal versus telephone recruitment for a surgical research study. Appl Clin Inform. 2014;5(4):1005-1014.
12. Beattie E, Fielding E, O'Reilly M, Brooks D, MacAndrew M, McCrow J. Recruitment of Individuals With Dementia and Their Carers for Social Research: Lessons Learned From Nine Studies. Res. 2018;11(3):119-128.
13. Bent S, Hendren RL, Zandi T, Law K, Choi JE, Widjaja F, et al. Internet-based, randomized, controlled trial of omega-3 fatty acids for hyperactivity in autism. J Am Acad Child Adolesc Psychiatry. 2014;53(6):658-666.
14. Bergin PS, Ip T, Sheehan R, Frith RW, Sadleir LG, McGrath N, et al. Using the Internet to recruit patients for epilepsy trials: results of a New Zealand pilot study. Epilepsia. 2010;51(5):868-873.

15. Blalock SJ, Solow E, Nowell WB, Woloshin S, Schwartz L, Carpenter DM, et al. A trial testing strategies to enhance patient understanding of drug information: Experience recruiting subjects through an online patient community. Arthritis and Rheumatology Conference: American College of Rheumatology/Association of Rheumatology Health Professionals Annual Scientific Meeting, ACR/ARHP. 2017;69(Supplement 10).
16. Blitchtein D, Zevallos K, Samolski MR, Anthony JC. Recruiting for an SMS smoking cessation intervention in Peru: Pilot study results. Drug and Alcohol Dependence. 2015;146:e223-e224.
17. Boland MR, Miotto R, Gao J, Weng C. Feasibility of Feature-based Indexing, Clustering, and Search of Clinical Trials. Methods Inf Med. 2013;52(5):382-394.
18. Bold KW, Hanrahan TH, O'Malley SS, Fucito LM. Exploring the Utility of Web-Based Social Media Advertising to Recruit Adult Heavy-Drinking Smokers for Treatment. J Med Internet Res. 2016;18(5):e107.
19. Boles M, Getchell WS, Feldman G, McBride R, Hart RG. Primary prevention studies and the healthy elderly: evaluating barriers to recruitment. J Community Health. 2000;25(4):279-292.
20. Bonadies N, Bacher VU, Rovo A, Bohlius J, Stussi G, Gerber B, et al. The swiss MDS registry/biobank: Interim-report of pilot phase from acooperative research platform for personalized medicine in hemato-oncology in switzerland. Leukemia Research. 2017;55 (Supplement 1):S82.
21. Borlikova G, Phillips M, Smith L, O'Neill M. Evolving Classification Models for Prediction of Patient Recruitment in Multicentre Clinical Trials Using Grammatical Evolution. Applications of Evolutionary Computation 19th European Conference, EvoApplications 2016 Proceedings: LNCS 9597. 2016:46-57.
22. Bouhnik Y, Amiot A, Nuzzo A, Attar A, Kondamudi PK, Stefanescu C. Use of digital technology to boost patient recruitment in inflammatory bowel disease clinical trials (vol 11, pg S103, 2017). Journal of Crohns & Colitis. 2017;11(8):1027-1027.
23. Bouhnik Y, Amiot A, Nuzzo A, Mansouri L, Soudan D, Stefanescu C. A pilot study to evaluate the benefits of a recruitment platform, CT-scoutTM in inflammatory bowel diseases (IBD) clinical trials: Linking potential patients to clinical trials. United European Gastroenterology Journal. 2016;4 (5 Supplement 1):A637.

24. Bowen AM, Williams ML, Daniel CM, Clayton S. Internet based HIV prevention research targeting rural MSM: feasibility, acceptability, and preliminary efficacy. J Behav Med. 2008;31(6):463-477.

25. Bower JK, Bollinger CE, Foraker RE, Hood DB, Shoben AB, Lai AM. Active Use of Electronic Health Records (EHRs) and Personal Health Records (PHRs) for Epidemiologic Research: Sample Representativeness and Nonresponse Bias in a Study of Women During Pregnancy. EGEMS (Washington, DC). 2017;5(1):1263-1263.

26. Brabyn S, Adamson J, MacPherson H, Tilbrook H, Torgerson DJ. Short message service text messaging was feasible as a tool for data collection in a trial of treatment for irritable bowel syndrome. J Clin Epidemiol. 2014;67(9):993-1000.

27. Bracken K, Hague W, Wittert G, Keech A, Robledo K. Using phone, SMS and email screening reminders to improve clinical trial recruitment: Results from a sub-study of the t4dm diabetes prevention study. Trials Conference: 4th International Clinical Trials Methodology Conference , ICTMC and the 38th Annual Meeting of the Society for Clinical Trials United Kingdom. 2017;18(Supplement 1).

28. Breitfeld PP, Ullrich F, Anderson J, Crist WM. Web-based decision support for clinical trial eligibility determination in an international clinical trials network. Control Clin Trials. 2003;24(6):702-710.

29. Breitfeld PP, Weisburd M, Overhage JM, Sledge G, Jr., Tierney WM. Pilot study of a point-of-use decision support tool for cancer clinical trials eligibility. J Am Med Inform Assoc. 1999;6(6):466-477.

30. Bressler B, Gunn H, Jang J, Vandermeirsch G, Lim XY, Sutcliffe S, et al. Clinical trial recruitment with social media-what to expect. Gastroenterology. 2016;1):S777-S778.

31. Brooks CJ, Stephens JW, Price DE, Ford DV, Lyons RA, Prior SL, et al. Use of a patient linked data warehouse to facilitate diabetes trial recruitment from primary care. Prim Care Diabetes. 2009;3(4):245-248.

32. Brutman TB, Fisher RL, Stencel L, Ashkenazi I, Bashari D, Lalezari S, et al. How to become a high recruitment site for clinical studies. Haemophilia. 2018;24 (Supplement 5):114.

33. Bull SS, Lloyd L, Rietmeijer C, McFarlane M. Recruitment and retention of an online sample for an HIV prevention intervention targeting men who have sex with men: the Smart Sex Quest Project. AIDS Care. 2004;16(8):931-943.

34. Burgess JD, Kimble RM, Watt K, Cameron CM. The Adoption of Social Media to Recruit Participants for the Cool Runnings Randomized Controlled Trial in Australia. JMIR Res Protoc. 2017;6(10):e200.
35. Butte AJ, Weinstein DA, Kohane IS. Enrolling patients into clinical trials faster using RealTime Recuiting. Proc AMIA Symp. 2000;Annual Symposium.:111-115.
36. Byrd-Bredbenner C, Delaney C, Martin-Biggers J, Koenings M, Quick V. The marketing plan and outcome indicators for recruiting and retaining parents in the HomeStyles randomized controlled trial. Trials. 2017;18 (1) (no pagination)(540).
37. Cadmus Bertram LA, Chung G, Yu H, Salovey P, Irwin M. Feasibility of institutional registry-based recruitment for enrolling newly diagnosed breast cancer patients in an exercise trial. J Phys Act Health. 2011;8(7):955-963.
38. Caesar E, Berkowitz C, Shih C, Shum J, Hackett K, McInnis M, et al. Effectiveness of AlzU.org on recruitment for an Alzheimer's disease prevention clinical trial. Neurology Conference: 69th American Academy of Neurology Annual Meeting, AAN. 2017;88(16 Supplement 1).
39. Camacho MR, Nosheny RL, Truran-Sacrey D, Finley S, Flenniken D, Ulbricht A, et al. Using an internet registry to supplement recruitment efforts for clinical research. Alzheimer's and Dementia. 2016;12 (7 Supplement):P1189.
40. Cambron JA. Recruitment and accrual of women in a randomized controlled trial of spinal manipulation. Journal of Manipulative and Physiological Therapeutics. 2001;24(2):79-83.
41. Cantrell MA, Conte T, Hudson M, Shad A, Ruble K, Herth K, et al. Recruitment and retention of older adolescent and young adult female survivors of childhood cancer in longitudinal research. Oncol Nurs Forum. 2012;39(5):483-490.
42. Cardozo E, Meurer WJ, Smith BL, Holschen JC. Utility of an automated notification system for recruitment of research subjects. Emerg Med J. 2010;27(10):786-787.
43. Carlson RW, Tu SW, Lane NM, Lai TL, Kemper CA, Musen MA, et al. Computer-based screening of patients with HIV/AIDS for clinical-trial eligibility. Online J Curr Clin Trials. 1995;Doc No 179:[3347 words; 3332 paragraphs].
44. Carpenter C, Stark H, Blencowe N, Strong S. The use of social media for recruitment and communication in multicentre collaborative studies. Int J Surg. 2014;3):S51.

45. Carroll MS, Morin M, Dumoulin C, Mayrand MH, Waddell G, Khalife S, et al. E-recruitment for clinical trials in sexual medicine-a rising method for a modern world. Journal of Sexual Medicine. 2016;1):S258.
46. Chan AH, Stewart AW, Harrison J, Camargo Jr CA, Black PN, Mitchell EA. The effect of an electronic monitoring device with audiovisual reminder function on adherence to inhaled corticosteroids and school attendance in children with asthma: a randomised controlled trial. Lancet Respir Med. 2015;3(3):210-219.
47. Child RJ, Mentes JC, Pavlish C, Phillips LR. Using Facebook and participant information clips to recruit emergency nurses for research. Nurse Res. 2014;21(6):16-21.
48. Chipaux M, Grp G. THE GRENAT DATABASE: A NEW TOOL TO RECRUIT EPILEPTIC PATIENTS FOR MULTICENTRE CLINICAL TRIALS. Epilepsia. 2010;51:11-11.
49. Christian K, McGlynn E, Sitas F. Join a research study data base. Asia-Pacific Journal of Clinical Oncology. 2013;3):130-131.
50. Cobb NK, Poirier J. Implementation of an online pragmatic randomized controlled trial: a methodological case study. Transl Behav Med. 2013;3(3):295-303.
51. Cobb NK, Poirier J. Effectiveness of a multimodal online well-being intervention: a randomized controlled trial. Am J Prev Med. 2014;46(1):41-48.
52. Cowie JM, Gurney ME. The Use of Facebook Advertising to Recruit Healthy Elderly People for a Clinical Trial: Baseline Metrics. JMIR Res Protoc. 2018;7(1):e20.
53. Cunningham JA, Godinho A, Kushnir V. Using Mechanical Turk to recruit participants for internet intervention research: experience from recruitment for four trials targeting hazardous alcohol consumption. BMC Med Res Methodol. 2017;17(1):156.
54. Cunningham JA, Godinho A, Kushnir V. Can Amazon's Mechanical Turk be used to recruit participants for internet intervention trials? A pilot study involving a randomized controlled trial of a brief online intervention for hazardous alcohol use. Internet Interv. 2017;10:12-16.
55. D'Avolio L, Ferguson R, Goryachev S, Woods P, Sabin T, O'Neil J, et al. Implementation of the Department of Veterans Affairs’ first point-of-care clinical trial. J Am Med Inform Assoc. 2012;19 e170-e176.

56. DeBar LL, Yarborough BJ, Striegel-Moore RH, Rosselli F, Perrin N, Wilson GT, et al. Recruitment for a guided self-help binge eating trial: Potential lessons for implementing programs in everyday practice settings. Contemp Clin Trials. 2009;30(4):326-333.
57. Derrick JL, Eliseo-Arras RK, Hanny C, Britton M, Haddad S. Comparison of internet and mailing methods to recruit couples into research on unaided smoking cessation. Addict Behav. 2017;75:12-16.
58. Dietrich G, Ertl M, Fette G, Kaspar M, Krebs J, Mackenrodt D, et al. Extending the Query Language of a Data Warehouse for Patient Recruitment. Stud Health Technol Inform. 2017;243:152-156.
59. Dill J, Unson C, Kenyon-Pesce L, Kuchel GA, McElhaney J. Recruitment and retention of healthy and high-risk older adults for a multiyear influenza vaccination study. Conn Med. 2014;78(8):481-486.
60. Doig GS, Simpson F, Sweetman EA. Use of a of a formal study run-in phase to reduce recruitment errors in a multi-centre randomized controlled trial: Is quality better than quantity? Clin. 2012;9 (4):475.
61. Elkin PL, Schlegel DR, Anand E. Recruiting Participants to Local Clinical Trials using Ontology and the IoT. Stud Health Technol Inform. 2016;221:119.
62. Elkin PL, Schlegel DR, Anand E. Recruiting Participants to Local Clinical Trials using Ontology and the IoT. In: Hofdijk J, Seroussi B, Lovis C, Ehrler F, Sieverink F, Ugon A, et al., editors. Transforming Healthcare with the Internet of Things. Studies in Health Technology and Informatics. 2212016. p. 119-119.
63. Embi PJ, Jain A, Clark J, Bizjack S, Hornung R, Harris CM. Effect of a clinical trial alert system on physician participation in trial recruitment. Arch Intern Med. 2005;165(19):2272-2277.
64. Embi PJ, Leonard AC. Evaluating alert fatigue over time to EHR-based clinical trial alerts: findings from a randomized controlled study. J Am Med Inform Assoc. 2012;19(e1):e145-148.
65. Etter JF, Perneger TV. A comparison of cigarette smokers recruited through the Internet or by mail. Int J Epidemiol. 2001;30(3):521-525.
66. Ezell JM, Saltzgaber J, Peterson E, Joseph CL. Reconnecting with urban youth enrolled in a randomized controlled trial and overdue for a 12-month follow-up survey. Clin. 2013;10(5):775-782.

67. Fazzino TL, Rose GL, Helzer JE. Recruiting U.S. and Canadian college students via social media for a web based brief intervention study. Alcoholism: Clinical and Experimental Research. 2014;1):128A.

68. Feman SPC, Nguyen LI, Quilty MT, Kerr CE, Nam BH, Conboy LA, et al. Effectiveness of recruitment in clinical trials: An analysis of methods used in a trial for irritable bowel syndrome patients. Contemp Clin Trials. 2008;29(2):241-251.

69. Fernandez MI, Warren JC, Varga LM, Prado G, Hernandez N, Bowen GS. Cruising in cyber space: comparing Internet chat room versus community venues for recruiting Hispanic men who have sex with men to participate in prevention studies. J Ethn Subst Abuse. 2007;6(2):143-162.

70. Ferrigno C, He J, Cordova CP, Fair J, Shakoor N, Wimmer MA. Exploring the use of online social media advertising to recruit patients in an osteoarthritis clinical trial. Osteoarthritis and Cartilage. 2018;26 (Supplement 1):S271-S272.

71. Ferris M, Norwood V, Radeva M, Gassman JJ, Al-Uzri A, Askenazi D, et al. Patient recruitment into a multicenter randomized clinical trial for kidney disease: report of the focal segmental glomerulosclerosis clinical trial (FSGS CT). Clin Transl Sci. 2013;6(1):13-20.

72. Fink E, Hall LO, Goldgof DB, Goswami BD, Boonstra M, Krischer JP. Experiments on the automated selection of patients for clinical trials. SMC'03 Conference Proceedings 2003 IEEE International Conference on Systems, Man and Cybernetics Conference Theme - System Security and Assurance (Cat No03CH37483). 2003:4541-4545 vol.4545.

73. Fink E, Kokku PK, Nikiforou S, Hall LO, Goldgof DB, Krischer JP. Selection of patients for clinical trials: an interactive web-based system. Artif Intell Med. 2004;31(3):241-254.

74. Flores G, Portillo A, Lin H, Walker C, Fierro M, Henry M, et al. A successful approach to minimizing attrition in racial/ethnic minority, low-income populations. Contemp Clin Trials Commun. 2017;5:168-174.

75. Foster Akard T, Gerhardt CA, Hendricks-Ferguson V, Given B, Friedman DL, Hinds PS, et al. Facebook Advertising to Recruit Pediatric Populations. J Palliat Med. 2016;19(7):692-693.

76. Fraser D, Christiansen BA, Adsit R, Baker TB, Fiore MC. Electronic health records as a tool for recruitment of participants' clinical effectiveness research: Lessons learned from tobacco cessation. Transl Behav Med. 2013;3(3):244-252.

77. Frawley HC, Whitburn LY, Herbison P, Galea MP. Does E-recruitment affect retention and outcomes in a clinical trial? International Urogynecology Journal and Pelvic Floor Dysfunction. 2012;1):S204-S205.
78. Garcia DO, Valdez LA, Bell ML, Humphrey K, Hingle M, McEwen M, et al. A gender- and culturally-sensitive weight loss intervention for Hispanic males: The ANIMO randomized controlled trial pilot study protocol and recruitment methods. Contemp Clin Trials Commun. 2018;9:151-163.
79. Garner E. Use of social media and text messaging outreach in the recruitment of millennials into a phase 3 contraceptive patch study. J Womens Health (Larchmt). 2016;25 (4):A14.
80. Gatehouse CS, Tennen HA, Feinn RS, Abu-Hasaballah KS, Lalla RV. Association between interactive voice response adherence and subject retention in a randomized controlled trial. Contemp Clin Trials. 2012;33(4):589-592.
81. Gitlitz B, Sable-Hunt A, Young S, Kogelnik A, Hicks D, Morosini D, et al. Employing remote web consenting and social media to facilitate enrollment to an international trial on young lung cancer. Journal of Thoracic Oncology. 2017;12 (1 Supplement 1):S1428-S1429.
82. Glasgow RE, Nelson CC, Kearney KA, Reid R, Ritzwoller DP, Strecher VJ, et al. Reach, engagement, and retention in an Internet-based weight loss program in a multi-site randomized controlled trial. J Med Internet Res. 2007;9(2):e11.
83. Gliddon E, Lauder S, Cosgrove V, Grimm D, Dodd S, Suppes T, et al. The pros and cons of online recruitment. Bipolar Disord. 2015;1):85.
84. Goff SL, Youssef Y, Pekow PS, White KO, Guhn-Knight H, Lagu T, et al. Successful Strategies for Practice-Based Recruitment of Racial and Ethnic Minority Pregnant Women in a Randomized Controlled Trial: the IDEAS for a Healthy Baby Study. Journal of racial and ethnic health disparities. 2016;3(4):731-737.
85. Goldsmith JV, Price LL, Driban JB, Harvey WF, Wang C. The cost and effectiveness of various recruitment strategies in a mind body clinical trial among older adults with knee osteoarthritis. Arthritis and Rheumatology. 2014;10):S1019.
86. Goodman L, Sia C, Agarwal P. Successful recruiting strategies at evergreen neuroscience institute, a new huntington study group investigative site. Neurotherapeutics. 2011;8 (1):138.
87. Gordon JS, Akers L, Severson HH, Danaher BG, Boles SM. Successful participant recruitment strategies for an online smokeless tobacco cessation program. Nicotine Tob Res. 2006;8 Suppl 1:S35-41.

88. Graham AL, Bock BC, Cobb NK, Niaura R, Abrams DB. Characteristics of smokers reached and recruited to an internet smoking cessation trial: a case of denominators. Nicotine Tob Res. 2006;8 Suppl 1:S43-48.

89. Groupp E, Haas M, Fairweather A, Ganger B, Attwood M. Recruiting seniors with chronic low back pain for a randomized controlled trial of a self-management program. J Manipulative Physiol Ther. 2005;28(2):97-102.

90. Grundmeier RW, Swietlik M, Bell LM. Research subject enrollment by primary care pediatricians using an electronic health record. AMIA 2007;Annual Symposium Proceedings/AMIA Symposium.:289-293.

91. Gupta A, Calfas KJ, Marshall SJ, Robinson TN, Rock CL, Huang JS, et al. Clinical trial management of participant recruitment, enrollment, engagement, and retention in the SMART study using a Marketing and Information Technology (MARKIT) model. Contemp Clin Trials. 2015;42:185-195.

92. Harris PA, Scott KW, Lebo L, Hassan N, Lightner C, Pulley J. ResearchMatch: a national registry to recruit volunteers for clinical research. Acad Med. 2012;87(1):66-73.

93. Haslett K, Ashcroft L, Bramley R, Wilson B, Livsey J, Asselin M, et al. Using electronic patient records as an effective tool to screen and improve recruitment to a Phase 1 trial in non-small cell lung cancer. Lung Cancer. 2016;91 (Supplement 1):S67.

94. Hawkins MS, Hough LJ, Berger MA, Mor MK, Steenkiste AR, Gao S, et al. Recruitment of veterans from primary care into a physical activity randomized controlled trial: The experience of the VA-STRIDE study. Trials. 2014;15 (1) (no pagination)(11).

95. Hempel E, Millin S. Social media in clinical trial recruitment and retention. Clin. 2013;2):S11.

96. Huh A, Kolls B, Riemen K, Olson D. Using mobile electronic technology increases recruitment in clinical research studies. Neurocritical Care. 2011;1):S28.

97. Hui A, Wong PW, Fu KW. Evaluation of an Online Campaign for Promoting Help-Seeking Attitudes for Depression Using a Facebook Advertisement: An Online Randomized Controlled Experiment. JMIR Ment Health. 2015;2(1):e5.

98. Hutchison C, Cowan C, McMahon T, Paul J. A randomised controlled study of an audiovisual patient information intervention on informed consent and recruitment to cancer clinical trials. Br J Cancer. 2007;97(6):705-711.

99. Irwin RW, Hernandez G, Solinsky CM, Lopez CM, Kono N, Mack WJ, et al. Clinical trial recruitment framework for early Alzheimer's disease. Alzheimer's and Dementia. 2017;13 (7):P1262.

100. Jakrot V, Dalton J, Paton E, Spillane A, Thompson J. Recruitment and follow-up strategies for a large melanoma sentinel node trial with 10 yr follow-up - A coordinator's view from a site perspective. Pigment Cell and Melanoma Research. 2010;23 (6):900.

101. Jobling J, Manton J, Tane M, Perkes S, Pollock L, Bonevski B, et al. Recruiting and consulting for a national evidence-based trial on smoking cessation care for pregnant aboriginal and torres strait islander women: The sistaquit(TM) experience. Asia-Pacific Journal of Clinical Oncology. 2017;13 (Supplement 5):31-32.

102. Johnson FE, Katranides M, Coplin MA, Daly JL, Wade TP, Kraybill WG, et al. Evaluation of alternate messages on an electronic message strip to recruit subjects to a smoking-cessation program. J Cancer Educ. 1995;10(1):31-33.

103. Johnson KA, Peace J. Internet and social network recruitment: two case studies. Ni 2012 (2012). 2012;2012:199.

104. Johnson NA, Kypri K, Latter J, McElduff P, Attia J, Saitz R, et al. Effect of telephone follow-up on retention and balance in an alcohol intervention trial. Prev Med Rep. 2015;2:746-749.

105. Jones R, Lacroix LJ, Nolte K. "Is Your Man Stepping Out?" An Online Pilot Study to Evaluate Acceptability of a Guide-Enhanced HIV Prevention Soap Opera Video Series and Feasibility of Recruitment by Facebook Advertising. J Assoc Nurses AIDS Care. 2015;26(4):368-386.

106. Jones RB, Goldsmith L, Hewson P, Williams CJ. Recruitment to online therapies for depression: pilot cluster randomized controlled trial. J Med Internet Res. 2013;15(3):e45.

107. Jones RB, Goldsmith L, Williams CJ, Kamel Boulos MN. Accuracy of geographically targeted internet advertisements on Google AdWords for recruitment in a randomized trial. J Med Internet Res. 2012;14(3):e84.

108. Joseph CLM, Ownby DR, Zoratti E, Johnson D, Considine S, Bourgeois R, et al. Recruitment experience for a pragmatic randomized controlled trial: Using EMR initiatives and minimizing research infrastructure. Clinical Research and Regulatory Affairs. 2016;33(2-4):25-32.

109. Joshi S, Dunbar K, Taylor P, Sullivan KL, Afzal MM, Song C, et al. Streamlining Participant Recruitment for TBI and PTSD Research Studies. Mil Med. 2017;182(S1):124-127.

110. Justin S, Wilson A, Bailey N, Longson M, Kamauu A. Utilizing electronic medical record networks and advanced analytics for identifying patients for clinical trial recruitment. Pharmacoepidemiology and Drug Safety. 2015;1):587.

111. Kayrouz R, Dear BF, Karin E, Titov N. Facebook as an effective recruitment strategy for mental health research of hard to reach populations. Internet Interv. 2016;Part 1. 4:1-10.

112. Kelleher E, Moreno M, Wilt MP. Recruitment of Participants and Delivery of Online Mental Health Resources for Depressed Individuals Using Tumblr: Pilot Randomized Control Trial. JMIR Res Protoc. 2018;7(4):e95.

113. Kemp K, Reid V, Parker B. A novel virtual biologics clinic leads to improved patient experience, enhanced research recruitment and cost savings: Results of a quality improvement project. Rheumatology (United Kingdom). 2014;1):i77.

114. Kernan WN, Viscoli CM, Demarco D, Mendes B, Shrauger K, Schindler JL, et al. Boosting enrollment in neurology trials with Local Identification and Outreach Networks (LIONs). Neurology. 2009;72(15):1345-1351.

115. Kimmick GG, Peterson BL, Kornblith AB, Mandelblatt J, Johnson JL, Wheeler J, et al. Improving accrual of older persons to cancer treatment trials: a randomized trial comparing an educational intervention with standard information: CALGB 360001. J Clin Oncol. 2005;23(10):2201-2207.

116. Koca M, Husmann G, Jesgarz J, Overath M, Brandts C, Serve H. A Special Query Tool in the Hospital Information System to Recognize Patients and to Increase Patient Numbers for Clinical Trials. In: Mantas J, Andersen SK, Mazzoleni MC, Blobel B, Quaglini S, Moen A, editors. Quality of Life through Quality of Information. Studies in Health Technology and Informatics. 1802012. p. 1180-+.

117. Koenings M, Martin-Biggers J, Byrd-Bredbenner C. Recruiting rct participants: To facebook or not to facebook? FASEB Journal Conference: Experimental Biology. 2015;29(1 Meeting Abstracts).

118. Köpke F, Kraus S, Scholler A, Nau C, Schuttler J, Prokosch H, et al. Secondary use of routinely collected patient data in a clinical trial: An evaluation of the effects on patient recruitment and data acquisition. Int J Med Inf. 2013;82(3):185-192.

119. Kost RG, Corregano LM, Rainer TL, Melendez C, Coller BS. A data-rich recruitment core to support translational clinical research. Clin Transl Sci. 2015;8(2):91-99.

120. Krishnamurti T, Argo N. A Patient-Centered Approach to Informed Consent: Results from a Survey and Randomized Trial. Med Decis Making. 2016;36(6):726-740.

121. Laws RA, Litterbach EK, Denney-Wilson EA, Russell CG, Taki S, Ong KL, et al. A Comparison of Recruitment Methods for an mHealth Intervention Targeting Mothers: Lessons from the Growing Healthy Program. J Med Internet Res. 2016;18(9):e248.

122. Lawton S, Wathall S. Innovative methods for optimising clinical trial recruitment and retention within primary care. Trials Conference: 3rd International Clinical Trials Methodology Conference United Kingdom. 2015;16(Supplement 2).

123. Leavitt L, Abroms L, Johnson P, Schindler-Ruwisch J, Bushar J, Singh I, et al. Recruiting pregnant smokers from Text4baby for a randomized controlled trial of Quit4baby. Transl Behav Med. 2017;7(2):157-165.

124. Lee Y, Krishnamoorthy S, Dinakarpandian D. A Semantic Framework for Intelligent Matchmaking for Clinical Trial Eligibility Criteria. ACM Trans Intell Syst Technol. 2013;4(4).

125. Leonard A, Hutchesson M, Patterson A, Chalmers K, Collins C. Recruitment and retention of young women into nutrition research studies: practical considerations. Trials. 2014;15:23.

126. Leveille SG, Huang A, Tsai SB, Weingart SN, Iezzoni LI. Screening for chronic conditions using a patient internet portal: recruitment for an internet-based primary care intervention. J Gen Intern Med. 2008;23(4):472-475.

127. Lim HJ, Lin CJ, Liu Y, Chee W, Im EO. Effective strategies for recruiting of Asian cancer patients in internet research. Stud Health Technol Inform. 2006;122:882.

128. Lobe M, Staubert S, Goldberg C, Haffner I, Winter A. Towards Phenotyping of Clinical Trial Eligibility Criteria. Studies in health technology and informatics. 2018;248:293-299.

129. Longbottom ME, Roberts JN, Tom M, Hughes SE, Howard VJ, Sheffet AJ, et al. Interventions to increase enrollment in a large multicenter phase 3 trial of carotid stenting vs. endarterectomy. Int j. 2012;7(6):447-453.

130. Lonsdale D, Tustison C, Parker C, Embley DW. Formulating queries for assessing clinical trial eligibility. Natural Language Processing and Information Systems 11th International Conference on Applications of Natural Language to Information Systems, NLDB 2006 Proceedings (Lecture Notes in Computer Science Vol3999). 2006:82-93.
131. Lonsdale DW, Tustison C, Parker CG, Embley DW. Assessing clinical trial eligibility with logic expression queries. Data Knowl Eng. 2008;66(1):3-17.
132. Love SM. A new resource to accelerate research into the cause and prevention of breast cancer: The Love/Avon Army of Women. Cancer Prevention Research Conference: AACR International Conference on Frontiers in Cancer Prevention Research Philadelphia, PA United States Conference Publication:. 2010;3(12 SUPPL. 2).
133. Lowenstein LM, DeWindt L, Sutter E, Barnett S, Pearson TA. Community engagement makes for recruitment success into a randomized controlled intervention trial for deaf adults. Circulation Conference: American Heart Association's Epidemiology and Prevention/Physical Activity, Nutrition and Metabolism. 2013;127(12 Meeting Abstracts).
134. Lucey JF, Suresh GK, Kappas A. Crigler-Najjar syndrome, 1952-2000: learning from parents and patients about a very rare disease and using the internet to recruit patients for studies. Pediatrics. 2000;105(5):1152-1153.
135. Lutz S, Henkind SJ. Recruiting for clinical trials on the Web. Healthplan. 2000;41(5):36-43.
136. Malhotra S, Musgrave SD, Pinnock H, Price D, Ryan DP. The challenge of recruiting in primary care for a trial of telemonitoring in asthma: an observational study. Pragmat. 2012;3:51-55.
137. Markham S. Development of an online clinical trial recruitment portal for the NIHR mental health BRC. Res Involv Engagem. 2016;2:11.
138. Martin SS, Feldman DI, Blumenthal RS, Jones SR, Ratchford EV, Coresh J, et al. Recruitment success of the mactive physical activity mhealth trial: Can convenience increase enrollment of eligible patients and women into RCTs? Circulation Conference: American Heart Association's. 2014;130(SUPPL. 2).
139. Martin-Biggers JT, Delaney C, Koenings M, Byrd-Bredbenner C. HomeStyles: Recruitment strategies for a childhood obesity prevention randomized controlled trial. FASEB Journal Conference: Experimental Biology. 2016;30(Meeting Abstracts).

140. May NB, Winstead-Derlega C, Zimmerman P, Plews-Ogan M, Nees M, Dillingham R, et al. Recruiting for a text message intervention for rural african-american women with diabetes. Diabetes. 2013;1):A178.

141. McCallum GB, Versteegh LA, Morris PS, McKay CC, Jacobsen NJ, White AV, et al. Mobile phones support adherence and retention of indigenous participants in a randomised controlled trial: strategies and lessons learnt. BMC Public Health. 2014;14:622.

142. McGee MK. Provider Uses E-records To Recruit For Clinical Trials. InformationWEEK. 2011(1309):90-90.

143. Meeks B. Improved recruitment rates in a randomized controlled trial of acute myocardial infarction through use of a web-based interface for central randomization. Clin. 2013;2):S64.

144. Metz JM, Coyle C, Hudson C, Hampshire M. An Internet-based cancer clinical trials matching resource. J Med Internet Res. 2005;7(3):e24.

145. Meunier C, Jennings D, Hunter C, Sherer T. Fox trial finder: An innovative web-based trial matching tool to facilitate clinical trial recruitment. Neurology Conference: 64th American Academy of Neurology Annual Meeting New Orleans, LA United States Conference Publication:. 2012;78(1 Meeting Abstract).

146. Meurer WJ, Brown D, Skolarus L. Reach out: An exploratory randomized clinical trial of emergency department-based hypertension management. Stroke Conference: American Heart Association/American Stroke Association. 2018;49(Supplement 1).

147. Miller DM, Fox R, Atreja A, Moore S, Lee JC, Fu AZ, et al. Using an automated recruitment process to generate an unbiased study sample of multiple sclerosis patients. Telemedicine Journal & E-Health. 2010;16(1):63-68.

148. Mohebati A, Knutson A, Zhou XK, Smith JJ, Brown PH, Dannenberg AJ, et al. A web-based screening and accrual strategy for a cancer prevention clinical trial in healthy smokers. Contemp Clin Trials. 2012;33(5):942-948.

149. Mosis G, Dieleman JP, Stricker B, van der Lei J, Sturkenboom MC. A randomized database study in general practice yielded quality data but patient recruitment in routine consultation was not practical. J Clin Epidemiol. 2006;59(5):497-502.

150. Mudano A, Gary L, Melton M, Warriner A, Harrington TM, Kilgore M, et al. Methods to simplify recruiting for pragmatic clinical trials (PCTS) in osteoporosis in the community: Ipad vs. Interactive voice response (IVRS) systems. Journal of Bone and Mineral Research Conference. 2011;26(SUPPL. 1).

151. Myers K, Vander Stoep A, Lobdell C. Feasibility of conducting a randomized controlled trial of telemental health with children diagnosed with attention-deficit/hyperactivity disorder in underserved communities. J Child Adolesc Psychopharmacol. 2013;23(6):372-378.
152. Nash E, Sharman JE. Facebook advertising is successful for participant recruitment into a national blood pressure clinical trial. Hypertension. 2016;67(5):E13-E13.
153. Nasser N, Skurnik G, Stuart J, Katz J, Chen G, Roche AT, et al. Social media and web-based recruitment strategies for a preeclampsia postpartum lifestyle intervention trial. Endocrine Reviews Conference: 99th Annual Meeting of the Endocrine Society, ENDO. 2017;38(3 Supplement 1).
154. Nichols G, Granowitz C, Reynolds K, Philip S, Fazio S. The potential of electronic health record data to optimize recruitment efficiency in cardiovascular outcome trials. Journal of Clinical Lipidology. 2017;11 (3):796-797.
155. Nkoy FL, Wolfe D, Hales JW, Lattin G, Rackham M, Maloney CG. Enhancing an existing clinical information system to improve study recruitment and census gathering efficiency. AMIA Annu Symp Proc. 2009;2009:476-480.
156. Nystuen P, Hagen KB. Telephone reminders are effective in recruiting nonresponding patients to randomized controlled trials. J Clin Epidemiol. 2004;57(8):773-776.
157. Pakhomov SV, Buntrock J, Chute CG. Prospective recruitment of patients with congestive heart failure using an ad-hoc binary classifier. J Biomed Inform. 2005;38(2):145-153.
158. Papaconstantinou C, Theocharous G, Mahadevan S. An expert system for assigning patients into clinical trials based on Bayesian networks. J Med Syst. 1998;22(3):189-202.
159. Parikh PS, Ratanamaneechat S, George D, Comhair SA, Baran J, Hughes R, et al. Recruitment and enrollment of asthmatics in a phase II clinical trial. American Journal of Respiratory and Critical Care Medicine Conference: American Thoracic Society International Conference, ATS. 2010;181(1 MeetingAbstracts).
160. Park ER, Quinn VP, Chang Y, Regan S, Loudin B, Cummins S, et al. Recruiting pregnant smokers into a clinical trial: Using a network-model managed care organization versus community-based practices. Prev Med. 2007;44(3):223-229.

161. Parkinson S, Bromfield L. Recruiting young adults to child maltreatment research through Facebook: A feasibility study. Child Abuse and Neglect. 2013;37(9):716-720.
162. Partridge SR, Hebdren L, Balestracci K, Wong A, Phongsavan P, Denney-Wilson E, et al. Recruiting young adults into the TXT2BFiT trial for prevention of weight gain: Effectiveness and cost strategies. Obesity Research and Clinical Practice. 2014;1):77.
163. Patel C, Cimino J, Dolby J, Fokoue A, Kalyanpur A, A K, et al. Matching patient records to clinical trials using ontologies. In: Aberer K, Choi K-S, Noy N, Allemang D, Lee KI, Nixon L, et al., editors. The Semantic Web Proceedings 6th International Semantic Web Conference, 2nd Asian Semantic Web Conference Berlin: Springer-Verlag; 2007. p. 816-829.
164. Pazze LMD, Chowdhury S, Eberling JL, Meunier CC, Brooks DW. Innovative web-based matching service Fox Trial Finder as a means to improve clinical trial recruitment. Mov Disord. 2011;26:S135-S136.
165. Pedersen ER, Helmuth ED, Marshall GN, Schell TL, PunKay M, Kurz J. Using facebook to recruit young adult veterans: online mental health research. JMIR Res Protoc. 2015;4(2):e63.
166. Pedersen ER, Naranjo D, Marshall GN. Recruitment and retention of young adult veteran drinkers using Facebook. PLoS ONE. 2017;12(3):e0172972.
167. Pena-Robichaux V, Watson AJ. Recruiting subjects for research studies in the era of Web 2.0. J Am Acad Dermatol. 2010;63(3):e69-70.
168. Plante TB, Juraschek SP, Gleason K, Charleston JB, McArthur K, Dennison-Himmelfarb C, et al. Recruitment of seniors to a randomized con-trolled trial using electronic health record-enabled patient portal recruitment messages. J Gen Intern Med. 2018;33 (2 Supplement 1):328.
169. Prescott T, Phillips GL, Bull SS, Parsons JT, Mustanski B, Ybarra ML. Reaching gay, bisexual, and queer adolescent males: Development and refinement of an online recruitment strategy. Journal of Sexual Medicine. 2015;12 (Supplement 5):325-326.
170. Prescott TL, Phillips Ii G, DuBois LZ, Bull SS, Mustanski B, Ybarra ML. Reaching Adolescent Gay, Bisexual, and Queer Men Online: Development and Refinement of a National Recruitment Strategy. J Med Internet Res. 2016;18(8):e200.
171. Quinlan NP, Rukstalis MR, Diewald LK, Hoffer K, Berkowitz RI, Faith MS. Electronic Health Record (EHR) Recruitment for Early Childhood Overweight Intervention Research. Obesity (Silver Spring). 2009;17:S190-S190.

172. Rait MA, Prochaska JJ, Rubinstein ML. Recruitment of adolescents for a smoking study: use of traditional strategies and social media. Transl Behav Med. 2015;5(3):254-259.

173. Ramo DE, Rodriguez TMS, Chavez K, Sommer MJ, Prochaska JJ. Facebook recruitment of young adult smokers for a cessation trial: Methods, metrics, and lessons learned. Internet Interv. 2014;1(2):58-64.

174. Ray B, Aphinyanaphongs Y, Heffron S. Text Classification-Based Automatic Recruitment of Patients for Clinical Trials: A Silver Standards-Based Case Study. 2015 International Conference on Healthcare Informatics (ICHI) Proceedings. 2015:28-33.

175. Richesson RL, Young K, Lloyd J, Adams T, Guillette H, Malloy J, et al. An automated communication system in a Contact Registry for persons with rare diseases: tools for retaining potential clinical research participants. AMIA 2007;Annual Symposium Proceedings/AMIA Symposium.:1094.

176. Richesson RL, Lee HS, Cuthbertson D, Lloyd J, Young K, Krischer JP. An automated communication system in a contact registry for persons with rare diseases: scalable tools for identifying and recruiting clinical research participants. Contemp Clin Trials. 2009;30(1):55-62.

177. Rigney G, Weiss S, Chambers CT, Godbout R, Gruber R, Hall W, et al. Better nights, better days: relative success of digital, media and traditional recruitment methods for an e-health intervention for children with behavioural insomnia. Sleep Med. 2017;40 (Supplement 1):e345.

178. Rimel BJ, Lester J, Dang C, Sabacan L, Park D, Karlan BY. A novel clinical trial recruitment strategy for women's cancers. Gynecol Oncol. 2014;1):142-143.

179. Rimel BJ, Lester J, Sabacan L, Park D, Bresee C, Dang C, et al. A novel clinical trial recruitment strategy for women's cancer. Gynecol Oncol. 2015;138(2):445-448.

180. Ristuccia R, Badoux XC, Gibson M, Huseincehajic A, Trotman J, Haematology Clinical Res N. Increasing Cross-Referral and Recruitment to Clinical Trials: A New Approach. Blood. 2015;126(23).

181. Robinson BN, Newman AF, Tefera E, Herbolsheimer P, Nunes R, Gallagher C, et al. Video intervention increases participation of black breast cancer patients in therapeutic trials. NPJ Breast Cancer. 2017;3:36.

182. Rocque GB, Halilova KI, Varley AL, Williams CP, Taylor RA, Masom DG, et al. Feasibility of a Telehealth Educational Program on Self-Management of Pain and Fatigue in Adult Cancer Patients. J Pain Symptom Manage. 2017;53(6):1071-1078.

183. Rodrigues MJ, Haberer S, Dionysopoulos D, Barriere J, Wassermann J, Tazi Y, et al. Internet to boost patient accrual in oncology trials? A multiinstitutional AERIO study. Ann Oncol. 2011;22(2):490-491.

184. Rogers A, Mackenzie I, Rorie D, MacDonald TM. Successful recruitment to a large online randomised trial: The TIME study. Trials Conference: 4th International Clinical Trials Methodology Conference , ICTMC and the 38th Annual Meeting of the Society for Clinical Trials United Kingdom. 2017;18(Supplement 1).

185. Rosal MC, White MJ, Borg A, Scavron J, Candib L, Ockene I, et al. Translational research at community health centers: Challenges and successes in recruiting and retaining low-income latino patients with type 2 diabetes into a randomized clinical trial. Diabetes Educ. 2010;36(5):733-749.

186. Roth T, O'Daly S, Fenton M, Dowd S, Cassidy N, Allen J, et al. Effectiveness of recruiting type 1 narcolepsy patients via internet based pre-selection system. Sleep Med. 2017;40 (Supplement 1):e283.

187. Roth T, O'Daly S, Fenton M, Dowd S, Cassidy N, Allen J, et al. Effectiveness of recruiting type 1 narcolepsy patients via internet based pre-selection system. Sleep. 2018;41 (Supplement 1):A129-A130.

188. Ruffin IMT, Nease Jr DE. Using patient monetary incentives and electronically derived patient lists to recruit patients to a clinical trial. Journal of the American Board of Family Medicine. 2011;24(5):569-575.

189. Rukstalis MR, Rivera M, Anderer T, Bishop-Gilyard CT, Cochran W, Berkowitz RI. Use of Electronic Health Records to Recruit for Two-Site Adolescent Weight Management Randomized Behavioral Intervention Trial. Obesity (Silver Spring). 2009;17:S192-S193.

190. Russo R, Coultas D, Ashmore J, Peoples J, Sloan J, Jackson BE, et al. Chronic obstructive pulmonary disease self-management activation research trial (COPD-SMART): Results of recruitment and baseline patient characteristics. Contemp Clin Trials. 2015;41:192-201.

191. Salapatek AM, Buck J, Patel P. A case study in effective innovative strategic multi-modal recruitment strategies in difficult-to-recruit patient populations: Study of safety and tolerability of novel SLIT in an adolescent population. World Allergy Organization Journal Conference. 2013;7(SUPPL. 1).

192. Sanchez VA, Hall DA, Millar B, Escabi CD, Sharman A, Watson J, et al. Recruiting ENT and Audiology patients into pharmaceutical trials: evaluating the multi-centre experience in the UK and USA. Int J Audiol. 2018:1-12.

193. Sanossian N, Starkman S, Liebeskind DS, Ali LK, Restrepo L, Hamilton S, et al. Simultaneous ring voice-over-Internet phone system enables rapid physician elicitation of explicit informed consent in prehospital stroke treatment trials. Cerebrovasc Dis. 2009;28(6):539-544.

194. Santoro E, Nicolis E, Franzosi MG. Telecommunication technology for the management of large scale clinical trials: the Gissi experience. Computer Methods and Programs in Biomedicine. 1999;60(3):215-223.

195. Schmid A, Harvey WF, Price LL, Wang C. Cost-effectiveness and yield of different recruitment strategies utilized in an exercise trial of fibromyalgia patients. Arthritis and Rheumatology. 2014;10):S1019-S1020.

196. Schwinn T, Hopkins J, Schinke SP, Liu X. Using Facebook ads with traditional paper mailings to recruit adolescent girls for a clinical trial. Addict Behav. 2017;65:207-213.

197. Scianni A, Teixeira-Salmela LF, Ada L. Feasibility of a randomized clinical trial of muscle strengthening with stroke survivors: Recruitment, attendance, and adherence. Physiotherapy (United Kingdom). 2011;1):eS1117-eS1118.

198. Scott IU, VanVeldhuisen PC, Ip MS, Blodi BA, Oden NL, Figueroa M, et al. SCORE2 Report 1: Techniques to Optimize Recruitment in Phase III Clinical Trials of Patients With Central Retinal Vein Occlusion. Am J Ophthalmol. 2016;170:25-31.

199. Seibold-Simpson S, Morrison-Beedy D. Avoiding early study attrition in adolescent girls: impact of recruitment contextual factors. West J Nurs Res. 2010;32(6):761-778.

200. Sellors J, Cosby R, Trim K, Kaczorowski J, Howard M, Hardcastle L, et al. Recruiting family physicians and patients for a clinical trial: Lessons learned. Fam Pract. 2002;19(1):99-104.

201. Seroussi B, Bouaud J. Using OncoDoc as a computer-based eligibility screening system to improve accrual onto breast cancer clinical trials. Artif Intell Med. 2003;29(1-2):153-167.

202. Seyfried L, Hanauer DA, Nease D, Albeiruti R, Kavanagh J, Kales HC. Enhanced identification of eligibility for depression research using an electronic medical record search engine. Int J Med Inf. 2009;78:e13-e18.

203. Shamah CJ, Saphner TJ. Effect on clinical trial participation by integration of a clinical pathway program into an electronic health record (EHR). J Clin Oncol. 2016;34(7).

204. Shattuck D, Haile LT, Simmons RG. Lessons From the Dot Contraceptive Efficacy Study: Analysis of the Use of Agile Development to Improve Recruitment and Enrollment for mHealth Research. JMIR Mhealth Uhealth. 2018;6(4):e99.

205. Shere M, Koren G. The role of social media in recruiting for clinical trials in pregnancy. FASEB Journal Conference: Experimental Biology. 2013;27(Meeting Abstracts).

206. Short CE, Rebar AL, Vandelanotte C. Do personalised e-mail invitations increase the response rates of breast cancer survivors invited to participate in a web-based behaviour change intervention? A quasi-randomised 2-arm controlled trial.[Erratum appears in BMC Med Res Methodol. 2015;15:102; PMID: 26589396]. BMC Med Res Methodol. 2015;15:66.

207. Silva MI, Abbott P, Petrucci K, Canfield K, Muir J. Improving the efficiency of patient recruitment with an automated telephone screening system in a client-server environment. Seventeenth Annual Symposium on Computer Applications in Medical Care Patient-Centered Computing. 1994:41-45.

208. Skonnord T, Steen F, Skjeie H, Fetveit A, Brekke M, Klovning A. Survey Email Scheduling and Monitoring in eRCTs (SESAMe): A Digital Tool to Improve Data Collection in Randomized Controlled Clinical Trials. J Med Internet Res. 2016;18(11):e311.

209. Smith KS, Eubanks D, Petrik A, Stevens VJ. Using web-based screening to enhance efficiency of HMO clinical trial recruitment in women aged forty and older. Clin. 2007;4(1):102-105.

210. Smith BD, Kalayil EJ, Patel-Larson A, Chen B, Vaughan M. Retaining clients in an outcome monitoring evaluation study: HIV prevention efforts in community settings. Eval Program Plann. 2012;35(1):16-24.

211. Spencer J, Wilson A, Bailey N, Longson MS, Kamauu A. Utilizing electronic medical record networks for identifying patients for clinical trial recruitment. Value in Health. 2015;18 (3):A18.

212. Spoelstra S, Sikorskii A, Given C, Coursaris CK, Majumder A, DeKoekkoek T, et al. Recruitment and enrollment of older adult into mobile phone text message clinical trials. Gerontologist. 2015;55:477-477.

213. Squiers L, Brown D, Parvanta S, Dolina S, Kelly B, Dever J, et al. The SmokefreeTXT (SFTXT) Study: Web and Mobile Data Collection to Evaluate Smoking Cessation for Young Adults. JMIR Res Protoc. 2016;5(2):e134.

214. Strehlau V, Torchalla I, Patterson M, Moniruzzaman A, Laing A, Addorisio S, et al. Recruitment and retention of homeless individuals with mental illness in a housing first intervention study. Contemp Clin Trials Commun. 2017;7:48-56.
215. Sturgeon KM, Hackley R, Fornash A, Dean LT, Laudermilk M, Brown JC, et al. Strategic recruitment of an ethnically diverse cohort of overweight survivors of breast cancer with lymphedema. Cancer. 2018;124(1):95-104.
216. Style S, Beard BJ, Harris-Fry H, Sengupta A, Jha S, Shrestha BP, et al. Experiences in running a complex electronic data capture system using mobile phones in a large-scale population trial in southern Nepal. Glob Health Action. 2017;10(1):1330858.
217. Subasinghe AK, Nguyen M, Wark JD, Tabrizi SN, Garland SM. Targeted Facebook Advertising is a Novel and Effective Method of Recruiting Participants into a Human Papillomavirus Vaccine Effectiveness Study. JMIR Res Protoc. 2016;5(3):e154.
218. Suffoletto B, Kristan J, Callaway C. Use of text-messaging drinking assessments to minimize missing data in an interventional clinical trial. Ann Emerg Med. 2011;1):S197.
219. Sumi E, Teramukai S, Yamamoto K, Satoh M, Yamanaka K, Yokode M. The correlation between the number of eligible patients in routine clinical practice and the low recruitment level in clinical trials: a retrospective study using electronic medical records. Trials. 2013;14:426.
220. Swain MS, Kamper SJ, Maher CG, Latimer J, Broderick C, McKay D, et al. Short-term Clinical Course of Knee Pain in Children and Adolescents: A Feasibility Study Using Electronic Methods of Data Collection. Physiother Res Int. 2017;22(4).
221. Takahashi Y, Nishida Y, Asai S. Utilization of clinical data warehouse in clinical studies and clinical trial recruitment screening. Journal of Pharmacological Sciences. 2010;112:176P-176P.
222. Tanjasiri SP, Weiss JW, Santos L, Flores P, Flores P, Lacsamana JD, et al. CBPR-Informed Recruitment and Retention Adaptations in a Randomized Study of Pap Testing Among Pacific Islanders in Southern California. Prog. 2015;9(3):389-396.
223. Tehranisa JS, Meurer WJ. Can response-adaptive randomization increase participation in acute stroke trials? Stroke. 2014;45(7):2131-2133.
224. Thew S, Leeming G, Ainsworth J, Gibson M, Buchan I. FARSITE: Evaluation of an automated trial feasibility assessment and recruitment tool. Trials Conference: Clinical Trials Methodology Conference. 2011;12(SUPPL. 1).

225. Thompson DS, Oberteuffer R, Dorman T. Sepsis alert and diagnostic system: integrating clinical systems to enhance study coordinator efficiency. Comput Inform Nurs. 2003;21(1):22-26; quiz 27-28.
226. Torgerson JS, Arlinger K, Kappi M, Sjostrom L. Principles for enhanced recruitment of subjects in a large clinical trial. the XENDOS (XENical in the prevention of Diabetes in Obese Subjects) study experience. Control Clin Trials. 2001;22(5):515-525.
227. Tortella BJ, Lavery RF, Quadrel M, Cody RP, Heyt G. Use of on-line medical command to randomize patients in a prehospital research study. Prehospital Disaster Med. 1996;11(1):55-58; discussion 58-59.
228. Trotman J, Badoux X, Huseincehajic A, Gambrill M, LeGall A, Daly M, et al. Clintrial Refer- a Mobile App To Connect Patients With Local Clinical Trials. Blood. 2013;122(21).
229. Usher D, Frye V, Shinnick J, Greene E, Baez E, Benitez J, et al. Recruitment by a geospatial networking application for research and practice: the New York City experience. J Acquir Immune Defic Syndr. 2014;67(5):e143-145.
230. van Staa TP, Dyson L, McCann G, Padmanabhan S, Belatri R, Goldacre B, et al. The opportunities and challenges of pragmatic point-of-care randomised trials using routinely collected electronic records: evaluations of two exemplar trials. Health Technol Assess. 2014;18(43):1-146.
231. Varner C, McLeod SL, Nahiddi N, Borgundvaag B. Text messaging research participants as a follow-up strategy to decrease emergency department study attrition. Canadian Journal of Emergency Medicine. 2016;18 (Supplement 1):S59.
232. Vermunt L, Veal CD, Ter Meulen L, Chrysostomou C, van der Flier W, Frisoni GB, et al. European Prevention of Alzheimer's Dementia Registry: Recruitment and prescreening approach for a longitudinal cohort and prevention trials. Alzheimers Dement. 2018;14(6):837-842.
233. Villanti AC, Jacobs MA, Zawistowski G, Brookover J, Stanton CA, Graham AL. Impact of Baseline Assessment Modality on Enrollment and Retention in a Facebook Smoking Cessation Study. J Med Internet Res. 2015;17(7):e179.
234. Virk H, Murff W, Castle C, Paniagua S, Rosen KD, Potter JS. Clinical trials in the Facebook age: using social media to recruit individuals with addiction. Ann Behav Med. 2015;49:S188-S188.
235. Wade A, Crawford GM. The effectiveness and cost-effectiveness of different methods of recruiting patients to a pain study. Value in Health. 2017;20 (9):A550.

236. Walline JJ, Jones-Jordan LA, Shaw KJ, Myers JA, Cardenas LL, Nixon AD, et al. Recruitment costs for a randomized clinical trial. Investigative Ophthalmology and Visual Science Conference. 2017;58(8).
237. Weiner DL, Butte AJ, Hibberd PL, Fleisher GR. Computerized recruiting for clinical trials in real time. Ann Emerg Med. 2003;41(2):242-246.
238. Weintraub K. Social media ups clinical trial enrollment. Cancer Discov. 2016;6(8):808-809.
239. Weng C. Optimizing Clinical Research Participant Selection with Informatics. Trends in Pharmacological Sciences. 2015;36(11):706-709.
240. Wilbur J, Buchholz SW, Ingram DM, Braun LT, Johnson TJ, Fogg L, et al. Effectiveness, efficiency, duration, and costs of recruiting for an African American women's lifestyle physical activity program. Res Nurs Health. 2013;36(5):487-499.
241. Wilcox CS, Oskooilar N, Tong ML, Morrissey JL, De Francisco DF, Henry MM, et al. Do current internet-based patient recruitment methods impact patient retention and completion rates in depression studies and are these metrics truly indicative of positive outcomes? Neuropsychopharmacology. 2011;1):S359-S360.
242. Williams JW, Taylor CB, Romaniuk H, Cini K, Sawyer S, Taylor K. Recruitment challenges: A randomised trial of a community based obesity intervention for adolescents. Turk Pediatri Arsivi. 2013;2):113.
243. Williams JW, Taylor CB, Romaniuk H, Cini K, Sawyer S, Taylor K. Recruitment Challenges: A Randomised Trial of a Community Based Obesity Intervention for Adolescents. Turkish Archives of Pediatrics. 2013;48 (Suppl 2):113.
244. Wise T, Arnone D, Marwood L, Zahn R, Lythe KE, Young AH. Recruiting for research studies using online public advertisements: Examples from research in affective disorders. Neuropsychiatric Disease and Treatment. 2016;12:279-285.
245. Wong AD, Kirby J, Guyatt GH, Moayyedi P, Vora P, You JJ. Randomized controlled trial comparing telephone and mail follow-up for recruitment of participants into a clinical trial of colorectal cancer screening. Trials. 2013;14:40.
246. Wu H, Toti G, Morley KI, Ibrahim Z, Folarin A, Kartoglu I, et al. SemEHR: Surfacing semantic data from clinical notes in electronic health records for tailored care, trial recruitment, and clinical research. The Lancet. 2017;390 (SPEC.ISS 1):S97.
247. Wu H, Toti G, Morley KI, Ibrahim ZM, Folarin A, Jackson R, et al. SemEHR: A general-purpose semantic search system to surface semantic data from clinical notes for tailored care, trial recruitment, and clinical research. J Am Med Inform Assoc. 2018;25(5):530-537.

248. Wu T-C, Sarraj A, Jacobs A, Shen L, Indupuru H, Biscamp D, et al. Telemedicine-guided remote enrollment of patients into an acute stroke trial. Annals of Clinical and Translational Neurology. 2015;2(1):38-42.

249. Ybarra ML, Holtrop JS, Prescott TL, Strong D. Process evaluation of a mHealth program: lessons learned from Stop My Smoking USA, a text messaging-based smoking cessation program for young adults. Patient Educ Couns. 2014;97(2):239-243.

250. Young SD. Social media technologies for HIV prevention study retention among minority men who have sex with men (MSM). Aids Behav. 2014;18(9):1625-1629.

251. Zimmerman LP, Goel S, Sathar S, Gladfelter CE, Onate A, Kane LL, et al. A Novel Patient Recruitment Strategy: Patient Selection Directly from the Community through Linkage to Clinical Data. Appl Clin Inform. 2018;9(1):114-121.
