# Supplementary material for: Digital tools for the recruitment and retention of participants in randomised controlled trials: a systematic map
Source: Trials. 2020 Jun 5;21:478. doi: 10.1186/s13063-020-04358-3 (PMC7273688; doi:10.1186/s13063-020-04358-3)
Supplement: Supplementary file 4 — Additional file 4: Appendix 1. Systematic map protocol [file 13063_2020_4358_MOESM4_ESM.doc]

**A systematic map of digital tools for the recruitment and retention of participants in clinical and health** **studies**

**Final version**

**30 June 2018**

Correspondence to:

Dr Geoff Frampton

Senior Research Fellow

Southampton Health Technology Assessments Centre (SHTAC)
University of Southampton
First Floor, Epsilon House, Enterprise Road
The University of Southampton Science Park
Southampton
SO16 7NS
UK

Tel: +44 (0) 23 80 599299
Fax: +44 (0) 23 80 595662

Email: [gkf1@soton.ac.uk](mailto:gkf1@soton.ac.uk)

1. **Background and rationale**

**1.1 The problem: inadequate patient recruitment and retention**

Recruiting participants to clinical and health studies and then retaining them in the studies is a key determinant of research efficiency, but is highly challenging, and poor rates of recruitment to randomised controlled trials (RCTs) are commonly reported.[^3^](#_ENREF_3) In reviews of clinical trials sponsored by the Medical Research Council (MRC) and/or the Health Technology Assessment (HTA) programme[^4-7^](#_ENREF_4) the proportion of trials achieving their original recruitment target ranged from only 31% (of 114 trials that recruited between 1994 and 2002)[^4^](#_ENREF_4) to 60% (of 151 trials published from 2004 to April 2016).[^7^](#_ENREF_7) Although there appears to have been some improvement over time, the latest data[^7^](#_ENREF_7) suggest that around 40% of trials may still fail to reach their intended enrolment, despite considerable interest in approaches that might improve study recruitment.

Once patients have been recruited into clinical or health studies the rates at which they are retained in the studies can be highly variable, depending on the nature of the population, condition, intervention, comparator and outcomes being studied. For example, estimates from trials in Alzheimer’s disease[^9^](#_ENREF_9) suggest that that there is an average dropout rate of 30% across trials and 85% of trials fail to retain enough patients,[^10^](#_ENREF_10) whilst in a systematic review of 87 randomised controlled trials (RCTs) of asthma inhalers we found that dropout rates ranged from 0% to over 40%.[^11^](#_ENREF_11)

Inadequate recruitment and/or retention of participants in clinical and health studies creates extensive research waste and has several important consequences which collectively impede the registration of new therapies and may mean that policy makers base their decisions on inferior evidence:

- reduced statistical power of studies, increasing the risk of type II error (failure to detect real treatment effects);[^12^](#_ENREF_12)
- substantial waste of patients’ and investigators’ time (e.g. 481 trials that closed during 2011 due to inadequate recruitment had already involved over 48,000 patients[^13^](#_ENREF_13));
- extended study duration (phase 2 to phase 4 clinical studies often end up doubling their original timelines in an attempt to meet their intended enrolment target, which incurs substantial costs to study sponsors[^14^](#_ENREF_14));
- ethical problems: it may not be possible to determine whether an intervention to which participants were exposed before a study closed would do more harm than good;[^2^](#_ENREF_2)
- nonrandom participant dropout can introduce bias and limit the relevance of the study’s findings to the target patient population;[^15^](#_ENREF_15)
- inefficient enrolment restricts patients’ access to studies (e.g. up to 60% of potentially eligible patients may miss being identified[^16^](#_ENREF_16)).

**1.2 Potential solutions: digital tools**

To address inadequate patient recruitment and/or retention in studies, clinical trial units (CTUs), funding agencies and study investigators are increasingly exploring the use of digital tools to identify, recruit and retain study participants. The range of digital tools that could assist with the recruitment and/or retention of study participants is potentially very wide. For example, a systematic review of the use of computers for patient recruitment into clinical trials found 79 different recruitment systems.[^17^](#_ENREF_17) Tools for digital recruitment and/or retention of patients include (among others): telephone messaging, audio messages,[^19^](#_ENREF_19) videos, radio advertisements, television advertisements,[^20^](#_ENREF_20) online advertisements, internet websites and tools, including online surveys, social media, smartphone apps, [^22^](#_ENREF_22) computer pop-up reminders,[^26^](#_ENREF_26) email messages, text messaging, and automated eligibility screening of electronic health records, data warehouses, or other patient data sources. The automated screening approaches may be subdivided according to the different algorithms employed for predicting patient eligibility, which include, among others, machine learning approaches and case-based reasoning models. These digital tools may either be used alone or in various combinations, and may be combined with non-digital approaches. For example, a strategy to improve patient recruitment, enrolment, engagement and retention in a clinical trial of weight loss for students included a smartphone app, television screens, email messaging, text messaging, online advertisements, and social media, as well as printed materials (flyers, coasters, pens, posters, and postcards).[^22^](#_ENREF_22) In another example, an intervention to increase research participation of young female cancer survivors included internet, email, and social media components as well as newspaper advertisements that appeared both online and in print.[^35^](#_ENREF_35)

**1.3 Current evidence**

Although the range of digital tools potentially available to assist with the recruitment and/or retention of patients in clinical and health studies is diverse, few evidence syntheses have investigated the extent to which these tools have been evaluated. Our scoping searches identified several relevant systematic reviews, including two Cochrane Reviews, but these do not fully capture the range of available digital tools and they are not up-to-date:

*Systematic reviews of patient recruitment strategies*

- Treweek et al. (2013, 2018) conducted a Cochrane Review of strategies to improve recruitment to randomised or quasi-randomised trials. They excluded observational studies and studies of interventions to improve patient retention. The search strategy was not specifically tailored to locate digital tools, although some evaluations of digital tools, principally telephone reminders, were identified. The searches (February 2015) are now over 3 years out of date.
- Köpke et al. (2014)[^17^](#_ENREF_17) included computer-based approaches for patient recruitment, not limited by study design. Although stated as being a systematic review, it is not strictly, since only one person conducted eligibility screening. The searches (January 2014) are now over 4 years out of date.
- Fletcher et al. (2012)[^3^](#_ENREF_3) included strategies aimed at improving the patient recruitment activity of clinicians, not limited by study design. However, no digital tools were included and the searches (March 2011) are now over 7 years out of date.
- Caldwell et al. (2010)[^37^](#_ENREF_37) covered a broad array of strategies for increasing patient recruitment, but excluded non-randomised studies. The search strategy was not specifically tailored to locate digital tools, although some studies of information provision to patients using telephone and video were included. The searches (April 2009) are now over 9 years out of date.

*Systematic reviews of patient retention strategies*

- Brueton et al. (2013)[^38^](#_ENREF_38) conducted a Cochrane Review of strategies to improve patient retention in randomised or quasi-randomised trials. They excluded observational studies and studies of interventions to improve patient recruitment. The search strategy was not specifically tailored to locate digital tools, although some evaluations of telephone follow-up were identified. The searches (May 2012) are now over 6 years out of date.
- Booker et al. (2011)[^39^](#_ENREF_39) covered a broad array of strategies for patient retention in population-based cohort studies and RCTs. The search strategy was not specifically tailored to locate digital tools, but several studies of telephone contact interventions were included. The searches (January 2011) are now over 7 years out of date.

**1.4 Rationale for systematic mapping as an evidence synthesis approach**

The Cochrane Review of strategies for improving patient recruitment conducted by Treweek et al. (2018) found that studies have tended to evaluate new interventions that are dissimilar to earlier ones, making quantitative combination of data difficult.[^2^](#_ENREF_2) This also seems to be borne out by the other relevant systematic reviews we identified (section 1.3) which largely reported narrative syntheses of the evidence. The evidence synthesis approach to be employed in the current project should, ideally, be able to capture this diversity of the individual tools and the studies that have evaluated them.

Another consideration when planning the evidence synthesis approach is the varying complexity of the interventions. As noted above (section 1.3), some interventions contained more than one digital tool, and some included both digital and non-digital strategies. A distinction may need to be made between the effectiveness of specific tools and the effectiveness of “bundles” of tools in which the effectiveness of the constituent tools may not be separable.

The definition and classification of digital tools is a further factor to consider when planning the evidence synthesis. Ideally, tools should be defined in such a way that the categories are informative and mutually exclusive, to avoid double counting or inconsistent grouping in analyses. Although our scoping searches have used a tentative initial classification in which we have mentioned smartphone apps, internet and social media (section 1.3), there is potential for overlap (e.g. in cases where the social media is delivered through the smartphone app and/or internet).

The diversity, uniqueness, and in some cases complexity, of strategies for improving patient recruitment and retention suggest that systematic mapping would be an appropriate initial approach to the evidence synthesis, to identify and characterise the range of digital tools for improving patient recruitment and/or retention and characterise the studies that have evaluated these tools. Systematic mapping can:

- provide an overview of the characteristics of the diverse evidence base, which would be difficult in a typical systematic review where the question is usually more specific;
- reveal patterns, such as trends, clusters and links in the evidence base which might help to improve the definitions and classification of digital tools;
- provide a basis for scrutiny and discussion of the evidence base, to assist a systematic prioritisation of particular topic areas where further primary research studies or focused secondary research (e.g. systematic review) would be most valuable and efficient.

Systematic mapping (NB also referred to in the literature as an evidence mapping) begins in the same way as systematic review, based on extensive searches for evidence and systematic screening to determine eligibility of the identified studies, but provides a descriptive output rather than an estimate of effects.[^40^](#_ENREF_40) Systematic maps can be helpful in identifying strengths and weaknesses of a broad and/or heterogeneous evidence base in order to plan and prioritise focused evaluative syntheses, e.g. using one or more subsequent systematic review(s).[^40-47^](#_ENREF_40) Systematic mapping was deemed the most appropriate evidence synthesis approach in the current project for the following reasons:

- no comprehensive overview of the evidence base relating to the use of digital tools for patient recruitment and/or retention currently exists;
- the most appropriate, or highest-priority, topics to focus on with systematic reviews within this evidence base are not fully clear at present;
- systematic mapping can identify strengths and weaknesses in the evidence base to help prioritise further research, including the identification of focused questions for future systematic reviews of high priority to stakeholders (i.e. research sponsors, CTUs, and study investigators).

**1.5 Role of systematic mapping within the project**

The project “User-focused research to identify the benefits of innovative digital recruitment and retention tools for more efficient conduct of randomised trials” consists of three data collection and analysis activities. Systematic mapping, as described in the present protocol, is the third of these activities:

- Phase 1: Scoping searches to identify digital tools for patient recruitment and retention.
- Phase 2: Interviews and focus groups with stakeholders, including research funders, study investigators, CTUs and ethics committees, to identify which digital tools for trial recruitment and retention are currently being used in practice and their perceived strengths and weaknesses.
- Phase 3: Systematic mapping to identify and describe the characteristics of research studies which have evaluated the effectiveness of digital tools for patient recruitment and/or retention.

The systematic mapping (phase 3) will be informed by phases 1 and 2 of the project. A list of digital tools identified in phase 1 will inform development of the search strategy for the systematic map. The names and features of digital tools identified in phase 2, including stakeholders’ perceptions of key strengths and weaknesses of the tools, may be used as keywords in the data coding step of the mapping exercise (for details of the mapping methods see section 4).

1. **Systematic map research question**

The question that will be addressed by the systematic map is “which digital tools have been evaluated for improving patient recruitment into clinical and health studies, or patient retention within the studies?”.

The map question captures both the characteristics of digital tools and characteristics of the research studies that have evaluated them.

1. **Aims and Objectives**

The aim of the current work is to identify and describe the research studies that have evaluated the effectiveness of digital tools for improving the recruitment and/or retention of patients in clinical and health research studies, and to describe the characteristics of those digital tools that have been evaluated.

This mapping exercise has two objectives:

- To develop a systematic map to identify and characterise the research studies that have evaluated digital tools for recruiting and retaining participants in clinical and health studies.
- Based on the results of the map, to summarise any key evidence gaps and areas where a more detailed focused evidence synthesis of effectiveness could be worthwhile; and to provide recommendations for further research as necessary.

1. **Methods**

Standard methods for systematic mapping will be followed, with the main steps comprising literature searching; eligibility screening; keyword coding of eligible studies to develop the map; and presentation and interpretation of the map.

**4.1 Literature Searching**

A comprehensive search for relevant literature will be undertaken by an experienced health information specialist. Search sources will include key health bibliographic databases, relevant internet sites, contact with experts in the field, and checking of reference lists of relevant articles. A draft search strategy has been iteratively developed (Appendix 1) and will be further refined based on feedback received by stakeholders, if appropriate.

The following electronic bibliographic databases (using the following host platforms) will be searched:

- Medline (Ovid)
- Medline In-Process (Ovid)
- Embase (Ovid)
- The Cochrane Library (Cochrane Database of Systematic Reviews; Cochrane Central Register of Controlled Trials (CENTRAL))
- Cinahl (Ebsco)
- Psychinfo (Ebsco)
- Social Science Citation Index (Web of Science)
- Delphis (University of Southampton Library database)
- Inspec database (Institute of Engineering and Technology)

Searches will be run from 1990 to the present day. This is because interest in digital tools for patient recruitment and/or retention started to develop in the late 1980s and relevant studies are unlikely to have been published before the 1990s.

We anticipate that much of the relevant evidence for this topic will be located in the ‘grey literature’ (i.e. not formally published in academic journals). Particular emphasis will therefore be given to searching the Internet. Two types of internet search will be conducted, using Google and Google Scholar search engines:

- internet pages of relevant organisations (e.g. CTUs; CROs; research funders) will be specifically searched to identify studies they may have conducted on patient recruitment and retention digital tools;
- internet searches will be conducted using broad search terms to capture any interventions that may improve patient recruitment and/or retention, and broad search terms to capture digital tools that may assist with the conduct of clinical and health research studies.

The searches will identify international literature, but will be limited to references in the English language. Members of the project’s Advisory Board will be asked to nominate any potentially relevant studies they are aware of. Potentially relevant references identified from the searches will be imported into an Endnote library for storage and removal of duplicates.

***Protocol amendments:*** *Some changes were made to the list of bibliographic sources that were searched, and studies published prior to 2008 were excluded (see Appendix 3).*

**4.2 Eligibility screening**

*Eligibility screening process*

The references identified in searches will be screened against the map eligibility criteria (stated below) independently by two reviewers. In cases of disagreement a third reviewer will be consulted to reach a consensus. Where titles and abstracts are available these will be screened first. Full-text articles will be retrieved for any titles and abstracts which meet the eligibility criteria or have unclear relevance, and also for references that do not have an abstract or summary (e.g. internet pages and reports). All full text articles will be screened by one reviewer and checked by a second. Again, a third reviewer will be consulted in cases of disagreement. The screening process will be reported according to PRISMA guidelines.[^48^](#_ENREF_48)

*Eligibility criteria*

The question being addressed by the systematic map is of a “PICOD” type, where the eligible populations, interventions, comparators, outcomes and study designs are specified as follows (sections 4.2.1 to 4.2.6).

**4.2.1 Populations**

*Inclusion criteria*

Studies featuring one or more of the following population groups will be eligible:

- Health professionals (e.g. doctors, nurses, therapists)
- Researchers and study administration staff (e.g. study investigators, research managers)
- Patients, their carers or the general public
- Healthy volunteers
- *Exclusion criteria*:
- People from disciplines outside of health, medical or clinical research and who are not patients or their carers
- Mixed populations in which not all participants meet the inclusion criteria and outcomes are not separately reported for those who meet the inclusion criteria

**4.2.2 Interventions**

Studies will be included if they report evaluation of one or more *digital approaches* that are used to recruit and/or to encourage retention of participants into *clinical and health studies*. Digital approaches for recruitment of participants could include, among others, methods that: raise awareness of trials; help eligible people to locate trials; identify eligible patients from databases or during clinical consultations; and/or check a person’s eligibility for a trial. Digital approaches to support retention of participants in trials could include, among others, methods that provide trial information and/or reminders for trial participants to provide data or to attend visits or tests. To be classed as a *digital approach*, the method of participant recruitment or retention should include one or more *digital tools*.A *digital tool* is defined, broadly, in this research study as: an internet, software or social media application; computer; smartphone; electronic tablet; or virtual assistant/gadget (e.g. Pillo Robot, Amazon Alexa), to support patient recruitment and/or retention in clinical and health studies. For the purposes of the current systematic map landline telephones were classed as being a digital tool only if the phone messages were delivered by an automated process (excluding voicemail) (i.e. they had potential to improve efficiency of communication in relation to recruitment or retention, such as saving time, relative to standard practice).

Examples of digital approaches and tools (see also section 1.2 above) include:

- Database searches for offline case finding, e.g. via Clinical Practice Research Datalink (CPRD).
- Tools that flag up a patient as eligible for a study during a health care consultation, using the electronic patient record, e.g. point of care trials.
- Study websites or trial directories, social media, email or text message campaigns that publicise a study to potential participants.
- Smart phone apps or text messages for participants as reminders for attending a study assessment visit or for taking medication.

Studies of multi-component digital approaches (e.g. which contain a digital tool in addition to other types of tool/activity) will be eligible.

*Clinical and health studies* are defined in this project as evaluations of interventions to treat a diagnosed health condition, or to promote health. Health is defined in this project in a broad sense to include health care, public health, and health promotion.

**4.2.3 Comparators**

*Inclusion criteria*

Any comparator is eligible, for example:

- Standard practice for patient recruitment or retention (for the given study sponsor or institution conducting the study);
- Non-digital approaches (e.g. approaches that comprise paper-based or manual tools);
- Recruitment or retention approaches comprising digital tools other than those included in the intervention;
- Digital approaches comprising “bundles” of tools (i.e. where the comparator includes more than one digital and/or non-digital tool).

*Exclusion criteria*

Studies in which the configuration of the comparator and intervention is such that effects of digital tools cannot be separated from effects of non-digital tools will be excluded (e.g. where the intervention and comparator both contain digital and non-digital strategies but the digital strategies are identical and therefore their effects would ‘cancel out’).

**4.2.4 Outcomes**

Studies that report one or more of the following outcome measures will be eligible for inclusion:

- Recruitment rate (e.g. the proportion of the intended number of participants enrolled in the study)
- Quantitative assessment of recruitment accuracy (e.g. the proportion of participants included in a study accurately meeting study inclusion criteria, as assessed by sensitivity, specificity and/or area under the curve estimates).
- Participant retention in a study (e.g. the proportion of recruited participants who remained in the study at the end).

**4.2.5 Study designs**

Although the overall project title refers to randomised trials, the systematic mapping will not be limited by study design, since there is a need to improve the recruitment and retention of patients across clinical and health studies in general, not limited to RCTs.

Any primary evaluation study design is eligible for inclusion (e.g. randomised controlled trial; quasi-experimental study; observational study). The results of the qualitative research and stakeholder survey (phase 2) will identify what level of evidence (e.g. experimental or observational) stakeholders think is adequate enough for them to use digital tools. This inclusion criterion may therefore be revised accordingly.

Any systematic reviews identified from the literature search will be used only as a source of potentially eligible references.

***Protocol amendments (see Appendix 3):***

- *Studies were limited to those in which the target trial was an RCT (or if not explicitly reported as such, appeared likely to have been an RCT).*
- *Standard meeting abstracts were excluded, but extended meeting abstracts were eligible for inclusion if they met all other eligibility criteria.*

**4.3 Coding of eligible studies and development of the systematic map**

All studies meeting the inclusion criteria described above in Section 4.2 will be classified through the systematic application of pre-specified keywords. A draft list of descriptive keywords will be developed (for the main topics to be covered see Appendix 2). This will be partly informed by results from phase 2 of the project (stakeholder survey and focus groups) which will provide information on the types of digital tool that stakeholders use or are aware of; the features of the tools they consider important; relevant outcome measures for evaluating the effectiveness of tools; and the kind of research evidence stakeholders consider adequate for evaluating the effectiveness of tools.

The development of the keywords will also be based on a pilot analysis of a subset of the included studies, to capture information about relevant study characteristics (e.g. their scope and methodology). A logic model devised for this project will also be used as a framework for the development of the keywords. Where appropriate, alternative ways of classifying digital tools as reported in the literature (e.g. by Weng et al.[^16^](#_ENREF_16)) will be considered.

The keywords would cover the following study features (see also Appendix 2):

- the digital tool(s) studied (e.g. purpose of the tool; medium; content; developer/provider, etc)
- study population (e.g. study investigators, researchers, patients)
- study design (e.g. experimental, observational)
- study context (e.g. country; type of research study; health topic area)
- study outcome measures (e.g. proportion of relevant patients recruited, proportion of patients retained).

The keyword coding will not characterise the results of studies.

The draft keyword list will be updated in light of any feedback from the project Advisory Board or other stakeholders. Each study will be coded by one reviewer and, during pilot-testing of the coding process, a random sample of included studies (minimum 20%) will be checked by a second reviewer. If errors or disagreements are identified during pilot-testing these will be discussed by the project team and, if necessary, the coding process may be refined. If refinements to coding are required these will be applied to all included studies to minimise the risk of bias. Once finalised, the keywords will be applied systematically to code all studies that meet the eligibility criteria.

**4.4 Presentation and interpretation of the map**

The list of keywords (and where appropriate any free text descriptors – see Appendix 2) produced for each study will be collated systematically in a Microsoft Excel relational database. Chart and pivot table options within the database will be used to produce a descriptive map of the characteristics of the evidence which will be presented in a final project report using tables, figures and text. A copy of the final database will be made available alongside the final report.

1. **Declaration of interests**

The members of the project team declare no competing interests.

1. **Protocol amendments**

Any amendments required to the protocol will be discussed by the project team. If necessary, an amended protocol will be produced, with a clear statement of the changes made and the rationale for them. The final report will include a section reporting any changes made to the protocol.

***Protocol amendments:*** *As noted above, some protocol amendments were made in relation to searches (section 4.1) and eligibility screening (section 4.2). These are listed, with a rationale, in Appendix 3. All amendments were agreed by the full project team and the project Advisory Board and were applied to all studies included in the final systematic map. Details of the full project team and Advisory Board have been added below (section 9).*

1. **Dissemination**

The key outputs of the project will include:

- A final project report
- A peer-reviewed publication describing the systematic map
- A copy of the Excel map database, provided alongside both the final report and map publication;
- Where possible, the work will be disseminated at relevant conferences, and local meetings

1. **Project Management**

As noted above, this systematic mapping exercise is the third phase of a project entitled “User-focused research to identify the benefits of innovative digital recruitment and retention tools for more efficient conduct of randomised trials”. Management of the current work will therefore be consistent with management of the overall project.

The systematic map will be produced by SHTAC, following SHTAC’s standard project management processes, and will follow a similar approach to the mapping part of a recent evidence synthesis project.[^45^](#_ENREF_45) All electronic project documents will be stored, and backed up, in a dedicated project folder on SHTAC’s J-drive. Regular face-to-face team meetings will be held to plan work, monitor progress, and identify any emergent issues. Progress will be reported back to the overall project team at monthly meetings.

**9. Project Team and Advisory Board**

Systematic map project team (project Phase 3):

- Dr Geoff Frampton (SHTAC, University of Southampton)
- Dr Jonathan Shepherd (SHTAC, University of Southampton)
- Dr Karen Pickett (SHTAC, University of Southampton)
- Mrs Karen Welch (information specialist) (SHTAC, University of Southampton)
- Professor Jeremy Wyatt (Director, Wessex Institute, University of Southampton)

Full project team (Project Phases 1 and 2):

- Professor Gareth Griffiths (Director, Southampton Clinical Trials Unit
- Dr Amanda Blatch-Jones (NETSCC, University of Southampton)
- Dr Jeremy Hinks (NETSCC, University of Southampton)
- Dr Athene Lane (Director, Bristol Randomised Trials Collaboration, University of Bristol)
- Jacqui Nuttall (Southampton Clinical Trials Unit)
- Dr Louise Worswick (NETSCC, University of Southampton)

Advisory Board:

- Dr Andrew Cook (NETSCC and Southampton Clinical Trials Unit)
- Dr Stephen Falk (Bristol and West Clinical Research Network)
- Ms Helen George (Patient and Public Involvement representative)
- Professor Mark Mullee (NIHR Research and Development Service South Central)
- Professor Robert Peveler (Chair, Wessex Clinical Research Network)
- Mr Neil Tape (University Hospital Southampton NHS Foundation Trust)
- Dr Karen Underwood (University Hospital Southampton NHS Foundation Trust)

**10. Project timetable**

The project timetable as tabulated here is consistent with the project milestones as specified in the GANTT chart for the overall project (March 2018 version). If any variation to these timelines is deemed necessary, this will be discussed by the overall project team and reflected in the GANTT chart for the overall project.

| **Activity** | **Date** |
| --- | --- |
| Refine and finalise protocol, including scoping searches and testing of search strategy | March – June, 2018 |
| Literature searches | June – July, 2018 |
| Eligibility screening | June – August, 2018 |
| Application of keywords | July – September, 2018 |
| Completion and circulation of map | October – November, 2018 |
| Write up of project report and publication | October – December, 2018 |
| Submission of final project report and publication | December, 2018 |

**11. References**

**Appendix 1. Medline draft search strategy**

Database: Ovid MEDLINE(R) Epub Ahead of Print, In-Process & Other Non-Indexed Citations, Ovid MEDLINE(R) Daily and Ovid MEDLINE(R) <1946 to Present>

Searched on 23/05/2018

Search Strategy:

1 Patient Selection/ or Patient Participation/ or Informed Consent/ or Research Subjects/ or Eligibility Determination/ (119373)

2 (recruit* or enrol* or accru* or retention).tw,kw. (733231)

3 1 and 2 (10694)

4 (select* adj3 (patient? or participant? or participat*)).tw. (132507)

5 ((retain* or retention) adj3 (patient? or participant? or participat*)).tw. (8189)

6 (recruit* adj3 (patient? or participant? or participat*)).tw. (45585)

7 (recruitment adj3 (toolkit or tool? or strategy or strategies or goal* or challenge* or eligibility)).tw,kw. (3538)

8 ("willingness to participate" or "willing participant*" or "participant rate*").tw,kw. (1662)

9 ("patient recruitment" or "patient identification" or "patient retention" or "patient accrual" or "patient enrolment" or "study enrolment" or "trial enrolment" or "patient eligibility" or "eligibility screening" or "patient? matching" or "matching patient?" or "trial participant?").tw,kw. (8003)

10 ("research subject selection" or "selection of subjects").tw,kw. (392)

11 ("recruitment method*" or "recruitment process*").tw,kw. (1473)

12 (recruit* adj3 (retain or retention)).tw. (4676)

13 (engag* adj3 (retain or retention)).tw. (461)

14 Patient Dropouts/ (7661)

15 (attrition or withdraw* or dropout).tw. (131215)

16 ("refusal to participate" or "unwilling to participate" or "non participation" or "lost to follow up").tw. (16964)

17 ("response rate*" or "non-response rate").tw. (98988)

18 or/4-17 (442257)

19 3 or 18 (448283)

20 (digital adj3 (tool* or solution? or recruit* or identification or invit* or retention or platform? or prescreen* or "pre-screen*" or technolog* or dashboard? or portal)).tw,kw. (3391)

21 ("database tool?" or "interactive tool?" or "software tool?" or "internet tool" or "internet intervention" or "electronic tool?" or "electronic device?" or "computer* assisted intervention*").tw. (12157)

22 (digitiz* or digitis* or digitali*).tw,kw. (25714)

23 Software/ or software.tw,kw. (200041)

24 digital*.ab. /freq=2 (24693)

25 "recruitment portal?".tw,kw. (1)

26 "patient portal?".tw,kw. (625)

27 ("electronic consent" or "e-consent").tw,kw. (35)

28 ("electronic screen*" or "e-screen*" or "electronic data capture" or "EHR data capture").tw,kw. (819)

29 ("e-technology" or "electronic technology" or "e-clinical").tw,kw. (294)

30 "web portal?".tw,kw. (714)

31 (recruitment adj (portal? or database?)).tw,kw. (15)

32 ("virtual study" or "virtual trial" or "virtual clinical trial" or "virtual clinical study").tw. (79)

33 ("remote consent" or "online consent").tw. (22)

34 Remote Consultation/ (4389)

35 (ipad or PDA or "personal digital assistant" or "mobile phone?" or "smart phone?" or smartphone? or "mobile app*" or "mobile technolog*" or "mobile health" or "mobile media" or "health app" or "m health" or palmtop? or laptop? or "hand held device?" or "text messag*" or SMS or IVR or "interative voice recognition" or "voice activation" or "web deliv*").tw. (34132)

36 ("social media" or facebook or twitter or WhatsApp or webchat or crowdsourcing).tw. (8667)

37 (interactive and website?).tw. (861)

38 ("web based" or "web tool?" or "web delivery" or "web delivered" or podcast*).tw. (25946)

39 ("electronic data capture" or "electronic recruitment" or "e-recruit*" or "electronic screening").tw. (514)

40 ("e-mail" or email or "electronic mail*").tw. (11149)

41 ("wearable device*" or "google x wristband").tw,kw. (1156)

42 (telephone adj2 intervention*).tw. (941)

43 Information Systems/ or Hospital Information Systems/ or Medical Records Systems, Computerized/ or Online Systems/ or Medical Informatics/ or Reminder Systems/ or Electronic Mail/ or Decision Support System/ (66438)

44 Bioinformatics/ (60347)

45 Decision Making,Computer Assisted/ (2657)

46 Automatic Data Processing/ or Cloud Computing/ or Information Storage/ (31524)

47 Smartphone/ or cellphone/ or computer, handheld/ or computing methodologies/ or computer systems/ (18655)

48 (automate? or automation).tw. (109656)

49 ("smart patients" or "smart participants").tw,kw. (21)

50 text messaging/ (1930)

51 Electronic Health Records/ (14674)

52 Mobile Applications/ (3022)

53 ("online forum?" or "online interaction?").tw. (506)

54 social media/ or social networking/ (6312)

55 *internet/ (34143)

56 communications media/ (1406)

57 videoconferencing/ (1245)

58 (video* or audiovisual).tw. (108040)

59 "big data".tw. (3501)

60 Telehealth/ or Telemedicine/ (17573)

61 ORRCA.tw. (2)

62 (CTTI or "clinical trials transformation initiative" or ORRCA or "online resource for recruitment research in clinical trials").tw,kw. (37)

63 "QuinteT Recruitment Intervention".tw,kw. (6)

64 "clinical trial educator program".tw,kw. (1)

65 ("Q-QAT" or "quanti-qualitative appointment training").tw,kw. (1)

66 "EHR4CR".tw,kw. (23)

67 transcelerate.tw,kw. (5)

68 "fox trial finder".af. (3)

69 "patients like me".tw,kw. (4)

70 eSource.af. (10)

71 mHealth.af. (2872)

72 "network oriented research assistant".af. (1)

73 "Research Kit".tw,kw. (22)

74 "Trial Forge".tw,kw. (2)

75 or/20-74 (688453)

76 qualitative research/ or social validity, research/ (39382)

77 Public Health/ (73303)

78 Epidemiologic Methods/ (30795)

79 validation studies/ (89575)

80 Validation Studies as Topic/ (1898)

81 Clinical Studies as Topic/ (251)

82 Clinical Trials as Topic/ (183855)

83 Randomized Controlled Trials as Topic/ (116645)

84 exp Clinical Study/ (848285)

85 (trial? or study or studies or research).tw. (9331369)

86 or/76-85 (9686042)

87 19 and 75 and 86 (14532)

88 limit 87 to english language (13995)

89 (recruit* or retention).ti. (52317)

90 (recruit* or enrol* or retention or accru*).ab. /freq=2 (137292)

91 89 or 90 (167479)

92 88 and 91 (2253)

93 limit 88 to "reviews (maximizes specificity)" (579)

94 limit 88 to "qualitative (maximizes specificity)" (1069)

95 19 and 75 (18801)

96 limit 95 to evaluation studies (584)

97 92 or 93 or 94 or 96 (4074)

**Appendix 2.** Summary of the main keyword groups that will be used to code studies in the systematic map

| **Keyword group** | **Comments** |
| --- | --- |
| A standard set of keywords to elicit basic characteristics of the studies, including date, location, publication source(s), etc. | See for example Shepherd et al. (2018) [^45^](#_ENREF_45) |
| Keywords describing the population | Keywords may be supported with free text descriptions to ensure all relevant populations are precisely captured. |
| Keywords describing the intervention | The project team will agree on a final classification of digital tools (which may possibly differ from that suggested in the current protocol), developed iteratively from pilot-testing the mapping process. |
| Keywords describing the comparator | Keywords should distinguish various possible permutations of digital/non-digital tools that may be used together within an intervention and/or comparator, and distinguish between single tools/bundles of tools. |
| Keywords describing the outcome(s) | Keywords may be supported with free text descriptions to ensure all relevant outcomes are precisely captured. |
| Keywords describing the study design | Designs will be classified as RCTs, non-randomised controlled trials, case-control studies), and single/multi-cohort before-after comparisons (among others). The final list of designs will emerge iteratively from pilot testing of the mapping process. |
| Keywords describing other aspects of the evidence base | Additional aspects of study conduct or digital tool use as identified in phase 1 and phase 2 of the project, and as suggested by the project Advisory Board, may be captured by keywords. |

**Appendix 3. Protocol amendments**

The following changes to the protocol were made during July-December 2018 after the final version of the protocol had been agreed by the full project team and project Advisory Board (for details of the team and Advisory Board see section 9). The rationale for each change is tabulated below. All changes were discussed and agreed with the full project team and Advisory Board and were applied retrospectively to all included studies in the systematic map to minimise the risk of selection bias.

| **Protocol section** | **Item** | **Change made** | **Justification** |
| --- | --- | --- | --- |
| **4.1** | The protocol lists a range of sources that would be searched. | We did not specifically search The Cochrane Library, Cinahl, Psychinfo, or Delphis. However, we did search the ORRCA database (Online Resource for Recruitment Research in Clinical Trials) ([http://www.orrca.org.uk](http://www.orrca.org.uk/)) which brings together published and ongoing work in the field of clinical trial recruitment research. | We searched a smaller range of sources to improve efficiency, given the large overlap between sources, to ensure we could meet the project timescale. The ORRCA searches, and checks of reference lists of the included studies, identified numerous potentially relevant studies for the current systematic map, but none that had not also been identified in our MEDLINE, Embase, Inspec, Web of Science and internet searches. |
| **4.1** | The protocol states that internet pages of relevant organisations (e.g. CTUs; CROs; research funders) would be searched. | We did not specifically search the pages of CROs, CTUs and funding agencies. | (i) The full project (i.e. including Phase 1 and Phase 2) involved a survey of digital tools used by CTUs and funding agencies and relevant references were provided by personal communication. (ii) We found it inefficient to search specific CTU and CRO web pages due to the heterogeneity of websites. Instead we included “CTU” and “CRO” as search terms in broad searches in Google and Google Scholar. |
| **4.1** | The protocol states that reference lists of relevant articles would be checked. | We checked the reference lists of all included studies published after 2014 (n=45, i.e. 45% of all studies in the map). | Checking the reference lists of all studies included in the map was not feasible within the project timescale. Checks of the reference lists published after 2014 did not identify any potentially relevant new studies that we had not already identified in our searches. We therefore considered the risk of missing any important studies by not checking the reference lists of the remaining studies to be low. |
| **4.2** | The eligibility screening process does not mention whether meeting abstracts would be eligible for inclusion in the map. | Meeting abstracts that passed title and abstract screening were subsequently excluded. | Meeting abstracts that were included in the full-text screening step generally (i) contained insufficient data to be informative, (ii) were time consuming to check for any linked full-text articles, and (iii) in most cases no linked full-text articles were found (where linked full-text articles did exist these were also identified by our database searches). The impact of excluding meeting abstracts is likely to be negligible given the limited data they could contribute. NB “extended” meeting abstracts that contained additional information such as in Tables or Charts were treated separately as though they were full text documents and were included in the systematic map if they met the eligibility criteria. |
| **4.1** | The initial search process specified a date limit of 1990. | At the full-text screening step, studies published before 2008 were excluded. | After completing approximately half the full-text screening (after excluding meeting abstracts as explained above), we estimated that the systematic map would include approximately 175 studies, each of which would require scrutiny, coding and checking. This would not have been feasible within the available resources and timescale. We estimated that limiting the map to studies published from 2008 onwards and limiting to studies whose target trials were RCTs would result in a map containing approximately 100 studies. We considered this feasible to complete within the required timescale and the Advisory Board agreed that such a map would be relevant to stakeholders. |
| **4.2.5** | The initial eligibility criteria permitted a broad range of target study designs to be included. | At the full-text screening step, eligible studies were limited to those whose target trial was an RCT. |
