# Supplementary material for: Digital tools for the recruitment and retention of participants in randomised controlled trials: a systematic map
Source: Trials. 2020 Jun 5;21:478. doi: 10.1186/s13063-020-04358-3 (PMC7273688; doi:10.1186/s13063-020-04358-3)
Supplement: Supplementary file 5 — Additional file 5: Appendix 2. Search strategies. [file 13063_2020_4358_MOESM5_ESM.docx]

**Appendix 2 Search strategies**

| Database Details | Literature Search Strategies | Results |
| --- | --- | --- |
| Database: Ovid MEDLINE(R) Epub Ahead of Print, In-Process & Other Non-Indexed Citations, Ovid MEDLINE(R) Daily and Ovid MEDLINE(R) <1946 to Present>  Searched: 17/07/2018 | Database: Ovid MEDLINE(R) Epub Ahead of Print, In-Process & Other Non-Indexed Citations, Ovid MEDLINE(R) Daily and Ovid MEDLINE(R) <1946 to Present>  Search Strategy:  1 Patient Selection/ or Patient Participation/ or Informed Consent/ or Research Subjects/ or Eligibility Determination/ (119715)  2 (recruit* or enrol* or accru* or retention).tw,kw. (738259)  3 1 and 2 (10727)  4 (select* adj3 (patient? or participant? or participat*)).tw. (133230)  5 ((retain* or retention) adj3 (patient? or participant? or participat*)).tw. (8212)  6 (recruit* adj3 (patient? or participant? or participat*)).tw. (45958)  7 (recruitment adj3 (toolkit or tool? or strategy or strategies or goal* or challenge* or eligibility)).tw,kw. (3563)  8 ("willingness to participate" or "willing participant*" or "participant rate*").tw,kw. (1677)  9 ("patient recruitment" or "patient identification" or "patient retention" or "patient accrual" or "patient enrolment" or "study enrolment" or "trial enrolment" or "patient eligibility" or "eligibility screening" or "patient? matching" or "matching patient?" or "trial participant?").tw,kw. (8019)  10 ("research subject selection" or "selection of subjects").tw,kw. (392)  11 ("recruitment method*" or "recruitment process*").tw,kw. (1487)  12 (recruit* adj3 (retain or retention)).tw. (4712)  13 (engag* adj3 (retain or retention)).tw. (460)  14 Patient Dropouts/ (7672)  15 (attrition or withdraw* or dropout).tw. (131836)  16 ("refusal to participate" or "unwilling to participate" or "non participation" or "lost to follow up").tw. (17047)  17 ("response rate*" or "non-response rate").tw. (99466)  18 or/4-13 (201170)  19 3 or 18 (207680)  20 or/14-17 (251388)  21 2 and 20 (24838)  22 19 or 21 (228239)  23 (digital adj3 (tool* or solution? or recruit* or identification or invit* or retention or platform? or prescreen* or "pre-screen*" or technolog* or dashboard? or portal)).tw,kw. (3459)  24 ("database tool?" or "interactive tool?" or "software tool?" or "internet tool" or "internet intervention" or "electronic tool?" or "electronic device?" or "computer* assisted intervention*").tw. (12294)  25 (digitiz* or digitis* or digitali*).tw,kw. (25812)  26 Software/ or software.tw,kw. (201452)  27 digital*.ab. /freq=2 (24902)  28 "recruitment portal?".tw,kw. (1)  29 "patient portal?".tw,kw. (623)  30 ("electronic consent" or "e-consent").tw,kw. (36)  31 ("electronic screen*" or "e-screen*" or "electronic data capture" or "EHR data capture").tw,kw. (828)  32 ("e-technology" or "electronic technology" or "e-clinical").tw,kw. (293)  33 "web portal?".tw,kw. (714)  34 (recruitment adj (portal? or database?)).tw,kw. (14)  35 ("virtual study" or "virtual trial" or "virtual clinical trial" or "virtual clinical study").tw. (79)  36 ("remote consent" or "online consent").tw. (22)  37 Remote Consultation/ (4396)  38 (ipad or PDA or "personal digital assistant" or "mobile phone?" or "smart phone?" or smartphone? or "mobile app*" or "mobile technolog*" or "mobile health" or "mobile media" or "health app" or "m health" or palmtop? or laptop? or "hand held device?" or "text messag*" or SMS or IVR or "interactive voice recognition" or "voice activation" or "web deliv*").tw. (34578)  39 ("social media" or facebook or twitter or WhatsApp or webchat or crowdsourcing).tw. (8854)  40 (interactive and website?).tw. (864)  41 ("web based" or "web tool?" or "web delivery" or "web delivered" or podcast*).tw. (26021)  42 ("electronic data capture" or "electronic recruitment" or "e-recruit*" or "electronic screening").tw. (521)  43 ("e-mail" or email or "electronic mail*").tw. (11201)  44 ("wearable device*" or "google x wristband").tw,kw. (1199)  45 (telephone adj2 intervention*).tw. (946)  46 Information Systems/ or Hospital Information Systems/ or Medical Records Systems, Computerized/ or Online Systems/ or Medical Informatics/ or Reminder Systems/ or Electronic Mail/ or Decision Support System/ (66567)  47 Bioinformatics/ (60691)  48 Decision Making,Computer Assisted/ (2654)  49 Automatic Data Processing/ or Cloud Computing/ or Information Storage/ (31556)  50 Smartphone/ or cellphone/ or computer, handheld/ or computing methodologies/ or computer systems/ (18825)  51 (automate? or automation).tw. (110174)  52 ("smart patients" or "smart participants").tw,kw. (21)  53 text messaging/ (1932)  54 Electronic Health Records/ (14763)  55 Mobile Applications/ (3139)  56 ("online forum?" or "online interaction?").tw. (506)  57 social media/ or social networking/ (6387)  58 *internet/ (34155)  59 communications media/ (1407)  60 videoconferencing/ (1251)  61 (video* or audiovisual).tw. (108708)  62 "big data".tw. (3600)  63 Telehealth/ or Telemedicine/ (17664)  64 ("Mechanical Turk" or MTurk).tw. (507)  65 (CTTI or "clinical trials transformation initiative" or ORRCA or "online resource for recruitment research in clinical trials").tw,kw. (36)  66 "QuinteT Recruitment Intervention".tw,kw. (6)  67 "clinical trial educator program".tw,kw. (1)  68 ("Q-QAT" or "quanti-qualitative appointment training").tw,kw. (1)  69 "EHR4CR".tw,kw. (23)  70 transcelerate.tw,kw. (6)  71 "fox trial finder".af. (3)  72 "patients like me".tw,kw. (4)  73 eSource.af. (9)  74 mHealth.af. (2970)  75 "network oriented research assistant".af. (1)  76 "Research Kit".tw,kw. (23)  77 "Trial Forge".tw,kw. (2)  78 or/23-77 (693184)  79 22 and 78 (11622)  80 qualitative research/ or social validity, research/ (39784)  81 Public Health/ (73370)  82 Epidemiologic Methods/ (30799)  83 validation studies/ (89969)  84 Validation Studies as Topic/ (1900)  85 Clinical Studies as Topic/ (256)  86 Clinical Trials as Topic/ (184070)  87 Randomized Controlled Trials as Topic/ (117088)  88 "non-randomi?ed".ab. (9932)  89 exp Clinical Study/ (851463)  90 (trial? or study or studies or research).tw. (9383763)  91 Evaluation Studies/ (235759)  92 or/80-91 (9833461)  93 79 and 92 (9475)  94 (recruit* or enrol* or retention or retain or accru*).ab. /freq=2 (141933)  95 ((recruit* or enrol* or retention or retain or accru*) and (trial* or study or studies or research)).ti. (7456)  96 94 or 95 (145133)  97 93 and 96 (2294)  98 (recruit* and participant*).tw. (56711)  99 (tool* or digital).tw,kw. (695853)  100 (recruit* and (retain or retention)).tw. (8201)  101 or/98-100 (754796)  102 93 and 101 (4149)  103 97 or 102 (4904)  104 "urinary retention".tw. (7967)  105 103 not 104 (4895)  106 "Secondary use of routinely collected patient data in a clinical trial".ti. (1)  107 "Using Mechanical Turk for research on cancer survivors".ti. (1)  108 22 and 64 (89)  109 105 or 106 or 107 or 108 (4907)  110 limit 109 to english language (4836) | 4836 |
|  |  |  |
| Embase Ovid  1974-18/07/2018  Searched 17/07/2018 | 1 (recruit* adj5 ("social media" or facebook or twitter or WhatsApp or webchat or crowdsourc*)).tw. (561)  2 (recruit* adj5 (electronic* or web or portal* or "smart patient*" or smartphone* or virtual* or online or platform* or internet)).tw. (3434)  3 (recruit* adj5 (ipad or "personal digital assistant" or "mobile app*" or "mobile technology" or "health app*")).tw. (27)  4 (recruit* adj5 ("text messag*" or SMS or "interactive voice recognition" or "voice activation" or podcast*)).tw. (43)  5 (recruit* adj5 ("electronic health record*" or "electronic medical record*" or EHR)).tw. (92)  6 Electronic Health Record/ and recruit*.tw. (187)  7 (recruit* and "interactive messag*").tw. (2)  8 "automated recruitment".tw. (7)  9 (recruitment adj automation).tw. (2)  10 or/1-9 (4103)  11 (recruit* and (trial or study or studies or research)).ti. (3622)  12 ("social media" or facebook or twitter or WhatsApp or webchat or crowdsourc* or electronic* or web or portal* or "smart patient*" or smartphone* or virtual* or online or platform* or internet or ipad or "personal digital assistant" or "mobile app*" or "mobile technology" or "health app*" or "text messag*" or SMS or "interactive voice recognition" or "voice activation" or podcast* or "interactive messag*" or automated or automation or EHR).tw. (991415)  13 11 and 12 (535)  14 10 or 13 (4409)  15 (recruit* adj5 (retain or retention or attrition or dropout*)).tw. (6139)  16 (recruit* adj5 ("response rate" or "nonresponse rate" or "non response rate")).tw. (281)  17 ("retention rate" or "attrition rate" or "dropout rate" or "lost to follow up").tw. (35575)  18 patient centric*.tw. (1096)  19 patient dropout/ (656)  20 or/15-19 (43384)  21 12 and 20 (3134)  22 limit 21 to clinical trial (270)  23 "clinical trial (topic)"/ (94460)  24 21 and 23 (89)  25 e-recruit*.tw. (27)  26 14 or 22 or 24 or 25 (4732)  27 limit 26 to (human and english language) (4221) | 4221 |
|  |  |  |
| Inspec  All Years  Searched 18/07 | Indexes=Inspec Timespan=All years & Language English  # 21 298 #20 OR #19 OR #18 OR #17 OR #16 OR #15 OR #14 OR #13 OR #12 OR #11 OR #10 OR #9 OR #8 OR #7 OR #6 OR #5 OR #4 OR #3 OR #2 OR #1  # 20 4 (TS=((method* or system* or module* or "data process*" or technolog* or automatic* or electronic*) and (recruit* and retention and "clinical trial*"))) AND LANGUAGE: (English)  # 19 61 (TS=((method* or system* or module* or "data process*" or technolog* or automatic* or electronic*) and ("patient recruitment" or "patient accrual" or "patient retention" or "patient dropout*"))) AND LANGUAGE: (English)  # 18 11 (TS=((recruit* and "clinical trial*") and (telehealth or telemedicine))) AND LANGUAGE: (English)  # 17 7 (TS=(recruit* and "clinical trial*" and video*)) AND LANGUAGE: (English)  # 16 12 (TS=(recruit* and "clinical trial*" and ontolog*)) AND LANGUAGE: (English)  # 15 13 (TS=((recruit* and "clinical trial*") and (warehouse or architecture))) AND LANGUAGE: (English)  # 14 5 (TS=(EHR4CR)) AND LANGUAGE: (English)  # 13 6 (TS=("decision support system*" and recruit* and "clinical trial*")) AND LANGUAGE: (English)  # 12 2 (TS=("cloud computing" and recruit* and clinical stud*)) AND LANGUAGE: (English)  # 11 1 (TS=(recruit* and "clinical trial*" and cloud)) AND LANGUAGE: (English)  # 10 8 (TS=((recruit* and "clinical trial*") and ("data warehouse*" or "data mining" or "data mine"))) AND LANGUAGE: (English)  # 9 4 (TS=((recruit* and "clinical trial*") and ( module* or dashboard*))) AND LANGUAGE: (English)  # 8 20 (TS=(recruit* and tool* and "clinical trial*")) AND LANGUAGE: (English)  # 7 42 (TS=(("electronic health record*" NEAR study) and (recruit* or selection or participat* or participant* or eligibil*))) AND LANGUAGE: (English)  # 6 38 ((TS=(("electronic health record*" NEAR research) and (recruit* or selection or participat* or participant* or eligibil*)))) AND LANGUAGE: (English)  # 5 43 ((TS=(("electronic health record*" and trial*) and (recruit* or selection or participat* or participant* or eligibil*)))) AND LANGUAGE: (English)  # 4 81 ((TS=((recruit* and "clinical trial*") and (automation or automated or electronic or database or web or portal or platform or software or technolog*)))) AND LANGUAGE: (English)  # 3 1 (TS=((recruit* and patient*) and ("voice recognition" or "voice activation" or podcast))) AND LANGUAGE: (English)  # 2 92 (TS=(enrol* NEAR ("clinical trial*" or "clinical study" or "clinical studies"))) AND LANGUAGE: (English)  # 1 4 (TS=(recruit* and retention* and ("clinical trial*" or "clinical study" or "clinical studies"))) AND LANGUAGE: (English) | 298 |
|  |  |  |
| Web of Science cross database search: 2000-2018  WOS Core Collection includes:  Biosis Citation Index  Biosis Previews  Current Contents Connect  Data Citation Index  Derwent Innovations Index  Inspec  KCI Korean Journal Database  18/07/2018 | # 25 1,060 #24 OR #23 OR #22 OR #21 OR #20 OR #19 OR #18 OR #17 OR #16 OR #15 OR #14 OR #13 OR #12 OR #11 OR #10 OR #9 OR #8 OR #7 OR #6 OR #5 OR #4 OR #3 OR #2 OR #1 Search language=English Timespan=2000-2018  # 24 15 TS=("e-technolog*" and trial*)  # 23 5 TS=(digital tool* and recruitment and retention and trial*)  # 22 34 TS=((digital and "clinical trial*") and ("patient recruitment" or "patient accrual" or "patient retention"))  # 21 27 TS=((electronic* NEAR "clinical trial*") and ("patient recruitment" or "patient accrual" or "patient retention" or "patient dropout*"))  # 20 172 TS=((recruit* NEAR "clinical trial*") and ("social media" or facebook or twitter or WhatsApp or webchat or crowdsourc* or "web-based" or portal* or "smart patient*" or smartphone* or virtual* or ipad or "personal digital assistant" or "mobile app*" or "mobile technology" or "health app*" or "text messag*" or SMS or "interactive voice recognition" or "voice activation" or podcast* or "interactive messag*"))  # 19 10 TS=(("health informatics" and recruit*) and ("clinical trial*" or "clinical study" or "clinical studies" or "clinical research"))  # 18 117 TS=((module* or "data process*" or technolog* or automatic* or electronic* or digital) and (recruit* and retention and "clinical trial*"))  # 17 180 TS=((recruit* and "clinical trial*") and (telehealth or telemedicine))  # 16 49 TS=((recruit* NEAR "clinical trial*") and (video*))  # 15 28 TS=(recruit* and "clinical trial*" and ontolog*)  # 14 64 TS=((recruit* and "clinical trial*") and (warehouse or architecture))  # 13 41 TS=("decision support system*" and recruit* and "clinical trial*")  # 12 7 TS=("cloud computing" and recruit* and clinical stud*)  # 11 8 TS=(recruit* and "clinical trial*" and cloud)  # 10 57 TS=((recruit* and "clinical trial*") and ("data warehouse*" or "data mining" or "data mine"))  # 9 22 TS=((recruit* NEAR "clinical trial*") and( module* or dashboard*))  # 8 40 TS=(("clinical trial*" NEAR recruit*) and ("digital tool*" or "electronic tool*" or database tool* or software tool*))  # 7 11 TS=(("clinical trial*" and recruit* and retention) and ("digital tool*" or "electronic tool*" or database tool* or software tool*))  # 6 18 TS=((software NEAR recruitment) and "clinical trial*")  # 5 6 TS=("automated recruit*" and "clinical trial*")  # 4 265 TS=(("electronic health record*" NEAR research) and (recruit* or selection or participat* or participant* or eligibil*))  # 3 108 TS=(("electronic health record*" NEAR trial*) and (recruit* or selection or participat* or participant* or eligibil*))  # 2 6 TS=((recruit* and "clinical trial*") and ("voice recognition" or "voice activation" or podcast))  # 1 14 TS=((recruit* and patient*) and ("voice recognition" or "voice activation" or podcast)) | 1060 |
