# Supplementary material for: Digital tools for the recruitment and retention of participants in randomised controlled trials: a systematic map
Source: Trials. 2020 Jun 5;21:478. doi: 10.1186/s13063-020-04358-3 (PMC7273688; doi:10.1186/s13063-020-04358-3)
Supplement: Supplementary file 6 — Additional file 6: Appendix 3. Eligibility screening worksheet. [file 13063_2020_4358_MOESM6_ESM.doc]

| **Lead author name and Ref ID Number:** | | | |
| --- | --- | --- | --- |
| **Population**:  Eligible: Any population relevant to recruitment or retention in r**andomised** **controlled clinical or health trials^a^**. This could include e.g. patients; their carers; members of the public or healthy volunteers who would or could be eligible for a specified clinical study; health professionals involved in the study (doctors, nurses, therapists, GPs); or clinical study investigators or research managers.  Ineligible: populations related to non-controlled clinical/health trials; or mixed populations where effectiveness or accuracy outcomes are not separable for the eligible and ineligible populations. | Yes  ↓  next question | Unclear  ↓  next question | No  →  EXCLUDE |
| **Intervention:**  At least one **digital approach** for patient recruitment and/or retention^b^ is reported (any approach that is partly or wholly digitally-based that could influence recruitment or retention, e.g. by raising awareness of a recruiting study, helping to identify eligible people, providing reminders to enrolees to attend visits or tests, providing specific information about study benefits, etc.). | Yes  ↓  next question | Unclear  ↓  next question | No  →  EXCLUDE |
| **Comparator:**  Any comparison that can be made with the digital approach to enable a relevant outcome to be estimated, e.g. a parallel reference or comparison group or before-after comparison. Where multiple comparators are present (e.g. a ‘bundle’ of approaches), the outcomes for these must be separable for the digital and non-digital approaches. | Yes  ↓  next question | Unclear  ↓  next question | No  →  EXCLUDE |
| **Outcomes**:  A measure of the effectiveness or accuracy of the intervention for recruitment and/or retention is reported (any one of the following)^c^:   - Recruitment rate - Recruitment accuracy – quantitative - Recruitment accuracy - qualitative - Time to complete recruitment (for part or all of the process) - Retention rate - Retention accuracy | Yes  ↓  next question | Unclear  ↓  next question | No  →  EXCLUDE |
| **Date:**  Eligible: Study published between 2008 and present date. | Yes  ↓  next question | Unclear  ↓  next question | No  →  EXCLUDE |
| **Design:**  Eligible**:** Any primary research study design that aims to evaluate effectiveness or accuracy of recruitment and/or retention strategies.  Ineligible: Reviews (although these should be recorded and used as a source of references) | Yes  ↓  next question | Unclear  ↓  next question | No  →  EXCLUDE |
| **Final Decision** | **INCLUDE** | **UNCLEAR**  **(Discuss)** | **EXCLUDE** |

**Notes**

^a^ Only include study if the host trial is described as an RCT. However, if not explicitly defined as an RCT but you suspect that it is likely to be randomised (e.g. a comparative cancer treatment clinical trial) then include and state the rationale in the screening log.

^b^ Retention is defined as the number or proportion of patients who:

- are included in the final analysis of a study; OR
- are included any other key analysis in the study if not conducted at the study end; OR
- participate in a specified study visit (e.g. appointment or test) that is instrumental to the analysis (e.g. if numbers analysed are not reported but it’s stated that only those who attend such visits/tests are included in the analysis).

It is NOT:

- A measure of adherence or compliance with a study intervention or protocol (unless data on retention, as defined above, are also given)

^c^ **Examples of recruitment and retention outcomes**

| **Outcome measure** | **Examples** |
| --- | --- |
| Recruitment rate | The proportion of the intended number of participants enrolled in the study. |
| Recruitment accuracy – quantitative | The proportion of participants included in a study accurately meeting study inclusion criteria, as assessed by sensitivity, specificity and/or area under the curve estimates  or, the number of relevant studies identified by a digital tool for a particular patient (i.e. matching the study inclusion criteria to the patient's characteristics) |
| Recruitment accuracy - qualitative | Descriptive similarity of the characteristics of the recruited participants against the study eligibility criteria |
| Time to complete recruitment (for part or all of the process) | Time to identify eligible patients |
| Retention rate | The proportion of recruited participants who remained in the study at the end (for final follow-up assessment) |
| Retention accuracy | Qualitative (descriptive) representativeness of study completers compared to the recruited population |
