# Supplementary material for: Digital tools for the recruitment and retention of participants in randomised controlled trials: a systematic map
Source: Trials. 2020 Jun 5;21:478. doi: 10.1186/s13063-020-04358-3 (PMC7273688; doi:10.1186/s13063-020-04358-3)
Supplement: Supplementary file 7 — Additional file 7: Appendix 4. List of map keywords with explanations and comments. [file 13063_2020_4358_MOESM7_ESM.docx]

**Appendix 4 List of map keywords with explanations and comments**

| **KEYWORD (OR PHRASE)** | **EXPLANATION / COMMENT** |
| --- | --- |
| First author and reference ID number | Administrative data |
| Publication year |  |
| Digital approach name/identifier if >1 approach per study | This is used to identify the different digital approaches where there is more than one approach reported in an article |
| **Publication type** |  |
| Extended abstract | Publication source data |
| Academic journal |  |
| Agency or institution report |  |
| Book |  |
| Book chapter |  |
| Thesis or dissertation |  |
| **Location** |  |
| Country 1 name | Geographical location data |
| Country 2 name |  |
| Country 3 name |  |
| Multinational > 3 countries |  |
| **Evidence type (evaluation design)** |  |
| Experimental - randomised | This refers to the design of the study evaluating the digital recruitment or retention approach i.e. the **primary evaluation study** |
| Experimental - non-randomised |  |
| Observational study or retrospective analysis |  |
| Simulation study |  |
| Other (specify in comment) |  |
| **Purpose of digital approach for recruitment** | These are general classifications of **approaches** which may use one or more **digital tools** (see below) |
| Raise awareness of the host trial | Any digital approach to raise awareness of a trial to potential participants, e.g. via a study website, or advert placed within an App. This keyword can also be used to describe when RCTs are publicised to researchers, health professionals and others who, in turn, can raise awareness of the trial with potential participants. A passive way to expose a study to a patient. |
| Assist people to identify specific trials they may join | Any digital approach that helps potential participants find trials they may join, e.g. a searchable website of health studies. An active way for patients to find relevant studies. |
| Assist study personnel/health professionals to identify eligible study participants | Any digital approach that helps study investigators or clinicians to identify eligible participants, e.g. automated interrogation of patient registries or health records. |
| Obtain participant informed consent | e.g. eConsent tool |
| Other | Any approaches not fitting the above descriptions |
| **Purpose of digital approach for retention** | These are general classifications of **approaches** which may use one or more **digital tools** (see below) |
| Prompts/reminders to attend or complete tasks | Prompts/reminders to attend study appointments or complete outcome assessments or adhere to therapy. These may be delivered by text, email, smartphone App etc. (NB adherence is not a final outcome – it is only assessed if retention is also reported). |
| Communication to maintain engagement with the trial | e.g. study newsletter, or targeted message designed to maintain awareness of the study |
| Automated collation of existing data | e.g. tools for harvesting data from laboratory or electronic patient record systems |
| Digital capture of new data | e.g. Apps, computer programs or messaging systems to record or share outcomes |
| Other (specify in comment) | Any approaches not fitting the above descriptions |
| **Type of digital tool (retention or recruitment)** | These are the tools that can be used as part of the **digital approaches** (see above). If a study compares more than one digital tool then keyword them all here. |
| Internet site for recruitment | e.g. websites or trial directories that publicise a study to potential participants |
| Internet site for retention | e.g. websites that inform enrolees about a study to promote retention |
| Internet forum for recruitment | An internet forum is a website where people can hold discussions about particular topics by posting messages. These differ to chat rooms, where people can have real-time, more interactive discussions. |
| Internet forum for retention |  |
| Internet pop-up adverts for recruitment | A 'pop-up' is a window that suddenly appears on the screen when a person is using a website |
| e-mail for recruitment |  |
| e-mail for retention |  |
| Automated identification of trials for which people are potentially eligible, for recruitment | A system where trials' eligibility criteria have been encoded and therefore the system automatically evaluates for which trial(s) people may potentially be eligible, based on the person's characteristics (e.g. diagnosis, blood test results, gender). The system may be used either by health care professionals or people themselves to search for trials for which they are potentially eligible. |
| Automated screening method used to identify potential participants, for recruitment | An automated screening algorithm that is used to identify potentially eligible participants for a trial. These are automated screening methods. Automatic database queries using a trial's eligibility criteria are run to identify people listed in the database that potentially match the criteria. These systems may be used as part of a pre-screening process in a trial. Some of these systems may also automatically notify health care professionals or study personnel that a patient is potentially eligible for a trial. |
| Digital lecture/presentation (e.g. Powerpoint) for recruitment |  |
| Digital lecture/presentation (e.g. Powerpoint) for retention |  |
| Other computer program for recruitment | Any computer software not covered elsewhere in this list that promotes recruitment |
| Other computer program for retention | Any computer software not covered elsewhere in this list that promotes retention |
| Social media for recruitment | e.g. Facebook, Twitter, YouTube, Instagram, etc |
| Social media for retention |  |
| Crowdsourcing platform for recruitment | e.g. Amazon Mechanical Turk |
| Automated phone call for recruitment | e.g. automated phone survey of patient eligibility |
| Automated phone call for retention | e.g. automated phone reminders for attending a study assessment visit or for taking medication |
| Virtual assistant/gadget for retention | e.g. Amazon Echo |
| Chatbot for recruitment | A chatbot carries out a conversation with a person (or sometimes another chatbot) through text or auditory means, and is run by a computer program or artificial intelligence |
| Chatbot for retention |  |
| Virtual snowballing for recruitment | An explicit request to potential participants to digitally share information on the study, e.g. using their email or social media, thereby propagating the information to a wider number (i.e. reach) of potential participants (“snowballing”) |
| Instant messaging or text messaging for recruitment | e.g. WhatsApp, WeChat, Messenger |
| Instant messaging or text messaging for retention |  |
| Smartphone or tablet App for recruitment | Refers to Apps that are specifically intended or tailored to address recruitment or retention.  Record this keyword if the study specifically uses a named App to deliver the digital approach. For example, if the study specifically uses the Facebook App this would be relevant (record both "Smartphone / tablet App" and "Social media" keywords). But if the study uses Facebook without mentioning the App, choose the "Social media" keyword only (since Facebook can be accessed by other non-App means e.g. internet).  In general this item does not cover conventional apps that are used routinely for accessing items that are already in the list above - e.g. Facebook App, email App, text messaging App, Internet browser App, MS Office Apps, etc would not be eligible for this keyword, unless the App itself is considered integral for delivering the digital approach. |
| Smartphone or tablet App for retention |  |
| Smartphone or tablet other use for recruitment (specify in comment) | Where a smartphone/tablet is used for recruitment or retention in a way other than those already listed  The type of use should be noted (NB this is recorded separately – not listed in the database) |
| Smartphone or tablet other use for retention (specify in comment) |  |
| Video for recruitment |  |
| Video for retention |  |
| Television or radio for recruitment |  |
| Other (specify) | Any digital tools that are not covered above. Online adverts that are not delivered through social media, pop-ups or any of the above categories will be recorded here. This may include unspecified internet adverts, online banner adverts and online adverts on the Google Adwords system. |
| **Non-digital tools included in the digital approach** |  |
| Incentives for recruitment (specify in comment) | The type of incentive should be noted (NB this is recorded separately – not listed in the database)  If the ‘type of intervention’ is ‘combined digital & non-digital approaches’ or if the ‘type of comparator’ is ‘combined digital and non-digital approaches’ or ‘no formally defined comparator’, record all the non-digital approaches used here |
| Incentives for retention (specify in comment) |  |
| Mailouts for recruitment |  |
| Flyers for recruitment |  |
| Non-digital (i.e. human-mediated) phone call for recruitment |  |
| Non-digital (i.e. human-mediated) phone call for retention |  |
| Word of mouth for recruitment |  |
| Other (specify in comment) |  |
| **Type of intervention** |  |
| Single digital tool | A classification to identify whether individual tools or “bundles” of digital and/or non-digital tools were evaluated in the intervention approach |
| Multiple combined digital tools |  |
| Combined digital & non-digital tools |  |
| **Type of comparator** |  |
| Single digital tool | A classification to identify whether individual tools or “bundles” of digital and/or non-digital tools were evaluated in the comparator approach |
| Multiple combined digital tools |  |
| Single non-digital tool |  |
| Multiple combined non-digital tools |  |
| Combined digital & non-digital tools |  |
| Other comparison type (specify in comment) |  |
| No formally-defined comparator - but a multi-component approach with results separable for the components | No formally-defined comparator is present but there are multiple components to the approach for which outcomes for digital and/or non-digital components are reported separately, enabling some (unplanned) digital/non-digital comparisons |
| **Effectiveness outcomes assessed** | These are the effectiveness outcomes of the **primary evaluation study** of the digital tool itself, rather than the **host trial** that participants are being recruited to/retained in |
| Recruitment rate | The proportion of the intended number of participants enrolled in the trial |
| Recruitment accuracy - quantitative | The proportion of participants identified/ recruited for a trial who accurately meet the trial inclusion criteria, e.g. as assessed by sensitivity, specificity and/or area under the curve estimates. Or, the number of relevant trials identified by a digital tool for a particular potential participant (i.e. matching the study inclusion criteria to the person's characteristics). |
| Recruitment accuracy - qualitative | Descriptive similarity of the characteristics of the identified/ recruited participants against the trial eligibility criteria |
| Time to complete recruitment (for part or all of the process) |  |
| Recruitment reach | The population of potentially eligible and accessible participants for the host trial, as measured by how many potentially eligible participants were identified, approached, referred to, and/or made contact with study personnel about the trial |
| Retention rate | The number or proportion of patients who: are included in the final analysis of a study; OR are included any other key analysis in the study if not conducted at the study end; OR participate in a specified study visit (e.g. appointment or test) that is instrumental to the analysis (e.g. if numbers analysed are not reported but it’s stated that only those who attend such visits/tests are included in the analysis). It is NOT a measure of adherence or compliance with a study intervention or protocol (unless data on retention, as defined above, are also given). |
| Retention accuracy - qualitative | Qualitative (descriptive) representativeness of study completers compared to the recruited population |
| Other (specify in comment) |  |
| **Other outcomes assessed** |  |
| Attitudes towards use of the tool | This can be about the actual or intended use of a tool (e.g. user satisfaction with a video e-consent tool, or public concern about privacy issues associated with a targeted email campaign) |
| Study participant satisfaction | The (potential) participant’s satisfaction with being identified/ recruited or with participating in the study |
| Study personnel satisfaction | Trial investigator or clinical personnel satisfaction with the participant identification / recruitment process (e.g. impact on the trial recruiter (including research nurse, GP etc.) workload or other concerns with the process) |
| Cost of recruitment or retention | Any costs associated with the use of the digital tool (could include e.g. capital costs, revenue, time cost to develop or configure the tool for a study, or to reconfigure it for another study) |
| Efficiency of tool | Efficiency as expressed as a return on investment in the use of a digital tool (e.g. cost per additional participant recruited and/or retained) |
| Other (specify in comment) |  |
| **Type of host trial** |  |
| RCT | This distinguishes between host trials which were clearly reported as being RCTs and those which were not clearly reported but which the review team deemed were likely to have been RCTs based on the available information |
| Clinical trial (not explicitly stated as an RCT) |  |
| **Health topic(s) under study** |  |
| Bone and joint diseases |  |
| Brain and nervous system diseases |  |
| Cancers |  |
| Ear diseases |  |
| Endocrine, nutritional and metabolic conditions | e.g. diabetes, obesity, metabolic disorders |
| Eye diseases |  |
| Genito-urinary system diseases |  |
| Health promotion and public health (select specific topic below) | e.g. screening programmes, vaccination programmes |
| *Smoking cessation or tobacco control* |  |
| *Sexual health promotion* | Note that this category includes studies evaluating HIV prevention interventions, as well as other sexual health promotion activities. |
| *Physical activity promotion* |  |
| *Healthy eating* |  |
| *Alcohol misuse* |  |
| *Cardiovascular health promotion* |  |
| *Lifestyle interventions for diabetes prevention* |  |
| *Lifestyle interventions for weight gain or obesity prevention* |  |
| *Health checks or screening* |  |
| *Falls and fracture prevention in older adults* |  |
| *Cancer chemoprevention* |  |
| Infectious diseases |  |
| Mental health |  |
| Respiratory diseases |  |
| Skin diseases |  |
| Digestive system diseases |  |
| Circulatory system diseases |  |
| Maternal health and pregnancy |  |
| Other (specify in comment) |  |
| **Population studied** |  |
| Minority or under-served population | As defined by the study author, but could include e.g. gender, ethnicity, sexuality, disability, difficult to reach populations |
